# TALEN-Based Mutagenesis of Lipoxygenase LOX3 Enhances the Storage Tolerance of Rice (*Oryza sativa*) Seeds

**DOI:** 10.1371/journal.pone.0143877

**Published:** 2015-12-07

**Authors:** Lei Ma, Fugui Zhu, Zhenwei Li, Jianfu Zhang, Xin Li, Jiangli Dong, Tao Wang

**Affiliations:** 1 State Key Laboratory of Agrobiotechnology, College of Biological Sciences, China Agricultural University, Beijing, China; 2 State Key Laboratory of Cotton Biology, Institute of Cotton Research of CAAS, Anyang, China; 3 Rice Research Institute, Fujian Academy of Agricultural Sciences, Fuzhou, China; Wuhan University, CHINA

## Abstract

The deterioration of rice grain reduces the quality of rice, resulting in serious economic losses for farmers. Lipoxygenases (LOXs) catalyze the dioxygenation of polyunsaturated fatty acids with at least one cis,cis-1,4-pentadiene to form hydroperoxide, which is a major factor influencing seed longevity and viability. Recently, genome editing, an essential tool employed in reverse genetics, has been used experimentally to investigate basic plant biology or to modify crop plants for the improvement of important agricultural traits. In this study, we performed targeted mutagenesis in rice using transcription activator-like effector nucleases (TALENs) to improve seed storability. A modified ligation-independent cloning method (LIC) was employed to allow for the quick and efficient directional insertion of TALEN monomer modules into destination vectors used in plants. We demonstrated the feasibility and flexibility of the technology by developing a set of modular vectors for genome editing. After construction and validation, the TALEN pairs were used to create stable transgenic rice lines via *Agrobacterium*-mediated transformation. One heterozygous mutant (4%) was recovered from 25 transgenic NPTII-resistant lines, and the mutation was transmitted to the next generation. Further molecular and protein level experiments verified LOX3 deficiency and demonstrated the improvement of seed storability. Our work provides a flexible genome editing tool for improving important agronomic traits, as well as direct evidence that Lox3 has only a limited impact on seed longevity.

## Introduction

Rice (*Oryza sativa*) is an important food crop that feeds more than half of the world’s population. However, during storage, rice gradually loses its viability and acquires a stale flavor (referred to as rice grain deterioration), causing serious economic losses for farmers. In addition to environmental stresses, genetic factors also influence rice seed longevity and storage tolerance.

Previous studies have shown that lipid metabolism [1, 2], protein and DNA damage [3–5], and heat shock factors [6, 7] impact seed longevity. Lipid metabolism is an important contributory factor to lipid membrane degradation and seed deterioration, thus affecting seed longevity. Arabidopsis phospholipase D (PLDα1) negatively regulates seed quality and viability, and PLDα1-deficient seeds show higher germination rates and accumulate more unsaturated fatty acids than wild-type seeds after artificial aging treatments [1]. Arabidopsis VTE1 and VTE2, two enzymes involved in vitamin E biosynthesis, positively regulate seed longevity, and vitamin E may prolong seed longevity by reducing lipid peroxidation during storage [2]. AtDLAH is a mitochondrial acyl hydrolase that catalyzes the hydrolysis of phospholipids and plays a positive role in Arabidopsis seed viability [8].

Research on rice seed storability is mainly focused on lipoxygenases (LOXs;EC 1.13.11.12). LOX enzymes catalyze the dioxygenation of polyunsaturated fatty acids containing at least one cis,cis-1,4-pentadiene to form hydroperoxide. Based on bioinformatics analysis, the rice genome encodes 14 LOX proteins [9], five of which have been cloned and characterized, including three embryo isozymes (LOX1, LOX2, and LOX3) [10–12] and HI-LOX [13, 14]. LOX1, LOX2 and LOX3 have distinct functions in rice seed longevity, while others are involved in biotic stress responses. Compared with wild type seeds, *OsLOX2* over-expressing seeds germinate faster under normal conditions but show lower germination rates after aging treatments. RNAi lines perform conversely, suggesting that LOX2 is involved both in seed germination and longevity [12]. LOX3 protein was purified in 1986, and the gene encoding LOX3 was cloned in 2008. A point mutation from G to A in an allele of *Os03g0700400* in the variety ‘Daw Dam’ results in the premature stop of translation, producing a LOX3-null mutant [15]. The biochemical function, subcellular localization, and expression pattern of LOX3 have been characterized, and its effect on seed longevity was also evaluated [11]. Antisense suppression of *lox3* was also investigated and was demonstrated to increase the viable storage time of rice seeds [16].

Genome editing enables researchers to precisely manipulate a gene or gene family and is dependent on engineered endonucleases such as zinc finger nucleases (ZFNs), transcription activator-like effector nucleases (TALENs), and the cluster regularly interspaced short palindromic repeats (CRISPR)/Cas system [17]. These nucleases can generate DNA double-strand breaks (DSB) at specific sites, which triggers DNA damage repair via either non-homologous end joining (NHEJ) or homologous recombination. NHEJ is error-prone and can introduce mutations such as small insertions and deletions, resulting in gene inactivation. Genome editing technology has been used in basic science research and molecular breeding research in agriculture. The *Os11N3* promoter was successfully edited with high efficiency using TALENs to produce *Xanthomonas oryzae*-resistant rice [18], and three homoallelic *MLOs* were simultaneously edited in hexaploid bread wheat, overcoming genetic redundancy and producing wheat with a broad-spectrum resistance to powdery mildew [19]. Targeted mutagenesis of the fatty acid desaturase 2 gene family via TALENs was used to create high oleic acid soybean varieties [20]. TALENs have also been used in the creation of fragrant rice by mutating *OsBADH2*, which encodes betaine aldehyde dehydrogenase [21].

Due to their longer recognition sites, TALENs may cause fewer unwanted off-target effects than ZFNs and the CRISPR/Cas9 system. However, it is laborious and time consuming to construct a ready-to-use destination vector for TALEN [22], severely limiting its applications in research and molecular breeding. Although several methods such as Golden Gate cloning [23] have been developed to assemble repeats [24], specifically designed destination vectors remain scarce, especially in plant research. In this study, we use a modified ligation-independent cloning (LIC) method to allow for the quick and efficient directional insertion of TALEN monomer modules into transformation vectors for genome editing in plants. Taking advantage of this method, we obtained the LOX3 mutant lines. Further genetic and protein experiments were carried out to verify LOX3 deficiency and to demonstrate the improvement of seed storability. Our work provides a flexibility and powerful tool for improving agronomic traits.

## Results

### Binary destination vector assembly

We have developed a convenient method to obtain ready-to-use destination vectors for genome editing based on TALEN. T-DNA consists of two TALEN expression cassettes containing the respective plant promoters. A modified LIC cloning method was designed for the quick and efficient directional insertion of TALEN monomer modules into destination vectors.

**Fig 1:**
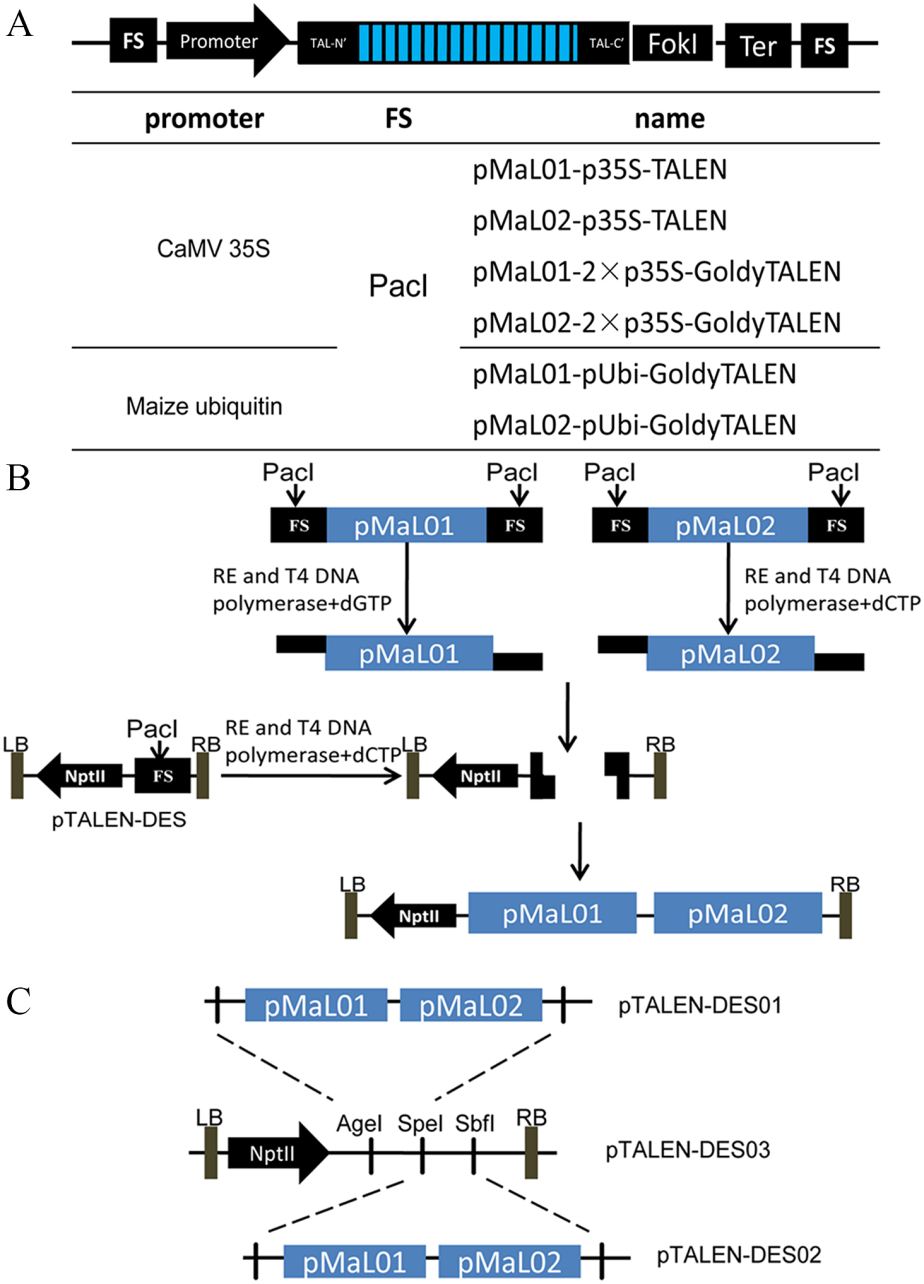
Schematic representation of the modular vectors generated and the assembly of binary destination vectors. **(A)** The modular vectors were designed for overexpression in plants, consisting of FS (flanking sequences), promoter, TALE, FokI and Ter (terminator). The expression of the TALEN cassettes of the modular vectors is driven by the promoters specified below. The CaMV35S promoter (double-enhancer version) and maize ubiquitin promoter were used for dicot or monocot plant transformation. A PacI endonuclease site was embedded into the FS used to obtain DNA fragments. The typical protein structure of an artificial TALEN composed of a repetitive DNA binding domain flanked by an N-terminal and a C-terminal was followed by the cleavage domain of FokI nuclease. The GoldyTALEN scaffold with N- and C-terminal truncations (N152/C63) fused with enhanced heterodimeric (ELD, KKR mutations) FokI domains is also provided. (**B**) A modified LIC method was implemented to enable high-throughput cloning of constructs for genome editing. The binary destination vector (pTALEN-DES) and DNA fragments containing TALEN cassettes were obtained using the restrictive endonuclease (RE) PacI and were processed by T4 DNA polymerase to create specific 12 nucleotide single-stranded overhangs. After annealing, a circular plasmid was generated in *Escherichia coli* cells without the use of T4 DNA ligase. (**C**) A schematic illustrating the construction of the binary destination vector containing four TALEN cassettes. RE, Restriction endonuclease; LB and RB, left border and right border of T-DNA; NptII, neomycin phosphotransferase (KanR); PacI, AgeI, SpeI, SbfI, recognition sequences of the restriction enzymes PacI, AgeI, SpeI and SbfI, respectively.

First, two original donor vectors, pMAL01 and pMAL02, were constructed to contain the respective full-length TALEN backbone sequences. To achieve a higher level of gene-modification and to reduce off-target effects, we also generated a GoldyTALEN scaffold fused with enhanced heterodimeric (ELD, KKR mutations) FokI domains. The GoldyTALEN scaffold, with N and C terminal truncations, results in a much higher induced mutation rate than the parental vectors [25]. The adoption of the same promoters for the T-DNA results in certain transgene silencing effects [26]; thus, different promoters were recommended. The CaMV35S promoter (p35S), a double-enhancer version of the CaMV35S promoter (2×p35S), and the maize ubiquitin promoter (pUbi) were used in dicotyledonous or monocotyledonous plant transformations with their respective terminator sequences (Fig 1A). In addition, we included several endonuclease cleavage sites to allow for promoter replacement in future studies. Flanking sequences (FS) containing PacI restriction enzyme recognition sites for generating compatible termini were incorporated into the destination vector and both ends of the TALEN cassette. Proofreading DNA polymerases, such as T4 DNA polymerase, resect the 3′ ends of the double-stranded DNA molecules until they meet a deoxyribonucleotide triphosphate, at which point digestion is terminated. Well-defined ssDNA overhangs of 12 bp at the termini of the dsDNA molecules were generated. In this study, dG and dC bases were placed at opposite dsDNA ends of the FS elements in pMAL01 and pMAL02.

Second, for typical heterodimeric target sites, the TALEN monomer was constructed using the Golden-Gate method [23]. These plasmids were then digested with restriction enzymes (PacI) followed by the resection of the 3′ ends of the double-stranded DNA molecules by T4 DNA polymerase and terminated by the corresponding deoxyribonucleotide triphosphate (dG or dC). Well-defined single-stranded overhangs of 12 nucleotides were obtained in the plant destination vector (pTALEN-DES01/02) and on the DNA inserts (TALEN cassettes). After annealing, mixtures of vector and DNA inserts were transformed into competent *E*. *coli* cells without the use of T4 DNA ligase.

Finally, a circular plasmid (pTALEN-DES-MaL1-MaL2) was obtained within *E*. *coli* cells through the formation of covalent bonds at the vector-insert junctions. Restriction digestion screening confirmed that the plasmids yielded were all true recombinants. pTALEN-DES01/02 was designed for use with the Golden Gate TALEN kit, replacing the pTAL vector. We were able to obtain a plasmid immediately following Golden Gate assembly, which is simple and highly efficient. It is worth mentioning that we included unique restriction sites (AgeI, SpeI, and SbfI) in pTALEN-DES01/02. Through traditional digestion and ligation reactions, four TALEN expression cassettes were assembled for pTALEN-DES03. In summary, we established a cost-efficient and high-throughput vector system designed for use with the Golden Gate TALEN kit, which is optimized for genome editing in plant systems.

### Design and validation of TALEN pairs targeting endogenous lipoxygenase LOX3

**Fig 2:**
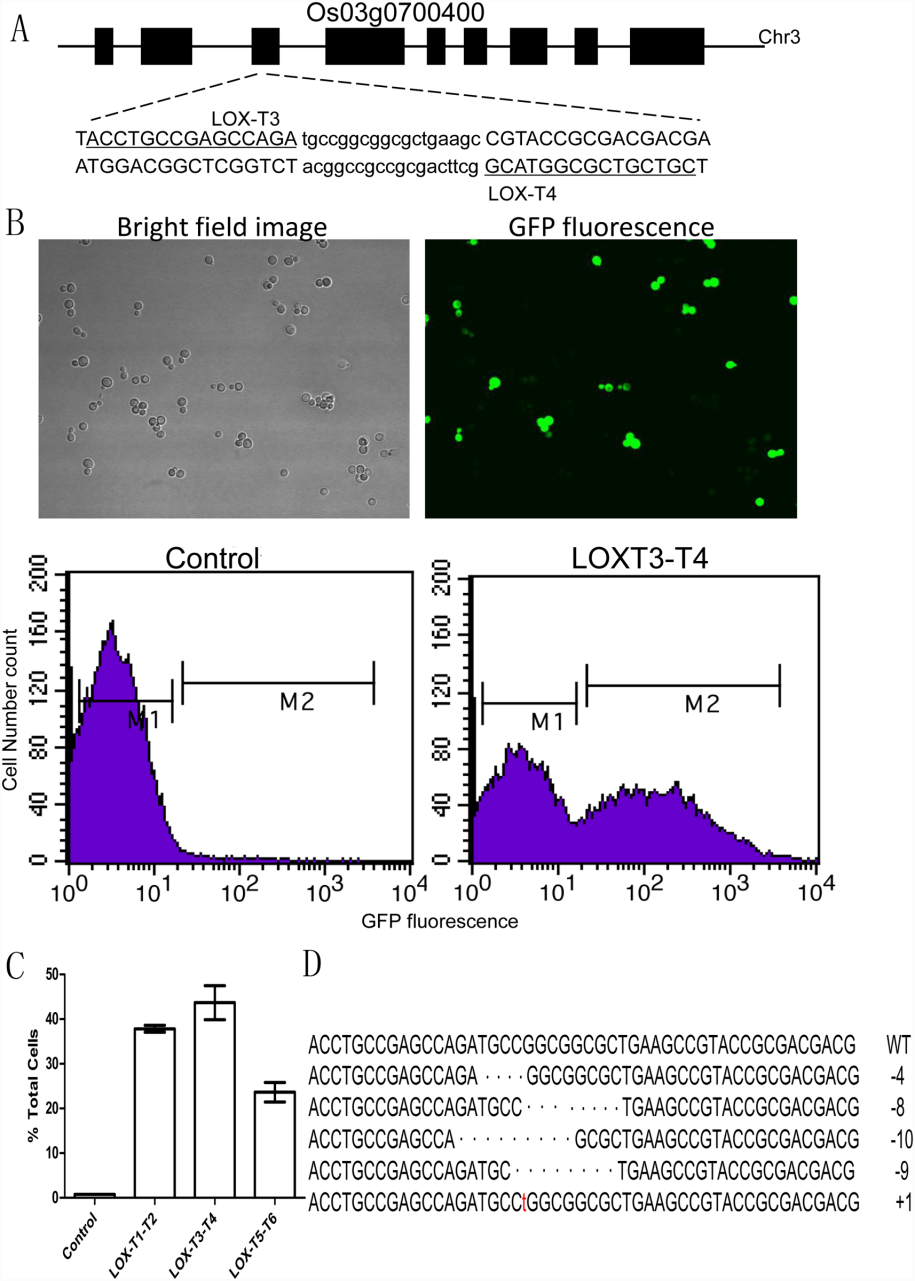
Design and validation of TALEN pairs targeting the endogenous lipoxygenase LOX3. **(A**) The structure and sequence of the lipoxygenase LOX3 (*Os03g0700400*), showing the binding sites of the TALEN pairs (uppercase). (**B**) A transient assay for the activity of the TALEN pairs in yeast. GFP fluorescence was observed after two overlapping gfp fragments recovered a functional GFP. Representative images of cells treated with TALEN pairs are shown. The proportion of YFP-positive cells in each sample was quantified using flow cytometry. (**C**) The activity of custom TALEN pairs was assessed by determining the percentage of GFP-positive cells. Control, cells introduced by empty vector. Error bars denote the s.d.; n = 3. (**D**) Different types of mutations induced by TALEN pairs in the rice protoplast. Deletions are represented by dashes; inserted bases are in lowercase. The lengths of the insertions (+) or deletions (−) are indicated to the right of the sequences.

Lipoxygenase LOX3 has been reported to play an important role in the acquisition of a stale flavor during rice storage [27]. Full length *LOX3* (Os03g0700400) measures 4402 bp, including nine exons and eight introns, and is located on chromosome 3. Three different TALEN pairs were designed to target unique sequences in exon 3 or exon 4 of the *LOX3* gene (Fig 2A). Target sites, which are required to have a T nucleotide at the start of each sequence, were identified using the publicly available TALE-NT program. The number of repeats in each TAL effector array ranged from 15 to 20, and the length of the spacers within the target sites were between 15 and 20 bp (S1 Table). To test activity and specificity, the TALEN pairs targeting the *lox3* gene were assessed using a single-strand annealing (SSA)-mediated yeast assay in which green fluorescent protein (GFP) activity served as an indicator of DNA cleavage. In this assay, the target plasmid contained a GFP reporter gene with two partially overlapping GFP fragments. The TALEN target site is set in the middle of the duplication. The target plasmid and TALEN expression pairs are brought together in the same cell, and when a double-stranded DNA break occurs at the target site, it is repaired through single-strand annealing between the duplicated sequences, creating a functional GFP gene whose expression can be quantified by counting the number of cells emitting green fluorescent light via flow cytometry (Fig 2B). The TALEN pair LOX-T3 and LOX-T4 exhibited the highest activity and was therefore chosen for use in validation experiments in rice protoplasts (Fig 2C).

The pFUS_A and pFUS_B modules, constituting LOX-T3 and LOX-T4, respectively, were assembled directly into a TALEN cassette with pMaL01-P35S-TALEN and pMaL02-P35S-TALEN using the Golden Gate method. After a round of stacking (Fig 1B), the transformation vector pTALEN-DES-T3-T4 was generated and introduced into rice protoplasts for transient assay. Genomic DNA was prepared from the samples, and the DNA fragments encompassing the target site were amplified by PCR and cloned for DNA sequencing. Mutations within the target’s spacer sequence confirmed the presence of NHEJ-induced mutations and further validated the TALEN pair’s activity (Fig 2D).

### Screening and identification of the LOX3-knockout mutant

**Fig 3:**
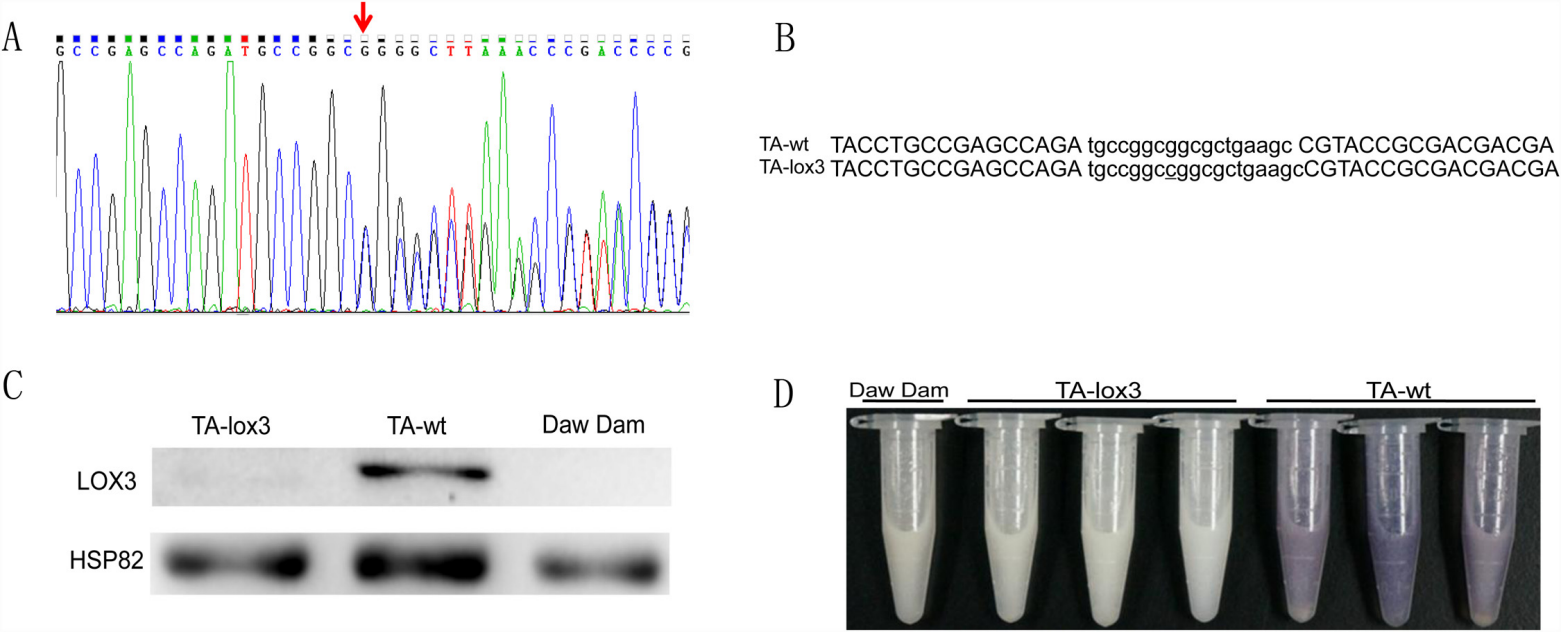
Screening and verification of TALEN-induced LOX3 mutant lines. **(A**) Screening TALEN-induced T0 line by Sanger sequencing. The insertion was introduced at the target site in the *lox3* gene, giving rise to a double sequence.(**B**) The genotypes of T0 lines induced by TALEN pairs.(**C**) Western blot analysis of LOX3 expression levels in rice seeds. The anti-LOX3 monoclonal antibody was used (upper bands), and HSP82 is the reference protein (lower bands). Daw Dam is a known LOX3-null variety and served as a negative control.(**D**) Verification of LOX3 activity by the KI–I2 starch staining method. A dark purple color was formed by LOX3-normal rice seeds, whereas no color was observed in LOX3-null lines.

Through *Agrobacterium*-mediated rice transformation with the vector pTALEN-DES-T3-T4, a total of 25 transgenic NPTII-resistant T0 plants were recovered in the ‘Nipponbare’ background. All T0 transgenic plants were evaluated by PCR; however, only one was found to contain a mutation at the target site. As shown in the Sanger sequencing chromatographs, insertions introduced at the site resulted in complicated peaks (Fig 3A). Sequencing revealed that a C base was inserted at the spacer between the target sites (Fig 3B). To determine whether the mutation could be transmitted stably to the next generation, this T0 mutant line was self-pollinated, and 100 T1 lines were randomly selected to identify mutations via direct sequencing. The results showed that 29 (29.0%) lines were homozygous mutants (TA-lox3 mutant), 45 (45.0%) lines were heterozygous (i.e., DNA sequencing results showing double peaks), and the remaining 26 (26.0%) lines were wild type (TA-wt line), conforming to Mendel’s law of segregation.

A single base insertion resulted in a frame shift that led to the premature termination of translation, resulting in a protein of 248 amino acids that probably has no function. To test the deficiency of LOX3 mutant seeds at the protein level, we performed western blotting and enzyme activity detection experiments. Using the monoclonal antibody anti-LOX3 [16], we detected a LOX3 band in the TA-wt seeds but no band in the TA-lox3 mutants or ‘Daw Dam’ seeds, demonstrating the absence of LOX3 in the TA-lox3 mutants (Fig 3C). Next, the KI–I2 starch staining method, which is based on the enzyme activity of lipoxygenase, was used to determine the absence of LOX3 in rice seeds. As is shown in Fig 3D, TA-wt embryos stained a dark purple color, while TA-lox3 mutants and ‘Daw Dam’ embryos were colorless. This is direct evidence for the lack of functional LOX3 in the TA-lox3 mutants.

To obtain rice lines that contain LOX3 mutations without TALEN transgenes or NPTII-resistant genes, a PCR-based assay was conducted to test the T1 lines. Three primer pairs for the FokI gene, TALE and NPTII gene were designed, and the endogenous LOX3 gene was used as an internal reference. We identified 18 (18.0%) lines with the T-DNA eliminated, indicating that the TALEN construct had segregated during propagation (S1 Fig).

### LOX3 knockout mutants show improved seed storability

**Fig 4:**
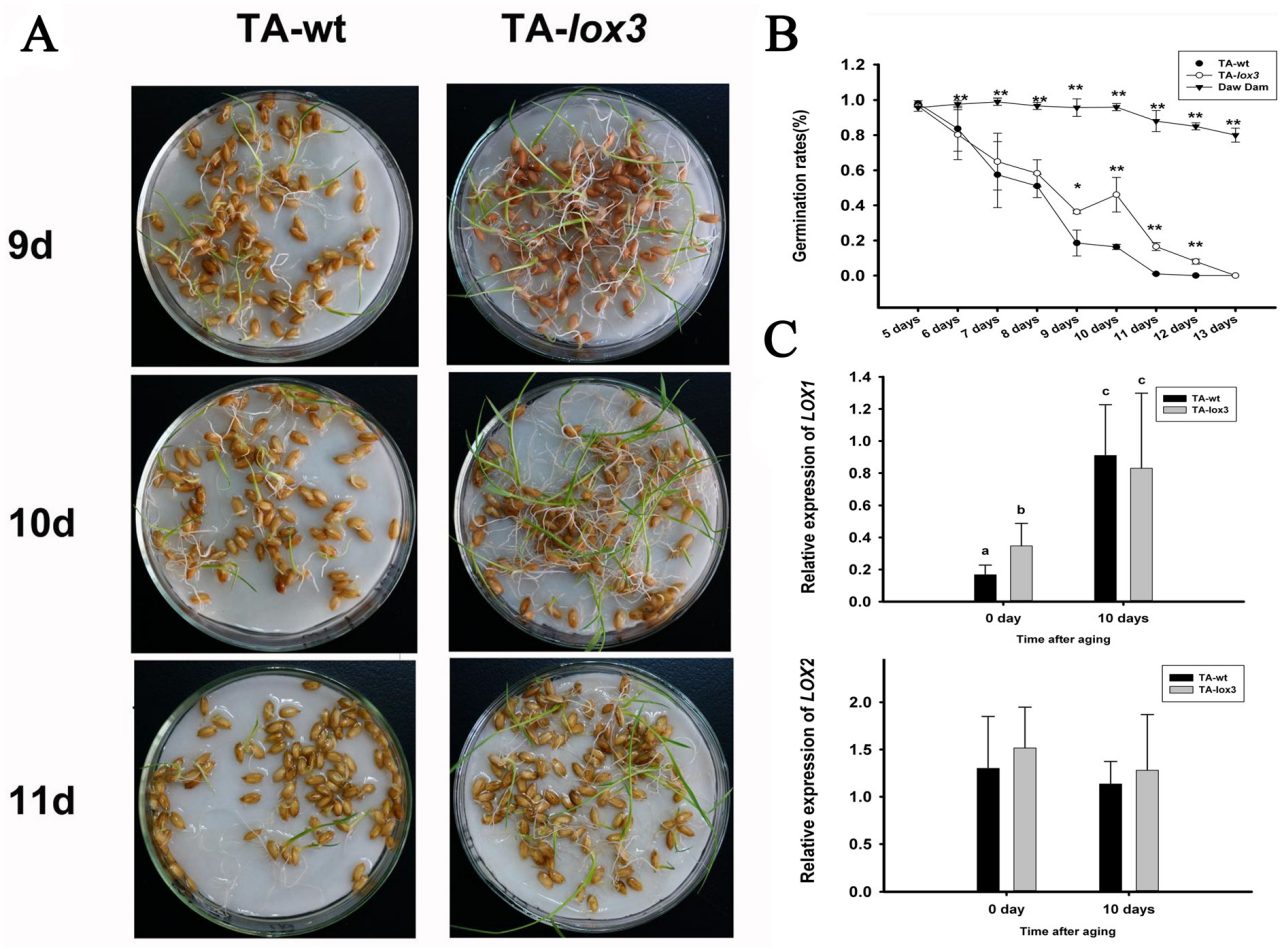
TALEN-induced LOX3-knockout mutant seeds have enhanced seed longevity. **(A)** Representative images of TA-wt seeds and TA-lox3 mutant seeds after 9, 10, and 11 days of aging treatment. (**B)** Changes in the germination rate of TA-wt and TA-lox3 during artificial accelerated aging treatment. (C) qPCR analysis of the gene expression of LOX1 and LOX2 after aging treatment.Values are presented as the mean ± SD of three replicates. A Kruskal and Wallis test was used to determine the significant differences (indicated by letters,α<5%;).

Seed storability is evaluated by determining the germination rate after a long storage time. To shorten the experimental period, researchers often create a simulated environment with high temperature and high humidity to accelerate seed aging in a process referred to as artificial aging. Previous studies have shown that LOX3 promotes lipid peroxidation, and hence plays a negative role in seed longevity [11, 16]. We compared the longevity of the T3 lines of TA-lox3 mutant seeds with TA-wt seeds and Daw Dam using an artificial aging treatment (Fig 4A). Before the aging treatment, the germination rate of the TA-lox3 mutant and TA-wt seeds were compared and found to exhibit no significant difference (P > 0.05), indicating that LOX3 has no effect on seed germination under normal conditions. TA-wt and TA-lox3 seeds exhibit similar trends under artificial aging, with no significant differences after 6 days of aging. However, the TA-lox3 mutant had a higher germination rate than TA-wt after 9- and 11-day aging treatments (P<0.01 Fig 4B), and even after a 12-day treatment, the TA-lox3 mutant had a germination rate of 8%. As is shown in Fig 4B, the Daw Dam had a significantly higher germination rate of ~80% after 13 days of accelerated aging, while the germination rate of TA-lox3 mutants was reduced to almost zero. The result obtained from this loss-of-function transgenic plant, rather than an RNAi line, provides direct evidence that Lox3 exerts only a limited impact on seed longevity, and does not play a significant role consistent with previous reports [11]. Both *r9-LOX1* and *OsLOX2* were also proved to have the capacity to maintain rice grain quality and viability during storage [12, 28]. Decreased overall lox expression, involving LOX1, LOX2 and LOX3, could extend seed longevity significantly [16, 29]. The real-time PCR was employed to check the gene expression of LOX1 and LOX2 after aging treatment. As is shown in (Fig 4C), the transcript level of r9-LOX1 gene of both TA-wt and TA-lox3 were up regulated significantly (P<0.01). But the expression levels of LOX2 did not show any significant differences as compared to the untreated TA-wt and TA-lox3 (P>0.05). The result indicated that LOX1 plays a dominant role in the absence of LOX3.

The fatty acid content of rice seeds in TA-lox3 and TA-wt seeds was measured by gas chromatography (GC) to study the effect of LOX3 deficiency on peroxidation (Table 1). The unsaturated fatty acid, especially linoleic acid (LA), is the substrate of LOX3, and LA levels declined markedly after artificial aging treatment in both TA-wt and TA-lox3 seeds (Table 1). However, there was no significant difference in LA content between TA-lox3 and TA-wt seeds before and after aging treatment. Taken together, unsaturated fatty acid peroxidation did occur during aging treatment, but the deficiency of LOX3 did not slow the extent of its peroxidation. Furthermore consistent with previous reports [28], other fatty acids (16:0, 18:0, 18:1) was also observed decrease in TA-wt and TA-lox3 rice seeds after aging treatment.

**Table 1:** Fatty acid content of rice seeds of TA-wt and TA-*lox3* after aging treatment.

Fatty acid	0 day Aging Treatment		10 days Aging Treatment	
	TA-wt	TA-*lox3*	TA-wt	TA-*lox3*
C16:0	6.70±0.332^b^	6.83±0.589^b^	5.81±0.523^a^	6.14±0.375^ab^
C16:1	0.05 ±0.002^a^	0.05±0.008^a^	0.04±0.001^a^	0.05±0.011^a^
C18:0	0.51 ±0.033^ab^	0.54±0.066^b^	0.43±0.031^a^	0.46±0.029^ab^
C18:1	10.40±0.501^a^	9.79±0.430^a^	7.99±0.342^b^	8.44±0.511^b^
C18:2	13.04 ±0.638^c^	12.76±0.963^bc^	11.26±0.566^a^	11.51±0.346^ab^
C18:3	0.53 ±0.022^b^	0.52±0.030^b^	0.44±0.045^a^	0.44±0.006^a^
C20:0	0.14 ±0.003^a^	0.14±0.012^a^	0.12±0.007^a^	0.13±0.007^a^
C22:0	0.10 ±0.004^ab^	0.11±0.012^b^	0.09±0.002^a^	0.09±0.004^a^

The contents of fatty acid are shown in the table. Three independent experiments were performed, data represent means of three replicates±SD. A Kruskal and Wallis test was used to determine the significant differences (data indicatted with the same letter are not significantly different (P<0.05);).

The above results indicated that seed aging is a complex process, and it is not realistic to expect to enhance the storage-tolerance of rice seeds by down-regulating a single gene. Furthermore, RNAi may be a better technique for simultaneous suppression of several genes compared with the targeted-mutation one gene by genome editing in order to enhance seed longevity.

### LOX3 does not affect the agronomic traits of rice

Lipoxygenase plays diverse roles in plant development, pathogen infection, insect attack, and mechanical wounding. However, the goal of molecular breeding is to avoid negative effects on other traits, especially those related to crop development and yield. Additionally, LOX3 is not only expressed in seeds but also in roots, panicles, and stems, with the highest expression in roots [11]. To determine whether the LOX3 mutation has an impact on rice development and yield, we made a general comparison of the main agronomic traits, including height, tiller number, filled grain percentage, and thousand grain weight, between TA-wt and TA-lox3 lines (Table 2). The results showed that there were no significant differences, suggesting that LOX3 deficiency had no effect on the main agronomic traits of rice.

**Table 2:** Agronomic traits of TA-wt and TA-lox3 lines in the field.

	TA-wt	TA-lox3
Height(cm)	110 ± 3.6	108 ± 4.3
Tillers(Panicles) number	14 ± 2.4	13 ± 3.2
Panicle length (cm)	17.2 ± 0.99	16.8 ± 1.56
Leaf length (cm)	35.6 ± 3.2	33.7 ± 3.7
Flag leaf width (cm)	1.49 ± 0.07	1.49 ± 0.08
Number of grain per plant	1237 ± 291	1103 ± 189
Number of filled grain per plant	1052 ± 282	959 ± 193
Filled grain percentage (%)	84 ± 4	86 ± 5
Thousand grain weight (g)	24.4 ± 0.7	24.2 ± 1.7
Grain weight per plant (g)	25.8 ± 7.0	23.2 ± 5.3

Values are the mean ± SD of three replicates (n = 20). Mean without any denote indicated that there was no significant difference at p<0.05.

## Discussion

In recent years, precise genome editing has emerged as a tool for the functional characterization of plant genes and the genetic improvement of agricultural crops. Several reports describing applications of these genome editing systems in different plants such as *Arabidopsis*, tobacco, and rice have been published, but there are only a few successful examples of improved crop quality. In addition, genome editing technology has a unique advantage for GMO safety; segregating plants can be genotyped to identify individuals that are homozygous for a mutation and no longer carry the marker gene, similar to the two T-DNA binary system [30].

TALEN repeat assembly methods currently in use include Golden Gate, FLASH assembly, unit assembly, and Iterative Capped Assembly, of which the Golden Gate method is the most convenient and efficient [24]. However, the final expression vector assembled by Golden Gate is usually a yeast or mammalian expression vector containing only one TALEN monomer. Because yeast and mammalian cell transformation and transfection methods are highly developed, co-transformation strategies could solve the need for introducing multiple TALEN monomers into a cell. However, in plant functional genomics and biotechnology research, the need for co-transformation severely limits the application of TALEN technology due to its complexity and inefficiency. Although the linkage of two TALEN monomer coding sequences by a T2A sequence has been reported [24], optimized destination vectors designed for plant expression are needed. This study provides a vector system allowing two or more TALEN cassettes to integrate into T-DNA, which could reduce the vast amount of time spent on molecular cloning. This vector system, combined with the method of pre-assembling trimer and tetramer repeat-variable diresidue domains [31, 32], could markedly reduce the time of vector construction to 3 days from the design of the vector to a ready-to-use plant binary vector.

Only one heterozygous mutant was recovered from 25 transgenic lines, which is lower than reported previously [18, 21]. When the same promoter (35S) was employed for both TALENs on the T-DNA, the expression of each gene was silenced in *Nicotiana benthamiana* by agroinfiltration [33]. However, another study demonstrated that tandem genes expressed from the 35S promoter resulted in comparable levels of TALEN activity, as measured in tobacco protoplasts [34]. Taking into account the higher transformation efficiency of protoplasts than agroinfiltration, we hypothesize that gene silencing may be the cause of low efficiency and that a different promoter should be utilized. Thus, we developed modular plasmids coupled with different promoters. A TALEN backbone with N- and C-terminal truncations (N152/C63) fused to hetero-dimeric FokI cleavage domains was also employed to achieve higher gene modification efficiency and to reduce off-target effects.

We successfully knocked out endogenous LOX3 by TALEN and found that LOX3 knockout seeds exhibited improved storability, providing another example of the use of TALEN to change metabolic pathways for improved grain quality. This is the most direct evidence of the negative role of LOX3 in rice seed longevity, compared with previous studies using overexpression and antisense suppression [11, 16]. Although rice seeds with enhanced longevity have been generated via antisense suppression [16], TALEN has obvious advantages compared with traditional transgenic or hybridization breeding; TALEN can precisely modify a gene in a manner that is stably inherited, in contrast to RNAi gene modifications, which are incomplete and unstable. In addition, modified plants without exogenous transgenes that resemble natural mutants can be obtained, and because breeding TALEN lines does not require several generations, a great amount of time is saved.

All LOX3 null varieties shared a uniform mutation in *Os03g0700400*, suggesting that LOX3 is conserved in rice and that TALEN could be applied to other rice varieties that are frequently used in breeding. However, it is important to stress that rice seed longevity is a complicated quantitative trait that involves several major and minor genes [35]. LOX3 has long been thought to affect seed longevity by catalyzing the peroxidation of linoleic and linolenic acids, but our results show that TA-lox3 seeds did not accumulate more LA after aging treatment, indicating that LOX3 deficiency cannot slow the extent of unsaturated fatty acid peroxidation. This could be due to functional redundancy among the three isoenzymes expressed in the embryo (LOX1, LOX2 and LOX3). These additional two isoenzymes can also catalyze the peroxidation of LA, so the mutation of only LOX3 cannot retard the peroxidation of LA. Although the production of 9-hydroperoxyoctadecadienoic acid (9-HPOD) from linoleic acid was significantly lower in transgenic seeds of antisense LOX3 grains, quantitative real-time PCR showed that the expression levels of LOX1 and LOX2 were also down-regulated significantly [16]. In summary, we speculate that LOX3 may catalyze an unknown metabolic pathway in which the produced oxylipins then regulate the seed longevity. Future studies of seed longevity should focus on the fine mapping of QTLs with larger effects, such as *qLG-9*, *qLG-2*, and *qLG-4* [35].

## Materials and Methods

### Plasmid construction

Standard molecular biology procedures and DNA manipulations were performed to generate all constructs. Details of vector construction are available in the Supplemental Materials and Methods (S2 File). Sequences of the primers used are provided in (S2 Table). TALENs targeting endogenous genes were constructed through Golden Gate assembly. The plasmids for TALEN assembly were obtained from www.Addgene.org. The LIC method was modified to allow the incorporation of DNA fragments (i.e., TALEN cassettes) into the destination vectors [36]. Briefly, a reaction mixture was prepared on ice in a 20 μl volume containing a DNA fragment (400–500 ng), 1×NEB2 buffer (NEB), 0.1 mg/ml BSA (NEB), 1U T4 DNA polymerase (NEB) and 1 mM extension nucleotide triphosphate (NEB). Reactions were incubated at 25°C for 5 min and put on ice. When the PCR block reached the inactivation temperature of 75°C, 10 μl of the reaction mixture containing different DNA fragments was pooled in one tube for the annealing reaction. Then, the mixture was returned to the block at 75°C and incubated for 20 min, then slowly cooled to 55°C at a ramp rate of 1°C/min. After incubation for 20 min at 55°C, the samples were slowly cooled to room temperature at a rate of 0.4°C/min. The annealed mixture was used directly for bacterial cell transformation.

All the detail sequence of these vectors presented in (S1 File), and these vectors can be used in a wide range of functional genomics projects in plants and will be distributed to the research community for noncommercial research purposes upon request.

### Transient reporter assay in yeast and rice protoplasts

The yeast assay for testing TALEN pair activity was carried out as described previously [23]. GFP-positive cells were counted by flow cytometry (BD FACS ARIA II SORP) after 16 h of incubation according to the manufacturer’s instructions. At least 30,000 cells were analyzed for each sample.

Rice protoplasts were isolated and transformed as previously described [37]. Protoplasts were collected by centrifuging at 13,000 rpm for 2 min, and the supernatants were discarded. DNA was extracted using the DNAsecure Plant Kit (Tiangen, DP320), with final concentrations of ~30 ng/μL in 50-μL elution volumes. Protoplast DNA (~400 ng) was digested by *Nae*I in a 20 μL volume, then 8 μL of the digested product was used as the template in a 40 μL PCR reaction. Target sites were amplified, and PCR products were recovered using a DNA Gel Extraction Kit (Axygen). The PCR products (400 ng) were digested with *Nae*I, and undigested bands were detected by electrophoresis. Undigested PCR products and completely digested wild-type samples served as controls. The undigested product was cloned into a pMD18-T vector (Takara) to identify the mutation.

### Rice transformation and screening for the LOX3 knockout mutant

The rice variety ‘Nipponbare’ was used throughout these studies. *Agrobacterium*-mediated transformations of embryogenic calli were performed by Beijing Weiming Kaituo Co., Ltd.

Genomic DNA was extracted from T0 transgenic rice plants using the CTAB method, and PCR reactions were performed to amplify sequences across the LOX3-TALEN recognition site. In total, 400 ng of PCR product was digested by NaeI (NEB) and was separated via 1% agarose gel electrophoresis. The undigested PCR products and fully digested wild-type samples served as controls. The NaeI-insensitive fragments were cloned for sequencing to determine the mutation type.

### Western blotting and KI-I2 starch assay

The T3 generation of Nipponbare seeds (mutant and wild type) and Daw Dam (LOX3-null variety) were used in these experiments. Total protein was extracted from seeds using a protein extraction buffer (10 mM KCl, 1 mM EDTA at pH 8.0, 1 mM EGTA, 1 mM MgCl2, 400 mM sugar, 100 mM potassium phosphate, and 1% AEBSF). Total protein (30 μg) was loaded, and western blotting analysis was performed using monoclonal mouse anti-LOX3 (Abmart) [16] and anti-HSP82 (BPI) antibodies. Horseradish peroxidase activities were measured using the EasySee Western Blot Kit (TransGen).

The KI-I2 starch assay is a simple and convenient method for detecting LOX3 deficiency in rice seeds by monitoring color change [11]. Twelve rice seed embryos were put into a 1.5-mL centrifuge tube and crushed using a pestle, then 0.5 mL of 0.2 M borate/boric acid buffer solution (1 L contains 6.67 g Borax and 8.03 g borax acid, pH 8.2) was added to the tube and shaken vigorously. The tube was incubated at room temperature for 1 h, then 0.5 mL of 0.16 mM linoleic acid (diluted in the borate/boric acid buffer) was added, mixed by inversion, and incubated for 10 min. Then, 100 μL KI solution (saturated KI solution: 15% glacial acetic acid, 5:95) and 100 μL 1% starch (dissolved by borate/boric acid buffer) were added, the tube was placed in the dark for 12 h, and the final color of the reaction was recorded. Purple or red indicated that the embryos expressed functional LOX3, while a lack of color suggested a lack of LOX3 activity in the embryos.

### Evaluation of rice seed longevity

Artificial aging was performed to evaluate seed longevity based on the germination rate after several days of storage. Harvested T3 generation Nipponbare (TA-lox3 and TA-wt) and Daw Dam (LOX3-null variety) seeds were placed in a drying oven for 3 days at 45°C to reduce their water content. Then, the seeds were placed in a chamber (Sanyo, MLR-351H) at 42°C with 88% relative humidity for 0–15 days. The seeds were removed for germination. A total of 100 plump seeds from each individual were placed on three layers of filter paper in a petri dish, then enough water was added to immerse the seeds fully, and the dishes were incubated at 37°C for 2 days. After, the water was replaced, and the dishes were placed in a growth chamber at 30°C with a 12-h light/12-h dark cycle. Water was added as needed. The germination rates were scored after 7 days.

### RNA isolation and quantitative RT-PCR analysis

Total RNA was isolated from 250 mg seeds of TA-lox3 and TA-wt seeds before and after 10 days aging treatment using TRIzol reagent[16], First-strand cDNA was synthesized from 2 μg of total RNA using a cDNA reverse transcription kit (TIANGEN, KR1601,CHINA) following the manufacturer’s instructions. Real-time quantitative PCR was performed using the Bio-Rad CFX-96 system and a SYBR Green Ex-Taq Premix (TAKARA,RR420A, JAPAN). For the quantitative RT-PCR analysis, LOX1 and LOX2 specific primers were used and EF-1α was used as reference gene. The primer used for quantitative RT-PCR as follows: LOX1 (F-5’-CCAAGGCTTATGTTGCTGTTA-3’andR-5’-CCGCCGTTGATGAGTGT-3’), LOX2(F-5’-GGAGAAGAAGACGCGGTTG-3’andR-5’- ATACCCGGCGCCATCAT C-3’)and EF-1α (F-5’-CCTGTGGAAGTTCGAGACCA-3’and R-5’-CTGGCCAT CCTTGGAGATAC -3’).

### Determination of the fatty acid content

The aging seeds were first dehulled and frozen in liquid nitrogen, then ground into powder using a high-speed disintegrator (TL2010 made by DHSbio Ltd company, Beijing). Fatty acids content was analyzed according to the method described by Yin et al. [38]. Rice powder (0.3 g) was weighed into a vial, and heptadecanoic acid (17:0) was used as an internal standard. The boron trifluoride (BF_3_) method was used to prepare the methyl esters of fatty acids; then the material was analyzed by a Hewlett-Packard (HP) Model 6890 gas chromatography (GC) system (Wilmington, DE) coupled with a capillary column (CP-Sil 88 column, 100 m×250 μm, 0.25 μm film thickness, Chrompack, Palo Alto, CA). By comparing the retention times of rice samples with the corresponding standards, specific peaks were identified and quantified.

## Supporting Information

S1 Fig The segregation of the TALEN transgene in T1 mutants. Gel images of PCR products obtained with the primer sets for *FOKI*, *NPTII*, *TALE* and LOX3.
Lane 1~5 are representatives of T1 lines lack the T-DNA region; Lane6~8 are lines with T-DNA region.
(DOCX)

S1 File Sequence information for donor vectors pMaL01-p35S-TALEN, pMaL02-p35S-TALEN, pMaL01-2×p35S-GoldyTALEN, pMaL02-2×p35S-GoldyTALEN, pMaL01-pUbi-GoldyTALEN, pMaL02-pUbi-GoldyTALEN and pTALEN-DES01, pTALEN-DES02, pTALEN-DES03 (DOCX)

S2 File Supplemental materials and methods Construction of donor vectors and GFP report plasmid (DOCX)

S1 Table Information of engineered TALEN for lipoxygenase *LOX3*
(DOCX)

S2 Table List of PCR primers for construction of vector system, identification of transformation. (DOCX)
