# Supplementary material for: TALEN-Based Mutagenesis of Lipoxygenase LOX3 Enhances the Storage Tolerance of Rice (Oryza sativa) Seeds
Source: PLoS One. 2015 Dec 7;10(12):e0143877. doi: 10.1371/journal.pone.0143877 (PMC4671593; doi:10.1371/journal.pone.0143877)
Supplement: S1 Fig — Gel images of PCR products obtained with the primer sets for FOKI, NPTII, TALE and LOX3. Lane 1~5 are representatives of T1 lines lack the T-DNA region; Lane6~8 are lines with T-DNA region. (DOCX) [file pone.0143877.s001.docx]

**S1 Fig.**

**
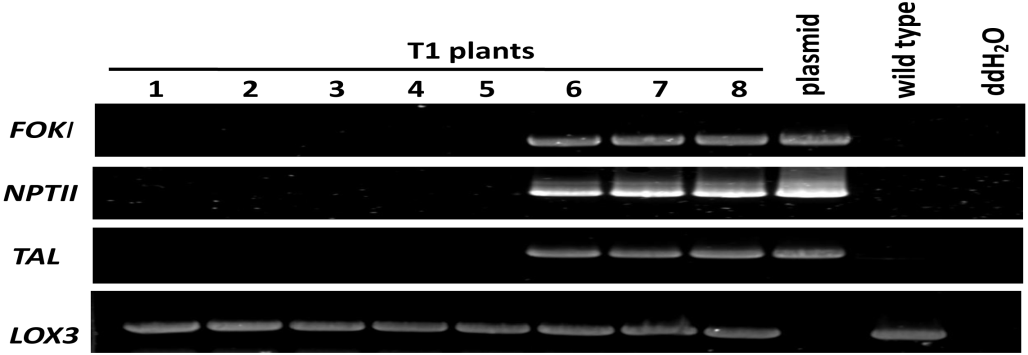
**

**S1 Fig. The segregation of the TALEN transgene in T1 mutants.**Gel images of PCR products obtained with the primer sets for *FOKI, NPTII, TALE* and LOX3.Lane 1~5 are representatives of T1 lines lack the T-DNA region; Lane6~8 are lines with T-DNA region.
