# Supplementary material for: TALEN-Based Mutagenesis of Lipoxygenase LOX3 Enhances the Storage Tolerance of Rice (Oryza sativa) Seeds
Source: PLoS One. 2015 Dec 7;10(12):e0143877. doi: 10.1371/journal.pone.0143877 (PMC4671593; doi:10.1371/journal.pone.0143877)
Supplement: S1 File — (DOCX) [file pone.0143877.s002.docx]

**Sequence information for vectors**

pMaL01-p35S-TALEN:

1 TCGCGCGTTT CGGTGATGAC GGTGAAAACC TCTGACACAT GCAGCTCCCG GTCACGGTCA

61 CAGCTTGTCT GTAAGCGGAT GCCGGGAGCA GACAAGCCCG TCAGGGCGCG TCAGCGGGTG

121 TTGGCGGGTG TCGGGGCTGG CTTAACTATG CGGCATCAGA GCAGATTGTA CTGAGAGTGC

181 ACCATATGCG GTGTGAAATA CCGCACAGAT GCGTAAGGAG AAAATACCGC ATCAGGCGCC

241 ATTCGCCATT CAGGCTGCGC AACTGTTGGG AAGGGCGATC GGTGCGGGCC TCTTCGCTAT

301 TACGCCAGCT GGCGAAAGGG GGATGTGCTG CAAGGCGATT AAGTTGGGTA ACGCCAGGGT

361 TTTCCCAGTC ACGACGTTGT AAAACGACGG CCAGTGCCAA AGAAGCATGA CGGCAAGTGG

421 ACGATTCTTA ATTAAGGGGG GTTACCGGGT ACCCATGGAG TCAAAGATTC AAATAGAGGA

481 CCTAACAGAA CTCGCCGTAA AGACTGGCGA ACAGTTCATA CAGAGTCTCT TACGACTCAA

541 TGACAAGAAG AAAATCTTCG TCAACATGGT GGAGCACGAC ACACTTGTCT ACTCCAAAAA

601 TATCAAAGAT ACAGTCTCAG AAGACCAAAG GGCAATTGAG ACTTTTCAAC AAAGGGTAAT

661 ATCCGGAAAC CTCCTCGGAT TCCATTGCCC AGCTATCTGT CACTTTATTG TGAAGATAGT

721 GGAAAAGGAA GGTGGCTCCT ACAAATGCCA TCATTGCGAT AAAGGAAAGG CCATCGTTGA

781 AGATGCCTCT GCCGACAGTG GTCCCAAAGA TGGACCCCCA CCCACGAGGA GCATCGTGGA

841 AAAAGAAGAC GTTCCAACCA CGTCTTCAAA GCAAGTGGAT TGATGTGATA TCTCCACTGA

901 CGTAAGGGAT GACGCACAAT CCCACTATCC TTCGCAAGAC CCTTCCTCTA TATAAGGAAG

961 TTCATTTCAT TTGGAGAGAA CACGGGGGAC TCTCGAGAAA GATATTGTAT ATATCGTAAC

1021 AATAGGAGGT TCAACAATGG CTTCCTCCCC TCCAAAGAAA AAGAGAAAGG TTAGTTGGAA

1081 GGACGCAAGT GGTTGGTCTA GAATGCATGC GGATCCCATT CGTCCGCGCA GGCCAAGTCC

1141 TGCCCGCGAG CTTCTGCCCG GACCCCAACC GGATAGGGTT CAGCCGACTG CAGATCGTGG

1201 GGTGTCTGCG CCTGCTGGCA GCCCTCTGGA TGGCTTGCCC GCTCGGCGGA CGGTGTCCCG

1261 GACCCGGCTG CCATCTCCCC CTGCGCCCTC ACCTGCGTTC TCGGCGGGCA GCTTCAGCGA

1321 TCTGCTCCGT CCGTTCGATC CGTCGCTTCT TGATACATCG CTTCTTGATT CGATGCCTGC

1381 CGTCGGCACG CCGCATACAG CGGCTGCCCC AGCAGAGTGG GATGAGGCGC AATCGGCTCT

1441 GCGTGCAGCC GATGACCCGC CACCCACCGT GCGTGTCGCT GTCACTGCCG CGCGGCCGCC

1501 GCGCGCCAAG CCGGCCCCGC GACGGCGTGC TGCGCAACCC TCCGACGCTT CGCCGGCCGC

1561 GCAGGTGGAT CTACGCACGC TCGGCTACAG TCAGCAGCAG CAAGAGAAGA TCAAACCGAA

1621 GGTGCGTTCG ACAGTGGCGC AGCACCACGA GGCACTGGTG GGCCATGGGT TTACACACGC

1681 GCACATCGTT GCGCTCAGCC AACACCCGGC AGCGTTAGGG ACCGTCGCTG TCACGTATCA

1741 GCACATAATC ACGGCGTTGC CAGAGGCGAC ACACGAAGAC ATCGTTGGCG TCGGCAAACA

1801 GTGGTCCGGC GCACGCGCCC TGGAGGCCTT GCTCACGGAT GCGGGGGAGT TGAGAGGTCC

1861 GCCGTTACAG TTGGACACAG GCCAACTTGT GAAGATTGCA AAACGTGGCG GCGTGACCGC

1921 AATGGAGGCA GTGCATGCAT CGCGCAATGC ACTGACGGGT GCCCCCCTGG AGACGATGGT

1981 GAGCAAGGGC GAGGAGCTGT TCACCGGGGT GGTGCCCATC CTGGTCGAGC TGGACGGCGA

2041 CGTAAACGGC CACAAGTTCA GCGTGTCCGG CGAGGGCGAG GGCGATGCCA CCTACGGCAA

2101 GCTGACCCTG AAGTTCATCT GCACCACCGG CAAGCTGCCC GTGCCCTGGC CCACCCTCGT

2161 GACCACCCTG ACCTACGGCG TGCAGTGCTT CAGCCGCTAC CCCGACCACA TGAAGCAGCA

2221 CGACTTCTTC AAGTCCGCCA TGCCCGAAGG CTACGTCCAG GAGCGCACCA TCTTCTTCAA

2281 GGACGACGGC AACTACAAGA CCCGCGCCGA GGTGAAGTTC GAGGGCGACA CCCTGGTGAA

2341 CCGCATCGAG CTGAAGGGCA TCGACTTCAA GGAGGACGGC AACATCCTGG GGCACAAGCT

2401 GGAGTACAAC TACAACAGCC ACAACGTCTA TATCATGGCC GACAAGCAGA AGAACGGCAT

2461 CAAGGTGAAC TTCAAGATCC GCCACAACAT CGAGGACGGC AGCGTGCAGC TCGCCGACCA

2521 CTACCAGCAG AACACCCCCA TCGGCGACGG CCCCGTGCTG CTGCCCGACA ACCACTACCT

2581 GAGCACCCAG TCCGCCCTGA GCAAAGACCC CAACGAGAAG CGCGATCACA TGGTCCTGCT

2641 GGAGTTCGTG ACCGCCGCCG GGATCACTCT CGGCATGGAC GAGCTGTACA AGTAACGTCT

2701 CCAACGACCA CCTCGTCGCC TTGGCCTGCC TCGGCGGACG TCCTGCCATG GATGCAGTGA

2761 AAAAGGGATT GCCGCACGCG CCGGAATTGA TCAGAAGAGT CAATCGCCGT ATTGGCGAAC

2821 GCACGTCCCA TCGCGTTGCC GACTACGCGC AAGTGGTTCG CGTGCTGGAG TTTTTCCAGT

2881 GCCACTCCCA CCCAGCGTAC GCATTTGATG AGGCCATGAC GCAGTTCGGG ATGAGCAGGA

2941 ACGGGTTGGT ACAGCTCTTT CGCAGAGTGG GCGTCACCGA ACTCGAAGCC CGCGGTGGAA

3001 CGCTCCCCCC AGCCTCGCAG CGTTGGGACC GTATCCTCCA GGCATCAGGG ATGAAAAGGG

3061 CCAAACCGTC CCCTACTTCA GCTCAAACAC CGGATCAGGC GTCTTTGCAT GCATTCGCCG

3121 ATTCGCTGGA GCGTGACCTT GATGCGCCCA GCCCAATGCA CGAGGGAGAT CAGACGCGGG

3181 CAAGCAGCCG TAAACGGTCC CGATCGGATC GTGCTGTCAC CGGCCCCTCC GCACAGCAGG

3241 CTGTCGAGGT GCGCGTTCCC GAACAGCGCG ATGCGCTGCA TTTGCCCCTC AGCTGGAGGG

3301 TAAAACGCCC GCGTACCAGG ATCTGGGGCG GCCTCCCGGA TCCGATATCT AGATCCCAGC

3361 TAGTGAAATC TGAATTGGAA GAGAAGAAAT CTGAACTTAG ACATAAATTG AAATATGTGC

3421 CACATGAATA TATTGAATTG ATTGAAATCG CAAGAAATTC AACTCAGGAT AGAATCCTTG

3481 AAATGAAGGT GATGGAGTTC TTTATGAAGG TTTATGGTTA TCGTGGTAAA CATTTGGGTG

3541 GATCAAGGAA ACCAGACGGA GCAATTTATA CTGTCGGATC TCCTATTGAT TACGGTGTGA

3601 TCGTTGATAC TAAGGCATAT TCAGGAGGTT ATAATCTTCC AATTGGTCAA GCAGATGAAA

3661 TGCAAAGATA TGTCAAAGAG AATCAAACAA GAAACAAGCA TATCAACCCT AATGAATGGT

3721 GGAAAGTCTA TCCATCTTCA GTAACAGAAT TTAAGTTCTT GTTTGTGAGT GGTCATTTCA

3781 AAGGAAACTA CAAAGCTCAG CTTACAAGAT TGAATCATAA GACTAATTGT AATGGAGCTG

3841 TTCTTAGTGT AGAAGAGCTT TTGATTGGTG GAGAAATGAT TAAAGCTGGT ACATTGACAC

3901 TTGAGGAAGT GAGAAGGAAA TTTAATAACG GTGAGATAAA CTTTTAATAG GAGCTCGGAT

3961 CGTTCAAACA TTTGGCAATA AAGTTTCTTA AGATTGAATC CTGTTGCCGG TCTTGCGATG

4021 ATTATCATAT AATTTCTGTT GAATTACGTT AAGCATGTAA TAATTAACAT GTAATGCATG

4081 ACGTTATTTA TGAGATGGGT TTTTATGATT AGAGTCCCGC AATTATACAT TTAATACGCG

4141 ATAGAAAACA AAATATAGCG CGCAAACTAG GATAAATTAT CGCGCGCGGT GTCATCTATG

4201 TTACTAGATC GGGCGCGCCT TGCGCGTCGG TAAATCATCT TAATTAAGAA TCTCCAGAGG

4261 ATCGCCGGGA ACCGAGGACG AGTTCGTAAT CATGGTCATA GCTGTTTCCT GTGTGAAATT

4321 GTTATCCGCT CACAATTCCA CACAACATAC GAGCCGGAAG CATAAAGTGT AAAGCCTGGG

4381 GTGCCTAATG AGTGAGCTAA CTCACATTAA TTGCGTTGCG CTCACTGCCC GCTTTCCAGT

4441 CGGGAAACCT GTCGTGCCAG CTGCATTAAT GAATCGGCCA ACGCGCGGGG AGAGGCGGTT

4501 TGCGTATTGG GCGCTCTTCC GCTTCCTCGC TCACTGACTC GCTGCGCTCG GTCGTTCGGC

4561 TGCGGCGAGC GGTATCAGCT CACTCAAAGG CGGTAATACG GTTATCCACA GAATCAGGGG

4621 ATAACGCAGG AAAGAACATG TGAGCAAAAG GCCAGCAAAA GGCCAGGAAC CGTAAAAAGG

4681 CCGCGTTGCT GGCGTTTTTC CATAGGCTCC GCCCCCCTGA CGAGCATCAC AAAAATCGAC

4741 GCTCAAGTCA GAGGTGGCGA AACCCGACAG GACTATAAAG ATACCAGGCG TTTCCCCCTG

4801 GAAGCTCCCT CGTGCGCTCT CCTGTTCCGA CCCTGCCGCT TACCGGATAC CTGTCCGCCT

4861 TTCTCCCTTC GGGAAGCGTG GCGCTTTCTC ATAGCTCACG CTGTAGGTAT CTCAGTTCGG

4921 TGTAGGTCGT TCGCTCCAAG CTGGGCTGTG TGCACGAACC CCCCGTTCAG CCCGACCGCT

4981 GCGCCTTATC CGGTAACTAT CGTCTTGAGT CCAACCCGGT AAGACACGAC TTATCGCCAC

5041 TGGCAGCAGC CACTGGTAAC AGGATTAGCA GAGCGAGGTA TGTAGGCGGT GCTACAGAGT

5101 TCTTGAAGTG GTGGCCTAAC TACGGCTACA CTAGAAGAAC AGTATTTGGT ATCTGCGCTC

5161 TGCTGAAGCC AGTTACCTTC GGAAAAAGAG TTGGTAGCTC TTGATCCGGC AAACAAACCA

5221 CCGCTGGTAG CGGTGGTTTT TTTGTTTGCA AGCAGCAGAT TACGCGCAGA AAAAAAGGAT

5281 CTCAAGAAGA TCCTTTGATC TTTTCTACGG GGTCTGACGC TCAGTGGAAC GAAAACTCAC

5341 GTTAAGGGAT TTTGGTCATG AGATTATCAA AAAGGATCTT CACCTAGATC CTTTTAAATT

5401 AAAAATGAAG TTTTAAATCA ATCTAAAGTA TATATGAGTA AACTTGGTCT GACAGTTACC

5461 AATGCTTAAT CAGTGAGGCA CCTATCTCAG CGATCTGTCT ATTTCGTTCA TCCATAGTTG

5521 CCTGACTCCC CGTCGTGTAG ATAACTACGA TACGGGAGGG CTTACCATCT GGCCCCAGTG

5581 CTGCAATGAT ACCGCGAGAC CCACGCTCAC CGGCTCCAGA TTTATCAGCA ATAAACCAGC

5641 CAGCCGGAAG GGCCGAGCGC AGAAGTGGTC CTGCAACTTT ATCCGCCTCC ATCCAGTCTA

5701 TTAATTGTTG CCGGGAAGCT AGAGTAAGTA GTTCGCCAGT TAATAGTTTG CGCAACGTTG

5761 TTGCCATTGC TACAGGCATC GTGGTGTCAC GCTCGTCGTT TGGTATGGCT TCATTCAGCT

5821 CCGGTTCCCA ACGATCAAGG CGAGTTACAT GATCCCCCAT GTTGTGCAAA AAAGCGGTTA

5881 GCTCCTTCGG TCCTCCGATC GTTGTCAGAA GTAAGTTGGC CGCAGTGTTA TCACTCATGG

5941 TTATGGCAGC ACTGCATAAT TCTCTTACTG TCATGCCATC CGTAAGATGC TTTTCTGTGA

6001 CTGGTGAGTA CTCAACCAAG TCATTCTGAG AATAGTGTAT GCGGCGACCG AGTTGCTCTT

6061 GCCCGGCGTC AATACGGGAT AATACCGCGC CACATAGCAG AACTTTAAAA GTGCTCATCA

6121 TTGGAAAACG TTCTTCGGGG CGAAAACTCT CAAGGATCTT ACCGCTGTTG AGATCCAGTT

6181 CGATGTAACC CACTCGTGCA CCCAACTGAT CTTCAGCATC TTTTACTTTC ACCAGCGTTT

6241 CTGGGTGAGC AAAAACAGGA AGGCAAAATG CCGCAAAAAA GGGAATAAGG GCGACACGGA

6301 AATGTTGAAT ACTCATACTC TTCCTTTTTC AATATTATTG AAGCATTTAT CAGGGTTATT

6361 GTCTCATGAG CGGATACATA TTTGAATGTA TTTAGAAAAA TAAACAAATA GGGGTTCCGC

6421 GCACATTTCC CCGAAAAGTG CCACCTGACG TCTAAGAAAC CATTATTATC ATGACATTAA

6481 CCTATAAAAA TAGGCGTATC ACGAGGCCCT TTCGAG

pMaL02-p35S-TALEN:

1 TCGCGCGTTT CGGTGATGAC GGTGAAAACC TCTGACACAT GCAGCTCCCG GTCACGGTCA

61 CAGCTTGTCT GTAAGCGGAT GCCGGGAGCA GACAAGCCCG TCAGGGCGCG TCAGCGGGTG

121 TTGGCGGGTG TCGGGGCTGG CTTAACTATG CGGCATCAGA GCAGATTGTA CTGAGAGTGC

181 ACCATATGCG GTGTGAAATA CCGCACAGAT GCGTAAGGAG AAAATACCGC ATCAGGCGCC

241 ATTCGCCATT CAGGCTGCGC AACTGTTGGG AAGGGCGATC GGTGCGGGCC TCTTCGCTAT

301 TACGCCAGCT GGCGAAAGGG GGATGTGCTG CAAGGCGATT AAGTTGGGTA ACGCCAGGGT

361 TTTCCCAGTC ACGACGTTGT AAAACGACGG CCAGTGCCAA AGAAGCATGA CGGCAAGTGG

421 ACGATTCTTA ATTAAATCAT CTTAGGGGGT ACCCATGGAG TCAAAGATTC AAATAGAGGA

481 CCTAACAGAA CTCGCCGTAA AGACTGGCGA ACAGTTCATA CAGAGTCTCT TACGACTCAA

541 TGACAAGAAG AAAATCTTCG TCAACATGGT GGAGCACGAC ACACTTGTCT ACTCCAAAAA

601 TATCAAAGAT ACAGTCTCAG AAGACCAAAG GGCAATTGAG ACTTTTCAAC AAAGGGTAAT

661 ATCCGGAAAC CTCCTCGGAT TCCATTGCCC AGCTATCTGT CACTTTATTG TGAAGATAGT

721 GGAAAAGGAA GGTGGCTCCT ACAAATGCCA TCATTGCGAT AAAGGAAAGG CCATCGTTGA

781 AGATGCCTCT GCCGACAGTG GTCCCAAAGA TGGACCCCCA CCCACGAGGA GCATCGTGGA

841 AAAAGAAGAC GTTCCAACCA CGTCTTCAAA GCAAGTGGAT TGATGTGATA TCTCCACTGA

901 CGTAAGGGAT GACGCACAAT CCCACTATCC TTCGCAAGAC CCTTCCTCTA TATAAGGAAG

961 TTCATTTCAT TTGGAGAGAA CACGGGGGAC TCTCGAGAAA GATATTGTAT ATATCGTAAC

1021 AATAGGAGGT TCAACAATGG CTTCCTCCCC TCCAAAGAAA AAGAGAAAGG TTAGTTGGAA

1081 GGACGCAAGT GGTTGGTCTA GAATGCATGC GGATCCCATT CGTCCGCGCA GGCCAAGTCC

1141 TGCCCGCGAG CTTCTGCCCG GACCCCAACC GGATAGGGTT CAGCCGACTG CAGATCGTGG

1201 GGTGTCTGCG CCTGCTGGCA GCCCTCTGGA TGGCTTGCCC GCTCGGCGGA CGGTGTCCCG

1261 GACCCGGCTG CCATCTCCCC CTGCGCCCTC ACCTGCGTTC TCGGCGGGCA GCTTCAGCGA

1321 TCTGCTCCGT CCGTTCGATC CGTCGCTTCT TGATACATCG CTTCTTGATT CGATGCCTGC

1381 CGTCGGCACG CCGCATACAG CGGCTGCCCC AGCAGAGTGG GATGAGGCGC AATCGGCTCT

1441 GCGTGCAGCC GATGACCCGC CACCCACCGT GCGTGTCGCT GTCACTGCCG CGCGGCCGCC

1501 GCGCGCCAAG CCGGCCCCGC GACGGCGTGC TGCGCAACCC TCCGACGCTT CGCCGGCCGC

1561 GCAGGTGGAT CTACGCACGC TCGGCTACAG TCAGCAGCAG CAAGAGAAGA TCAAACCGAA

1621 GGTGCGTTCG ACAGTGGCGC AGCACCACGA GGCACTGGTG GGCCATGGGT TTACACACGC

1681 GCACATCGTT GCGCTCAGCC AACACCCGGC AGCGTTAGGG ACCGTCGCTG TCACGTATCA

1741 GCACATAATC ACGGCGTTGC CAGAGGCGAC ACACGAAGAC ATCGTTGGCG TCGGCAAACA

1801 GTGGTCCGGC GCACGCGCCC TGGAGGCCTT GCTCACGGAT GCGGGGGAGT TGAGAGGTCC

1861 GCCGTTACAG TTGGACACAG GCCAACTTGT GAAGATTGCA AAACGTGGCG GCGTGACCGC

1921 AATGGAGGCA GTGCATGCAT CGCGCAATGC ACTGACGGGT GCCCCCCTGG AGACGATGGT

1981 GAGCAAGGGC GAGGAGCTGT TCACCGGGGT GGTGCCCATC CTGGTCGAGC TGGACGGCGA

2041 CGTAAACGGC CACAAGTTCA GCGTGTCCGG CGAGGGCGAG GGCGATGCCA CCTACGGCAA

2101 GCTGACCCTG AAGTTCATCT GCACCACCGG CAAGCTGCCC GTGCCCTGGC CCACCCTCGT

2161 GACCACCCTG ACCTACGGCG TGCAGTGCTT CAGCCGCTAC CCCGACCACA TGAAGCAGCA

2221 CGACTTCTTC AAGTCCGCCA TGCCCGAAGG CTACGTCCAG GAGCGCACCA TCTTCTTCAA

2281 GGACGACGGC AACTACAAGA CCCGCGCCGA GGTGAAGTTC GAGGGCGACA CCCTGGTGAA

2341 CCGCATCGAG CTGAAGGGCA TCGACTTCAA GGAGGACGGC AACATCCTGG GGCACAAGCT

2401 GGAGTACAAC TACAACAGCC ACAACGTCTA TATCATGGCC GACAAGCAGA AGAACGGCAT

2461 CAAGGTGAAC TTCAAGATCC GCCACAACAT CGAGGACGGC AGCGTGCAGC TCGCCGACCA

2521 CTACCAGCAG AACACCCCCA TCGGCGACGG CCCCGTGCTG CTGCCCGACA ACCACTACCT

2581 GAGCACCCAG TCCGCCCTGA GCAAAGACCC CAACGAGAAG CGCGATCACA TGGTCCTGCT

2641 GGAGTTCGTG ACCGCCGCCG GGATCACTCT CGGCATGGAC GAGCTGTACA AGTAACGTCT

2701 CCAACGACCA CCTCGTCGCC TTGGCCTGCC TCGGCGGACG TCCTGCCATG GATGCAGTGA

2761 AAAAGGGATT GCCGCACGCG CCGGAATTGA TCAGAAGAGT CAATCGCCGT ATTGGCGAAC

2821 GCACGTCCCA TCGCGTTGCC GACTACGCGC AAGTGGTTCG CGTGCTGGAG TTTTTCCAGT

2881 GCCACTCCCA CCCAGCGTAC GCATTTGATG AGGCCATGAC GCAGTTCGGG ATGAGCAGGA

2941 ACGGGTTGGT ACAGCTCTTT CGCAGAGTGG GCGTCACCGA ACTCGAAGCC CGCGGTGGAA

3001 CGCTCCCCCC AGCCTCGCAG CGTTGGGACC GTATCCTCCA GGCATCAGGG ATGAAAAGGG

3061 CCAAACCGTC CCCTACTTCA GCTCAAACAC CGGATCAGGC GTCTTTGCAT GCATTCGCCG

3121 ATTCGCTGGA GCGTGACCTT GATGCGCCCA GCCCAATGCA CGAGGGAGAT CAGACGCGGG

3181 CAAGCAGCCG TAAACGGTCC CGATCGGATC GTGCTGTCAC CGGCCCCTCC GCACAGCAGG

3241 CTGTCGAGGT GCGCGTTCCC GAACAGCGCG ATGCGCTGCA TTTGCCCCTC AGCTGGAGGG

3301 TAAAACGCCC GCGTACCAGG ATCTGGGGCG GCCTCCCGGA TCCGATATCT AGATCCCAGC

3361 TAGTGAAATC TGAATTGGAA GAGAAGAAAT CTGAACTTAG ACATAAATTG AAATATGTGC

3421 CACATGAATA TATTGAATTG ATTGAAATCG CAAGAAATTC AACTCAGGAT AGAATCCTTG

3481 AAATGAAGGT GATGGAGTTC TTTATGAAGG TTTATGGTTA TCGTGGTAAA CATTTGGGTG

3541 GATCAAGGAA ACCAGACGGA GCAATTTATA CTGTCGGATC TCCTATTGAT TACGGTGTGA

3601 TCGTTGATAC TAAGGCATAT TCAGGAGGTT ATAATCTTCC AATTGGTCAA GCAGATGAAA

3661 TGGAAAGATA TGTCGAAGAG AATCAAACAA GAAACAAGCA TCTCAACCCT AATGAATGGT

3721 GGAAAGTCTA TCCATCTTCA GTAACAGAAT TTAAGTTCTT GTTTGTGAGT GGTCATTTCA

3781 AAGGAAACTA CAAAGCTCAG CTTACAAGAT TGAATCATAT CACTAATTGT AATGGAGCTG

3841 TTCTTAGTGT AGAAGAGCTT TTGATTGGTG GAGAAATGAT TAAAGCTGGT ACATTGACAC

3901 TTGAGGAAGT GAGAAGGAAA TTTAATAACG GTGAGATAAA CTTTTAATAG GAGCTCGGAT

3961 CGTTCAAACA TTTGGCAATA AAGTTTCTTA AGATTGAATC CTGTTGCCGG TCTTGCGATG

4021 ATTATCATAT AATTTCTGTT GAATTACGTT AAGCATGTAA TAATTAACAT GTAATGCATG

4081 ACGTTATTTA TGAGATGGGT TTTTATGATT AGAGTCCCGC AATTATACAT TTAATACGCG

4141 ATAGAAAACA AAATATAGCG CGCAAACTAG GATAAATTAT CGCGCGCGGT GTCATCTATG

4201 TTACTAGATC GGGCGCGCCT TACGCGTCCC TAATAATAAT TAATTAAGAA TCTCCAGAGG

4261 ATCGCCGGGA ACCGAGGACG AGTTCGTAAT CATGGTCATA GCTGTTTCCT GTGTGAAATT

4321 GTTATCCGCT CACAATTCCA CACAACATAC GAGCCGGAAG CATAAAGTGT AAAGCCTGGG

4381 GTGCCTAATG AGTGAGCTAA CTCACATTAA TTGCGTTGCG CTCACTGCCC GCTTTCCAGT

4441 CGGGAAACCT GTCGTGCCAG CTGCATTAAT GAATCGGCCA ACGCGCGGGG AGAGGCGGTT

4501 TGCGTATTGG GCGCTCTTCC GCTTCCTCGC TCACTGACTC GCTGCGCTCG GTCGTTCGGC

4561 TGCGGCGAGC GGTATCAGCT CACTCAAAGG CGGTAATACG GTTATCCACA GAATCAGGGG

4621 ATAACGCAGG AAAGAACATG TGAGCAAAAG GCCAGCAAAA GGCCAGGAAC CGTAAAAAGG

4681 CCGCGTTGCT GGCGTTTTTC CATAGGCTCC GCCCCCCTGA CGAGCATCAC AAAAATCGAC

4741 GCTCAAGTCA GAGGTGGCGA AACCCGACAG GACTATAAAG ATACCAGGCG TTTCCCCCTG

4801 GAAGCTCCCT CGTGCGCTCT CCTGTTCCGA CCCTGCCGCT TACCGGATAC CTGTCCGCCT

4861 TTCTCCCTTC GGGAAGCGTG GCGCTTTCTC ATAGCTCACG CTGTAGGTAT CTCAGTTCGG

4921 TGTAGGTCGT TCGCTCCAAG CTGGGCTGTG TGCACGAACC CCCCGTTCAG CCCGACCGCT

4981 GCGCCTTATC CGGTAACTAT CGTCTTGAGT CCAACCCGGT AAGACACGAC TTATCGCCAC

5041 TGGCAGCAGC CACTGGTAAC AGGATTAGCA GAGCGAGGTA TGTAGGCGGT GCTACAGAGT

5101 TCTTGAAGTG GTGGCCTAAC TACGGCTACA CTAGAAGAAC AGTATTTGGT ATCTGCGCTC

5161 TGCTGAAGCC AGTTACCTTC GGAAAAAGAG TTGGTAGCTC TTGATCCGGC AAACAAACCA

5221 CCGCTGGTAG CGGTGGTTTT TTTGTTTGCA AGCAGCAGAT TACGCGCAGA AAAAAAGGAT

5281 CTCAAGAAGA TCCTTTGATC TTTTCTACGG GGTCTGACGC TCAGTGGAAC GAAAACTCAC

5341 GTTAAGGGAT TTTGGTCATG AGATTATCAA AAAGGATCTT CACCTAGATC CTTTTAAATT

5401 AAAAATGAAG TTTTAAATCA ATCTAAAGTA TATATGAGTA AACTTGGTCT GACAGTTACC

5461 AATGCTTAAT CAGTGAGGCA CCTATCTCAG CGATCTGTCT ATTTCGTTCA TCCATAGTTG

5521 CCTGACTCCC CGTCGTGTAG ATAACTACGA TACGGGAGGG CTTACCATCT GGCCCCAGTG

5581 CTGCAATGAT ACCGCGAGAC CCACGCTCAC CGGCTCCAGA TTTATCAGCA ATAAACCAGC

5641 CAGCCGGAAG GGCCGAGCGC AGAAGTGGTC CTGCAACTTT ATCCGCCTCC ATCCAGTCTA

5701 TTAATTGTTG CCGGGAAGCT AGAGTAAGTA GTTCGCCAGT TAATAGTTTG CGCAACGTTG

5761 TTGCCATTGC TACAGGCATC GTGGTGTCAC GCTCGTCGTT TGGTATGGCT TCATTCAGCT

5821 CCGGTTCCCA ACGATCAAGG CGAGTTACAT GATCCCCCAT GTTGTGCAAA AAAGCGGTTA

5881 GCTCCTTCGG TCCTCCGATC GTTGTCAGAA GTAAGTTGGC CGCAGTGTTA TCACTCATGG

5941 TTATGGCAGC ACTGCATAAT TCTCTTACTG TCATGCCATC CGTAAGATGC TTTTCTGTGA

6001 CTGGTGAGTA CTCAACCAAG TCATTCTGAG AATAGTGTAT GCGGCGACCG AGTTGCTCTT

6061 GCCCGGCGTC AATACGGGAT AATACCGCGC CACATAGCAG AACTTTAAAA GTGCTCATCA

6121 TTGGAAAACG TTCTTCGGGG CGAAAACTCT CAAGGATCTT ACCGCTGTTG AGATCCAGTT

6181 CGATGTAACC CACTCGTGCA CCCAACTGAT CTTCAGCATC TTTTACTTTC ACCAGCGTTT

6241 CTGGGTGAGC AAAAACAGGA AGGCAAAATG CCGCAAAAAA GGGAATAAGG GCGACACGGA

6301 AATGTTGAAT ACTCATACTC TTCCTTTTTC AATATTATTG AAGCATTTAT CAGGGTTATT

6361 GTCTCATGAG CGGATACATA TTTGAATGTA TTTAGAAAAA TAAACAAATA GGGGTTCCGC

6421 GCACATTTCC CCGAAAAGTG CCACCTGACG TCTAAGAAAC CATTATTATC ATGACATTAA

6481 CCTATAAAAA TAGGCGTATC ACGAGGCCCT TTCGAG

pMaL01-2×p35S-GoldyTALEN:

1 TCGCGCGTTT CGGTGATGAC GGTGAAAACC TCTGACACAT GCAGCTCCCG GTCACGGTCA

61 CAGCTTGTCT GTAAGCGGAT GCCGGGAGCA GACAAGCCCG TCAGGGCGCG TCAGCGGGTG

121 TTGGCGGGTG TCGGGGCTGG CTTAACTATG CGGCATCAGA GCAGATTGTA CTGAGAGTGC

181 ACCATATGCG GTGTGAAATA CCGCACAGAT GCGTAAGGAG AAAATACCGC ATCAGGCGCC

241 ATTCGCCATT CAGGCTGCGC AACTGTTGGG AAGGGCGATC GGTGCGGGCC TCTTCGCTAT

301 TACGCCAGCT GGCGAAAGGG GGATGTGCTG CAAGGCGATT AAGTTGGGTA ACGCCAGGGT

361 TTTCCCAGTC ACGACGTTGT AAAACGACGG CCAGTGCCAA AGAAGCATGA CGGCAAGTGG

421 ACGATTCTTA ATTAAGGGGG GTTACCGGGT ACCGCCTCCT GTCAATGCTG GCGGCGGCTC

481 TGGTGGTGGT TCTGGTGGCG GCTCTGAGGG TGGTGGCTCT GAGGGTGGCG GTTCTGAGGG

541 TGGCGGCTCT GAGGGAGGCG GTTCCGGTGG TGGCTCTGGT TCCGGTGATT TTGATTATGA

601 AAAGATGGCA AACGCTAATA AGGGGGCTAT GACCGAAAAT GCCGATGAAA ACGCGCTACA

661 GTCTGACGCT AAAGGCAAAC TTGATTCTGT CGCTACTGAT TACGGTGCTG CTATCGATGG

721 TTTCATTGGT GACGTTTCCG GCCTTGCTAA TGGTAATGGT GCTACTGGTG ATTTTGCTGG

781 CTCTAATTCC CAAATGGCTC AAGTCGGTGA CGGTGATAAT TCACCTTTAA TGAATAATTT

841 CCGTCAATAT TTACCTTCCC TCCCTCAATC GGTTGAATGT CGCCCTTTTG TCTTTGGCCC

901 AATACGCAAA CCGCCTCTCC CCGCGCGTTG GCCGATTCAT TAATGCAGCT GGCACGACAG

961 GTTTCCCGAC TGGAAAGCGG GCAGTGAGCG CAACGCAATT AATGTGAGTT AGCTCACTCA

1021 TTAGGCACCC CAGGCTTTAC ACTTTATGCT TCCGGCTCGT ATGTTGTGTG GAATTGTGAG

1081 CGGATAACAA TTTCACACAG GAAACAGCTA TGACCATGAT TACGCCAAGC TTGCATGCCT

1141 GCAGGTCCCC AGATTAGCCT TTTCAATTTC AGAAAGAATG CTAACCCACA GATGGTTAGA

1201 GAGGCTTACG CAGCAGGTCT CATCAAGACG ATCTACCCGA GCAATAATCT CCAGGAAATC

1261 AAATACCTTC CCAAGAAGGT TAAAGATGCA GTCAAAAGAT TCAGGACTAA CTGCATCAAG

1321 AACACAGAGA AAGATATATT TCTCAAGATC AGAAGTACTA TTCCAGTATG GACGATTCAA

1381 GGCTTGCTTC ACAAACCAAG GCAAGTAATA GAGATTGGAG TCTCTAAAAA GGTAGTTCCC

1441 ACTGAATCAA AGGCCATGGA GTCAAAGATT CAAATAGAGG ACCTAACAGA ACTCGCCGTA

1501 AAGACTGGCG AACAGTTCAT ACAGAGTCTC TTACGACTCA ATGACAAGAA GAAAATCTTC

1561 GTCAACATGG TGGAGCACGA CACACTTGTC TACTCCAAAA ATATCAAAGA TACAGTCTCA

1621 GAAGACCAAA GGGCAATTGA GACTTTTCAA CAAAGGGTAA TATCCGGAAA CCTCCTCGGA

1681 TTCCATTGCC CAGCTATCTG TCACTTTATT GTGAAGATAG TGGAAAAGGA AGGTGGCTCC

1741 TACAAATGCC ATCATTGCGA TAAAGGAAAG GCCATCGTTG AAGATGCCTC TGCCGACAGT

1801 GGTCCCAAAG ATGGACCCCC ACCCACGAGG AGCATCGTGG AAAAAGAAGA CGTTCCAACC

1861 ACGTCTTCAA AGCAAGTGGA TTGATGTGAT ATCTCCACTG ACGTAAGGGA TGACGCACAA

1921 TCCCACTATC CTTCGCAAGA CCCTTCCTCT ATATAAGGAA GTTCATTTCA TTTGGAGAGA

1981 ACACGCTCGA GAAAGATATT GTATATATCG TAACAATAGG AGGTTCAACA ATGGCTTCCT

2041 CCCCTCCAAA GAAAAAGAGA AAGGTTAGTT GGAAGGACGC AAGTGGTTGG TCGCGAGTGG

2101 ATCTACGCAC GCTCGGCTAC AGTCAGCAGC AGCAAGAGAA GATCAAACCG AAGGTGCGTT

2161 CGACAGTGGC GCAGCACCAC GAGGCACTGG TGGGCCATGG GTTTACACAC GCGCACATCG

2221 TTGCGCTCAG CCAACACCCG GCAGCGTTAG GGACCGTCGC TGTCACGTAT CAGCACATAA

2281 TCACGGCGTT GCCAGAGGCG ACACACGAAG ACATCGTTGG CGTCGGCAAA CAGTGGTCCG

2341 GCGCACGCGC CCTGGAGGCC TTGCTCACGG ATGCGGGGGA GTTGAGAGGT CCGCCGTTAC

2401 AGTTGGACAC AGGCCAACTT GTGAAGATTG CAAAACGTGG CGGCGTGACC GCAATGGAGG

2461 CAGTGCATGC ATCGCGCAAT GCACTGACGG GTGCCCCCCT GGAGACGATG GTGAGCAAGG

2521 GCGAGGAGCT GTTCACCGGG GTGGTGCCCA TCCTGGTCGA GCTGGACGGC GACGTAAACG

2581 GCCACAAGTT CAGCGTGTCC GGCGAGGGCG AGGGCGATGC CACCTACGGC AAGCTGACCC

2641 TGAAGTTCAT CTGCACCACC GGCAAGCTGC CCGTGCCCTG GCCCACCCTC GTGACCACCC

2701 TGACCTACGG CGTGCAGTGC TTCAGCCGCT ACCCCGACCA CATGAAGCAG CACGACTTCT

2761 TCAAGTCCGC CATGCCCGAA GGCTACGTCC AGGAGCGCAC CATCTTCTTC AAGGACGACG

2821 GCAACTACAA GACCCGCGCC GAGGTGAAGT TCGAGGGCGA CACCCTGGTG AACCGCATCG

2881 AGCTGAAGGG CATCGACTTC AAGGAGGACG GCAACATCCT GGGGCACAAG CTGGAGTACA

2941 ACTACAACAG CCACAACGTC TATATCATGG CCGACAAGCA GAAGAACGGC ATCAAGGTGA

3001 ACTTCAAGAT CCGCCACAAC ATCGAGGACG GCAGCGTGCA GCTCGCCGAC CACTACCAGC

3061 AGAACACCCC CATCGGCGAC GGCCCCGTGC TGCTGCCCGA CAACCACTAC CTGAGCACCC

3121 AGTCCGCCCT GAGCAAAGAC CCCAACGAGA AGCGCGATCA CATGGTCCTG CTGGAGTTCG

3181 TGACCGCCGC CGGGATCACT CTCGGCATGG ACGAGCTGTA CAAGTAACGT CTCCAACGAC

3241 CACCTCGTCG CCTTGGCCTG CCTCGGCGGA CGTCCTGCCA TGGATGCAGT GAAAAAGGGA

3301 TTGCCGCACG CGCCGGAATT GATCAGAAGA GTCAATCGCC GTATTGGCGA ACGCACGTCC

3361 CATCGCGTTG CCTCTAGATC CCAGCTAGTG AAATCTGAAT TGGAAGAGAA GAAATCTGAA

3421 CTTAGACATA AATTGAAATA TGTGCCACAT GAATATATTG AATTGATTGA AATCGCAAGA

3481 AATTCAACTC AGGATAGAAT CCTTGAAATG AAGGTGATGG AGTTCTTTAT GAAGGTTTAT

3541 GGTTATCGTG GTAAACATTT GGGTGGATCA AGGAAACCAG ACGGAGCAAT TTATACTGTC

3601 GGATCTCCTA TTGATTACGG TGTGATCGTT GATACTAAGG CATATTCAGG AGGTTATAAT

3661 CTTCCAATTG GTCAAGCAGA TGAAATGCAA AGATATGTCA AAGAGAATCA AACAAGAAAC

3721 AAGCATATCA ACCCTAATGA ATGGTGGAAA GTCTATCCAT CTTCAGTAAC AGAATTTAAG

3781 TTCTTGTTTG TGAGTGGTCA TTTCAAAGGA AACTACAAAG CTCAGCTTAC AAGATTGAAT

3841 CATAAGACTA ATTGTAATGG AGCTGTTCTT AGTGTAGAAG AGCTTTTGAT TGGTGGAGAA

3901 ATGATTAAAG CTGGTACATT GACACTTGAG GAAGTGAGAA GGAAATTTAA TAACGGTGAG

3961 ATAAACTTTT AATAGGAGCT CGGATCGTTC AAACATTTGG CAATAAAGTT TCTTAAGATT

4021 GAATCCTGTT GCCGGTCTTG CGATGATTAT CATATAATTT CTGTTGAATT ACGTTAAGCA

4081 TGTAATAATT AACATGTAAT GCATGACGTT ATTTATGAGA TGGGTTTTTA TGATTAGAGT

4141 CCCGCAATTA TACATTTAAT ACGCGATAGA AAACAAAATA TAGCGCGCAA ACTAGGATAA

4201 ATTATCGCGC GCGGTGTCAT CTATGTTACT AGATCGGGCG CGCCTTGCGC GTCGGTAAAT

4261 CATCTTAATT AAGAATCTCC AGAGGATCGC CGGGAACCGA GGACGAGTTC GTAATCATGG

4321 TCATAGCTGT TTCCTGTGTG AAATTGTTAT CCGCTCACAA TTCCACACAA CATACGAGCC

4381 GGAAGCATAA AGTGTAAAGC CTGGGGTGCC TAATGAGTGA GCTAACTCAC ATTAATTGCG

4441 TTGCGCTCAC TGCCCGCTTT CCAGTCGGGA AACCTGTCGT GCCAGCTGCA TTAATGAATC

4501 GGCCAACGCG CGGGGAGAGG CGGTTTGCGT ATTGGGCGCT CTTCCGCTTC CTCGCTCACT

4561 GACTCGCTGC GCTCGGTCGT TCGGCTGCGG CGAGCGGTAT CAGCTCACTC AAAGGCGGTA

4621 ATACGGTTAT CCACAGAATC AGGGGATAAC GCAGGAAAGA ACATGTGAGC AAAAGGCCAG

4681 CAAAAGGCCA GGAACCGTAA AAAGGCCGCG TTGCTGGCGT TTTTCCATAG GCTCCGCCCC

4741 CCTGACGAGC ATCACAAAAA TCGACGCTCA AGTCAGAGGT GGCGAAACCC GACAGGACTA

4801 TAAAGATACC AGGCGTTTCC CCCTGGAAGC TCCCTCGTGC GCTCTCCTGT TCCGACCCTG

4861 CCGCTTACCG GATACCTGTC CGCCTTTCTC CCTTCGGGAA GCGTGGCGCT TTCTCATAGC

4921 TCACGCTGTA GGTATCTCAG TTCGGTGTAG GTCGTTCGCT CCAAGCTGGG CTGTGTGCAC

4981 GAACCCCCCG TTCAGCCCGA CCGCTGCGCC TTATCCGGTA ACTATCGTCT TGAGTCCAAC

5041 CCGGTAAGAC ACGACTTATC GCCACTGGCA GCAGCCACTG GTAACAGGAT TAGCAGAGCG

5101 AGGTATGTAG GCGGTGCTAC AGAGTTCTTG AAGTGGTGGC CTAACTACGG CTACACTAGA

5161 AGAACAGTAT TTGGTATCTG CGCTCTGCTG AAGCCAGTTA CCTTCGGAAA AAGAGTTGGT

5221 AGCTCTTGAT CCGGCAAACA AACCACCGCT GGTAGCGGTG GTTTTTTTGT TTGCAAGCAG

5281 CAGATTACGC GCAGAAAAAA AGGATCTCAA GAAGATCCTT TGATCTTTTC TACGGGGTCT

5341 GACGCTCAGT GGAACGAAAA CTCACGTTAA GGGATTTTGG TCATGAGATT ATCAAAAAGG

5401 ATCTTCACCT AGATCCTTTT AAATTAAAAA TGAAGTTTTA AATCAATCTA AAGTATATAT

5461 GAGTAAACTT GGTCTGACAG TTACCAATGC TTAATCAGTG AGGCACCTAT CTCAGCGATC

5521 TGTCTATTTC GTTCATCCAT AGTTGCCTGA CTCCCCGTCG TGTAGATAAC TACGATACGG

5581 GAGGGCTTAC CATCTGGCCC CAGTGCTGCA ATGATACCGC GAGACCCACG CTCACCGGCT

5641 CCAGATTTAT CAGCAATAAA CCAGCCAGCC GGAAGGGCCG AGCGCAGAAG TGGTCCTGCA

5701 ACTTTATCCG CCTCCATCCA GTCTATTAAT TGTTGCCGGG AAGCTAGAGT AAGTAGTTCG

5761 CCAGTTAATA GTTTGCGCAA CGTTGTTGCC ATTGCTACAG GCATCGTGGT GTCACGCTCG

5821 TCGTTTGGTA TGGCTTCATT CAGCTCCGGT TCCCAACGAT CAAGGCGAGT TACATGATCC

5881 CCCATGTTGT GCAAAAAAGC GGTTAGCTCC TTCGGTCCTC CGATCGTTGT CAGAAGTAAG

5941 TTGGCCGCAG TGTTATCACT CATGGTTATG GCAGCACTGC ATAATTCTCT TACTGTCATG

6001 CCATCCGTAA GATGCTTTTC TGTGACTGGT GAGTACTCAA CCAAGTCATT CTGAGAATAG

6061 TGTATGCGGC GACCGAGTTG CTCTTGCCCG GCGTCAATAC GGGATAATAC CGCGCCACAT

6121 AGCAGAACTT TAAAAGTGCT CATCATTGGA AAACGTTCTT CGGGGCGAAA ACTCTCAAGG

6181 ATCTTACCGC TGTTGAGATC CAGTTCGATG TAACCCACTC GTGCACCCAA CTGATCTTCA

6241 GCATCTTTTA CTTTCACCAG CGTTTCTGGG TGAGCAAAAA CAGGAAGGCA AAATGCCGCA

6301 AAAAAGGGAA TAAGGGCGAC ACGGAAATGT TGAATACTCA TACTCTTCCT TTTTCAATAT

6361 TATTGAAGCA TTTATCAGGG TTATTGTCTC ATGAGCGGAT ACATATTTGA ATGTATTTAG

6421 AAAAATAAAC AAATAGGGGT TCCGCGCACA TTTCCCCGAA AAGTGCCACC TGACGTCTAA

6481 GAAACCATTA TTATCATGAC ATTAACCTAT AAAAATAGGC GTATCACGAG GCCCTTTCGA

6541 G

pMaL02-2×p35S-GoldyTALEN:

1 TCGCGCGTTT CGGTGATGAC GGTGAAAACC TCTGACACAT GCAGCTCCCG GTCACGGTCA

61 CAGCTTGTCT GTAAGCGGAT GCCGGGAGCA GACAAGCCCG TCAGGGCGCG TCAGCGGGTG

121 TTGGCGGGTG TCGGGGCTGG CTTAACTATG CGGCATCAGA GCAGATTGTA CTGAGAGTGC

181 ACCATATGCG GTGTGAAATA CCGCACAGAT GCGTAAGGAG AAAATACCGC ATCAGGCGCC

241 ATTCGCCATT CAGGCTGCGC AACTGTTGGG AAGGGCGATC GGTGCGGGCC TCTTCGCTAT

301 TACGCCAGCT GGCGAAAGGG GGATGTGCTG CAAGGCGATT AAGTTGGGTA ACGCCAGGGT

361 TTTCCCAGTC ACGACGTTGT AAAACGACGG CCAGTGCCAA AGAAGCATGA CGGCAAGTGG

421 ACGATTCTTA ATTAAATCAT CTTAGGGGGT ACCGCCTCCT GTCAATGCTG GCGGCGGCTC

481 TGGTGGTGGT TCTGGTGGCG GCTCTGAGGG TGGTGGCTCT GAGGGTGGCG GTTCTGAGGG

541 TGGCGGCTCT GAGGGAGGCG GTTCCGGTGG TGGCTCTGGT TCCGGTGATT TTGATTATGA

601 AAAGATGGCA AACGCTAATA AGGGGGCTAT GACCGAAAAT GCCGATGAAA ACGCGCTACA

661 GTCTGACGCT AAAGGCAAAC TTGATTCTGT CGCTACTGAT TACGGTGCTG CTATCGATGG

721 TTTCATTGGT GACGTTTCCG GCCTTGCTAA TGGTAATGGT GCTACTGGTG ATTTTGCTGG

781 CTCTAATTCC CAAATGGCTC AAGTCGGTGA CGGTGATAAT TCACCTTTAA TGAATAATTT

841 CCGTCAATAT TTACCTTCCC TCCCTCAATC GGTTGAATGT CGCCCTTTTG TCTTTGGCCC

901 AATACGCAAA CCGCCTCTCC CCGCGCGTTG GCCGATTCAT TAATGCAGCT GGCACGACAG

961 GTTTCCCGAC TGGAAAGCGG GCAGTGAGCG CAACGCAATT AATGTGAGTT AGCTCACTCA

1021 TTAGGCACCC CAGGCTTTAC ACTTTATGCT TCCGGCTCGT ATGTTGTGTG GAATTGTGAG

1081 CGGATAACAA TTTCACACAG GAAACAGCTA TGACCATGAT TACGCCAAGC TTGCATGCCT

1141 GCAGGTCCCC AGATTAGCCT TTTCAATTTC AGAAAGAATG CTAACCCACA GATGGTTAGA

1201 GAGGCTTACG CAGCAGGTCT CATCAAGACG ATCTACCCGA GCAATAATCT CCAGGAAATC

1261 AAATACCTTC CCAAGAAGGT TAAAGATGCA GTCAAAAGAT TCAGGACTAA CTGCATCAAG

1321 AACACAGAGA AAGATATATT TCTCAAGATC AGAAGTACTA TTCCAGTATG GACGATTCAA

1381 GGCTTGCTTC ACAAACCAAG GCAAGTAATA GAGATTGGAG TCTCTAAAAA GGTAGTTCCC

1441 ACTGAATCAA AGGCCATGGA GTCAAAGATT CAAATAGAGG ACCTAACAGA ACTCGCCGTA

1501 AAGACTGGCG AACAGTTCAT ACAGAGTCTC TTACGACTCA ATGACAAGAA GAAAATCTTC

1561 GTCAACATGG TGGAGCACGA CACACTTGTC TACTCCAAAA ATATCAAAGA TACAGTCTCA

1621 GAAGACCAAA GGGCAATTGA GACTTTTCAA CAAAGGGTAA TATCCGGAAA CCTCCTCGGA

1681 TTCCATTGCC CAGCTATCTG TCACTTTATT GTGAAGATAG TGGAAAAGGA AGGTGGCTCC

1741 TACAAATGCC ATCATTGCGA TAAAGGAAAG GCCATCGTTG AAGATGCCTC TGCCGACAGT

1801 GGTCCCAAAG ATGGACCCCC ACCCACGAGG AGCATCGTGG AAAAAGAAGA CGTTCCAACC

1861 ACGTCTTCAA AGCAAGTGGA TTGATGTGAT ATCTCCACTG ACGTAAGGGA TGACGCACAA

1921 TCCCACTATC CTTCGCAAGA CCCTTCCTCT ATATAAGGAA GTTCATTTCA TTTGGAGAGA

1981 ACACGCTCGA GAAAGATATT GTATATATCG TAACAATAGG AGGTTCAACA ATGGCTTCCT

2041 CCCCTCCAAA GAAAAAGAGA AAGGTTAGTT GGAAGGACGC AAGTGGTTGG TCGCGAGTGG

2101 ATCTACGCAC GCTCGGCTAC AGTCAGCAGC AGCAAGAGAA GATCAAACCG AAGGTGCGTT

2161 CGACAGTGGC GCAGCACCAC GAGGCACTGG TGGGCCATGG GTTTACACAC GCGCACATCG

2221 TTGCGCTCAG CCAACACCCG GCAGCGTTAG GGACCGTCGC TGTCACGTAT CAGCACATAA

2281 TCACGGCGTT GCCAGAGGCG ACACACGAAG ACATCGTTGG CGTCGGCAAA CAGTGGTCCG

2341 GCGCACGCGC CCTGGAGGCC TTGCTCACGG ATGCGGGGGA GTTGAGAGGT CCGCCGTTAC

2401 AGTTGGACAC AGGCCAACTT GTGAAGATTG CAAAACGTGG CGGCGTGACC GCAATGGAGG

2461 CAGTGCATGC ATCGCGCAAT GCACTGACGG GTGCCCCCCT GGAGACGATG GTGAGCAAGG

2521 GCGAGGAGCT GTTCACCGGG GTGGTGCCCA TCCTGGTCGA GCTGGACGGC GACGTAAACG

2581 GCCACAAGTT CAGCGTGTCC GGCGAGGGCG AGGGCGATGC CACCTACGGC AAGCTGACCC

2641 TGAAGTTCAT CTGCACCACC GGCAAGCTGC CCGTGCCCTG GCCCACCCTC GTGACCACCC

2701 TGACCTACGG CGTGCAGTGC TTCAGCCGCT ACCCCGACCA CATGAAGCAG CACGACTTCT

2761 TCAAGTCCGC CATGCCCGAA GGCTACGTCC AGGAGCGCAC CATCTTCTTC AAGGACGACG

2821 GCAACTACAA GACCCGCGCC GAGGTGAAGT TCGAGGGCGA CACCCTGGTG AACCGCATCG

2881 AGCTGAAGGG CATCGACTTC AAGGAGGACG GCAACATCCT GGGGCACAAG CTGGAGTACA

2941 ACTACAACAG CCACAACGTC TATATCATGG CCGACAAGCA GAAGAACGGC ATCAAGGTGA

3001 ACTTCAAGAT CCGCCACAAC ATCGAGGACG GCAGCGTGCA GCTCGCCGAC CACTACCAGC

3061 AGAACACCCC CATCGGCGAC GGCCCCGTGC TGCTGCCCGA CAACCACTAC CTGAGCACCC

3121 AGTCCGCCCT GAGCAAAGAC CCCAACGAGA AGCGCGATCA CATGGTCCTG CTGGAGTTCG

3181 TGACCGCCGC CGGGATCACT CTCGGCATGG ACGAGCTGTA CAAGTAACGT CTCCAACGAC

3241 CACCTCGTCG CCTTGGCCTG CCTCGGCGGA CGTCCTGCCA TGGATGCAGT GAAAAAGGGA

3301 TTGCCGCACG CGCCGGAATT GATCAGAAGA GTCAATCGCC GTATTGGCGA ACGCACGTCC

3361 CATCGCGTTG CCTCTAGATC CCAGCTAGTG AAATCTGAAT TGGAAGAGAA GAAATCTGAA

3421 CTTAGACATA AATTGAAATA TGTGCCACAT GAATATATTG AATTGATTGA AATCGCAAGA

3481 AATTCAACTC AGGATAGAAT CCTTGAAATG AAGGTGATGG AGTTCTTTAT GAAGGTTTAT

3541 GGTTATCGTG GTAAACATTT GGGTGGATCA AGGAAACCAG ACGGAGCAAT TTATACTGTC

3601 GGATCTCCTA TTGATTACGG TGTGATCGTT GATACTAAGG CATATTCAGG AGGTTATAAT

3661 CTTCCAATTG GTCAAGCAGA TGAAATGGAA AGATATGTCG AAGAGAATCA AACAAGAAAC

3721 AAGCATCTCA ACCCTAATGA ATGGTGGAAA GTCTATCCAT CTTCAGTAAC AGAATTTAAG

3781 TTCTTGTTTG TGAGTGGTCA TTTCAAAGGA AACTACAAAG CTCAGCTTAC AAGATTGAAT

3841 CATATCACTA ATTGTAATGG AGCTGTTCTT AGTGTAGAAG AGCTTTTGAT TGGTGGAGAA

3901 ATGATTAAAG CTGGTACATT GACACTTGAG GAAGTGAGAA GGAAATTTAA TAACGGTGAG

3961 ATAAACTTTT AATAGGAGCT CGGATCGTTC AAACATTTGG CAATAAAGTT TCTTAAGATT

4021 GAATCCTGTT GCCGGTCTTG CGATGATTAT CATATAATTT CTGTTGAATT ACGTTAAGCA

4081 TGTAATAATT AACATGTAAT GCATGACGTT ATTTATGAGA TGGGTTTTTA TGATTAGAGT

4141 CCCGCAATTA TACATTTAAT ACGCGATAGA AAACAAAATA TAGCGCGCAA ACTAGGATAA

4201 ATTATCGCGC GCGGTGTCAT CTATGTTACT AGATCGGGCG CGCCTTACGC GTCCCTAATA

4261 ATAATTAATT AAGAATCTCC AGAGGATCGC CGGGAACCGA GGACGAGTTC GTAATCATGG

4321 TCATAGCTGT TTCCTGTGTG AAATTGTTAT CCGCTCACAA TTCCACACAA CATACGAGCC

4381 GGAAGCATAA AGTGTAAAGC CTGGGGTGCC TAATGAGTGA GCTAACTCAC ATTAATTGCG

4441 TTGCGCTCAC TGCCCGCTTT CCAGTCGGGA AACCTGTCGT GCCAGCTGCA TTAATGAATC

4501 GGCCAACGCG CGGGGAGAGG CGGTTTGCGT ATTGGGCGCT CTTCCGCTTC CTCGCTCACT

4561 GACTCGCTGC GCTCGGTCGT TCGGCTGCGG CGAGCGGTAT CAGCTCACTC AAAGGCGGTA

4621 ATACGGTTAT CCACAGAATC AGGGGATAAC GCAGGAAAGA ACATGTGAGC AAAAGGCCAG

4681 CAAAAGGCCA GGAACCGTAA AAAGGCCGCG TTGCTGGCGT TTTTCCATAG GCTCCGCCCC

4741 CCTGACGAGC ATCACAAAAA TCGACGCTCA AGTCAGAGGT GGCGAAACCC GACAGGACTA

4801 TAAAGATACC AGGCGTTTCC CCCTGGAAGC TCCCTCGTGC GCTCTCCTGT TCCGACCCTG

4861 CCGCTTACCG GATACCTGTC CGCCTTTCTC CCTTCGGGAA GCGTGGCGCT TTCTCATAGC

4921 TCACGCTGTA GGTATCTCAG TTCGGTGTAG GTCGTTCGCT CCAAGCTGGG CTGTGTGCAC

4981 GAACCCCCCG TTCAGCCCGA CCGCTGCGCC TTATCCGGTA ACTATCGTCT TGAGTCCAAC

5041 CCGGTAAGAC ACGACTTATC GCCACTGGCA GCAGCCACTG GTAACAGGAT TAGCAGAGCG

5101 AGGTATGTAG GCGGTGCTAC AGAGTTCTTG AAGTGGTGGC CTAACTACGG CTACACTAGA

5161 AGAACAGTAT TTGGTATCTG CGCTCTGCTG AAGCCAGTTA CCTTCGGAAA AAGAGTTGGT

5221 AGCTCTTGAT CCGGCAAACA AACCACCGCT GGTAGCGGTG GTTTTTTTGT TTGCAAGCAG

5281 CAGATTACGC GCAGAAAAAA AGGATCTCAA GAAGATCCTT TGATCTTTTC TACGGGGTCT

5341 GACGCTCAGT GGAACGAAAA CTCACGTTAA GGGATTTTGG TCATGAGATT ATCAAAAAGG

5401 ATCTTCACCT AGATCCTTTT AAATTAAAAA TGAAGTTTTA AATCAATCTA AAGTATATAT

5461 GAGTAAACTT GGTCTGACAG TTACCAATGC TTAATCAGTG AGGCACCTAT CTCAGCGATC

5521 TGTCTATTTC GTTCATCCAT AGTTGCCTGA CTCCCCGTCG TGTAGATAAC TACGATACGG

5581 GAGGGCTTAC CATCTGGCCC CAGTGCTGCA ATGATACCGC GAGACCCACG CTCACCGGCT

5641 CCAGATTTAT CAGCAATAAA CCAGCCAGCC GGAAGGGCCG AGCGCAGAAG TGGTCCTGCA

5701 ACTTTATCCG CCTCCATCCA GTCTATTAAT TGTTGCCGGG AAGCTAGAGT AAGTAGTTCG

5761 CCAGTTAATA GTTTGCGCAA CGTTGTTGCC ATTGCTACAG GCATCGTGGT GTCACGCTCG

5821 TCGTTTGGTA TGGCTTCATT CAGCTCCGGT TCCCAACGAT CAAGGCGAGT TACATGATCC

5881 CCCATGTTGT GCAAAAAAGC GGTTAGCTCC TTCGGTCCTC CGATCGTTGT CAGAAGTAAG

5941 TTGGCCGCAG TGTTATCACT CATGGTTATG GCAGCACTGC ATAATTCTCT TACTGTCATG

6001 CCATCCGTAA GATGCTTTTC TGTGACTGGT GAGTACTCAA CCAAGTCATT CTGAGAATAG

6061 TGTATGCGGC GACCGAGTTG CTCTTGCCCG GCGTCAATAC GGGATAATAC CGCGCCACAT

6121 AGCAGAACTT TAAAAGTGCT CATCATTGGA AAACGTTCTT CGGGGCGAAA ACTCTCAAGG

6181 ATCTTACCGC TGTTGAGATC CAGTTCGATG TAACCCACTC GTGCACCCAA CTGATCTTCA

6241 GCATCTTTTA CTTTCACCAG CGTTTCTGGG TGAGCAAAAA CAGGAAGGCA AAATGCCGCA

6301 AAAAAGGGAA TAAGGGCGAC ACGGAAATGT TGAATACTCA TACTCTTCCT TTTTCAATAT

6361 TATTGAAGCA TTTATCAGGG TTATTGTCTC ATGAGCGGAT ACATATTTGA ATGTATTTAG

6421 AAAAATAAAC AAATAGGGGT TCCGCGCACA TTTCCCCGAA AAGTGCCACC TGACGTCTAA

6481 GAAACCATTA TTATCATGAC ATTAACCTAT AAAAATAGGC GTATCACGAG GCCCTTTCGA

6541 G

pMaL01-pUbi-GoldyTALEN:

1 TCGCGCGTTT CGGTGATGAC GGTGAAAACC TCTGACACAT GCAGCTCCCG GTCACGGTCA

61 CAGCTTGTCT GTAAGCGGAT GCCGGGAGCA GACAAGCCCG TCAGGGCGCG TCAGCGGGTG

121 TTGGCGGGTG TCGGGGCTGG CTTAACTATG CGGCATCAGA GCAGATTGTA CTGAGAGTGC

181 ACCATATGCG GTGTGAAATA CCGCACAGAT GCGTAAGGAG AAAATACCGC ATCAGGCGCC

241 ATTCGCCATT CAGGCTGCGC AACTGTTGGG AAGGGCGATC GGTGCGGGCC TCTTCGCTAT

301 TACGCCAGCT GGCGAAAGGG GGATGTGCTG CAAGGCGATT AAGTTGGGTA ACGCCAGGGT

361 TTTCCCAGTC ACGACGTTGT AAAACGACGG CCAGTGCCAA AGAAGCATGA CGGCAAGTGG

421 ACGATTCTTA ATTAAGGGGG GTTACCGGGT ACCAAGCTTG ATATCGAATT CCTGCAGTGC

481 AGCGTGACCC GGTCGTGCCC CTCTCTAGAG ATAATGAGCA TTGCATGTCT AAGTTATAAA

541 AAATTACCAC ATATTTTTTT TGTCACACTT GTTTGAAGTG CAGTTTATCT ATCTTTATAC

601 ATATATTTAA ACTTTACTCT ACGAATAATA TAATCTATAG TACTACAATA ATATCAGTGT

661 TTTAGAGAAT CATATAAATG AACAGTTAGA CATGGTCTAA AGGACAATTG AGTATTTTGA

721 CAACAGGACT CTACAGTTTT ATCTTTTTAG TGTGCATGTG TTCTCCTTTT TTTTTGCAAA

781 TAGCTTCACC TATATAATAC TTCATCCATT TTATTAGTAC ATCCATTTAG GGTTTAGGGT

841 TAATGGTTTT TATAGACTAA TTTTTTTAGT ACATCTATTT TATTCTATTT TAGCCTCTAA

901 ATTAAGAAAA CTAAAACTCT ATTTTAGTTT TTTTATTTAA TAATTTAGAT ATAAAATAGA

961 ATAAAATAAA GTGACTAAAA ATTAAACAAA TACCCTTTAA GAAATTAAAA AAACTAAGGA

1021 AACATTTTTC TTGTTTCGAG TAGATAATGC CAGCCTGTTA AACGCCGTCG ACGAGTCTAA

1081 CGGACACCAA CCAGCGAACC AGCAGCGTCG CGTCGGGCCA AGCGAAGCAG ACGGCACGGC

1141 ATCTCTGTCG CTGCCTCTGG ACCCCTCTCG AGAGTTCCGC TCCACCGTTG GACTTGCTCC

1201 GCTGTCGGCA TCCAGAAATG CGTGGCGGAG CGGCAGACGT GAGCCGGCAC GGCAGGCGGC

1261 CTCCTCCTCC TCTCACGGCA CGGCAGCTAC GGGGGATTCC TTTCCCACCG CTCCTTCGCT

1321 TTCCCTTCCT CGCCCGCCGT AATAAATAGA CACCCCCTCC ACACCCTCTT TCCCCAACCT

1381 CGTGTTGTTC GGAGCGCACA CACACACAAC CAGATCTCCC CCAAATCCAC CCGTCGGCAC

1441 CTCCGCTTCA AGGTACGCCG CTCGTCCTCC CCCCCCCCCC CTCTCTACCT TCTCTAGATC

1501 GGCGTTCCGG TCCATGGTTA GGGCCCGGTA GTTCTACTTC TGTTCATGTT TGTGTTAGAT

1561 CCGTGTTTGT GTTAGATCCG TGCTGCTAGC GTTCGTACAC GGATGCGACC TGTACGTCAG

1621 ACACGTTCTG ATTGCTAACT TGCCAGTGTT TCTCTTTGGG GAATCCTGGG ATGGCTCTAG

1681 CCGTTCCGCA GACGGGATCG ATTTCATGAT TTTTTTTGTT TCGTTGCATA GGGTTTGGTT

1741 TGCCCTTTTC CTTTATTTCA ATATATGCCG TGCACTTGTT TGTCGGGTCA TCTTTTCATG

1801 CTTTTTTTTG TCTTGGTTGT GATGATGTGG TCTGGTTGGG CGGTCGTTCT AGATCGGAGT

1861 AGAATTCTGT TTCAAACTAC CTGGTGGATT TATTAATTTT GGATCTGTAT GTGTGTGCCA

1921 TACATATTCA TAGTTACGAA TTGAAGATGA TGGATGGAAA TATCGATCTA GGATAGGTAT

1981 ACATGTTGAT GCGGGTTTTA CTGATGCATA TACAGAGATG CTTTTTGTTC GCTTGGTTGT

2041 GATGATGTGG TGTGGTTGGG CGGTCGTTCA TTCGTTCTAG ATCGGAGTAG AATACTGTTT

2101 CAAACTACCT GGTGTATTTA TTAATTTTGG AACTGTATGT GTGTGTCATA CATCTTCATA

2161 GTTACGAGTT TAAGATGGAT GGAAATATCG ATCTAGGATA GGTATACATG TTGATGTGGG

2221 TTTTACTGAT GCATATACAT GATGGCATAT GCAGCATCTA TTCATATGCT CTAACCTTGA

2281 GTACCTATCT ATTATAATAA ACAAGTATGT TTTATAATTA TTTTGATCTT GATATACTTG

2341 GATGATGGCA TATGCAGCAG CTATATGTGG ATTTTTTTAG CCCTGCCTTC ATACGCTATT

2401 TATTTGCTTG GTACTGTTTC TTTTGTCGAT GCTCACCCTG TTGTTTGGTG TTACTTCTGC

2461 AGCCCGGGCT CGAGAAAGAT ATTGTATATA TCGTAACAAT AGGAGGTTCA ACAATGGCTT

2521 CCTCCCCTCC AAAGAAAAAG AGAAAGGTTA GTTGGAAGGA CGCAAGTGGT TGGTCGCGAG

2581 TGGATCTACG CACGCTCGGC TACAGTCAGC AGCAGCAAGA GAAGATCAAA CCGAAGGTGC

2641 GTTCGACAGT GGCGCAGCAC CACGAGGCAC TGGTGGGCCA TGGGTTTACA CACGCGCACA

2701 TCGTTGCGCT CAGCCAACAC CCGGCAGCGT TAGGGACCGT CGCTGTCACG TATCAGCACA

2761 TAATCACGGC GTTGCCAGAG GCGACACACG AAGACATCGT TGGCGTCGGC AAACAGTGGT

2821 CCGGCGCACG CGCCCTGGAG GCCTTGCTCA CGGATGCGGG GGAGTTGAGA GGTCCGCCGT

2881 TACAGTTGGA CACAGGCCAA CTTGTGAAGA TTGCAAAACG TGGCGGCGTG ACCGCAATGG

2941 AGGCAGTGCA TGCATCGCGC AATGCACTGA CGGGTGCCCC CCTGGAGACG ATGGTGAGCA

3001 AGGGCGAGGA GCTGTTCACC GGGGTGGTGC CCATCCTGGT CGAGCTGGAC GGCGACGTAA

3061 ACGGCCACAA GTTCAGCGTG TCCGGCGAGG GCGAGGGCGA TGCCACCTAC GGCAAGCTGA

3121 CCCTGAAGTT CATCTGCACC ACCGGCAAGC TGCCCGTGCC CTGGCCCACC CTCGTGACCA

3181 CCCTGACCTA CGGCGTGCAG TGCTTCAGCC GCTACCCCGA CCACATGAAG CAGCACGACT

3241 TCTTCAAGTC CGCCATGCCC GAAGGCTACG TCCAGGAGCG CACCATCTTC TTCAAGGACG

3301 ACGGCAACTA CAAGACCCGC GCCGAGGTGA AGTTCGAGGG CGACACCCTG GTGAACCGCA

3361 TCGAGCTGAA GGGCATCGAC TTCAAGGAGG ACGGCAACAT CCTGGGGCAC AAGCTGGAGT

3421 ACAACTACAA CAGCCACAAC GTCTATATCA TGGCCGACAA GCAGAAGAAC GGCATCAAGG

3481 TGAACTTCAA GATCCGCCAC AACATCGAGG ACGGCAGCGT GCAGCTCGCC GACCACTACC

3541 AGCAGAACAC CCCCATCGGC GACGGCCCCG TGCTGCTGCC CGACAACCAC TACCTGAGCA

3601 CCCAGTCCGC CCTGAGCAAA GACCCCAACG AGAAGCGCGA TCACATGGTC CTGCTGGAGT

3661 TCGTGACCGC CGCCGGGATC ACTCTCGGCA TGGACGAGCT GTACAAGTAA CGTCTCCAAC

3721 GACCACCTCG TCGCCTTGGC CTGCCTCGGC GGACGTCCTG CCATGGATGC AGTGAAAAAG

3781 GGATTGCCGC ACGCGCCGGA ATTGATCAGA AGAGTCAATC GCCGTATTGG CGAACGCACG

3841 TCCCATCGCG TTGCCTCTAG ATCCCAGCTA GTGAAATCTG AATTGGAAGA GAAGAAATCT

3901 GAACTTAGAC ATAAATTGAA ATATGTGCCA CATGAATATA TTGAATTGAT TGAAATCGCA

3961 AGAAATTCAA CTCAGGATAG AATCCTTGAA ATGAAGGTGA TGGAGTTCTT TATGAAGGTT

4021 TATGGTTATC GTGGTAAACA TTTGGGTGGA TCAAGGAAAC CAGACGGAGC AATTTATACT

4081 GTCGGATCTC CTATTGATTA CGGTGTGATC GTTGATACTA AGGCATATTC AGGAGGTTAT

4141 AATCTTCCAA TTGGTCAAGC AGATGAAATG CAAAGATATG TCAAAGAGAA TCAAACAAGA

4201 AACAAGCATA TCAACCCTAA TGAATGGTGG AAAGTCTATC CATCTTCAGT AACAGAATTT

4261 AAGTTCTTGT TTGTGAGTGG TCATTTCAAA GGAAACTACA AAGCTCAGCT TACAAGATTG

4321 AATCATAAGA CTAATTGTAA TGGAGCTGTT CTTAGTGTAG AAGAGCTTTT GATTGGTGGA

4381 GAAATGATTA AAGCTGGTAC ATTGACACTT GAGGAAGTGA GAAGGAAATT TAATAACGGT

4441 GAGATAAACT TTTAATAGGA GCTCGGATCG TTCAAACATT TGGCAATAAA GTTTCTTAAG

4501 ATTGAATCCT GTTGCCGGTC TTGCGATGAT TATCATATAA TTTCTGTTGA ATTACGTTAA

4561 GCATGTAATA ATTAACATGT AATGCATGAC GTTATTTATG AGATGGGTTT TTATGATTAG

4621 AGTCCCGCAA TTATACATTT AATACGCGAT AGAAAACAAA ATATAGCGCG CAAACTAGGA

4681 TAAATTATCG CGCGCGGTGT CATCTATGTT ACTAGATCGG GCGCGCCTTG CGCGTCGGTA

4741 AATCATCTTA ATTAAGAATC TCCAGAGGAT CGCCGGGAAC CGAGGACGAG TTCGTAATCA

4801 TGGTCATAGC TGTTTCCTGT GTGAAATTGT TATCCGCTCA CAATTCCACA CAACATACGA

4861 GCCGGAAGCA TAAAGTGTAA AGCCTGGGGT GCCTAATGAG TGAGCTAACT CACATTAATT

4921 GCGTTGCGCT CACTGCCCGC TTTCCAGTCG GGAAACCTGT CGTGCCAGCT GCATTAATGA

4981 ATCGGCCAAC GCGCGGGGAG AGGCGGTTTG CGTATTGGGC GCTCTTCCGC TTCCTCGCTC

5041 ACTGACTCGC TGCGCTCGGT CGTTCGGCTG CGGCGAGCGG TATCAGCTCA CTCAAAGGCG

5101 GTAATACGGT TATCCACAGA ATCAGGGGAT AACGCAGGAA AGAACATGTG AGCAAAAGGC

5161 CAGCAAAAGG CCAGGAACCG TAAAAAGGCC GCGTTGCTGG CGTTTTTCCA TAGGCTCCGC

5221 CCCCCTGACG AGCATCACAA AAATCGACGC TCAAGTCAGA GGTGGCGAAA CCCGACAGGA

5281 CTATAAAGAT ACCAGGCGTT TCCCCCTGGA AGCTCCCTCG TGCGCTCTCC TGTTCCGACC

5341 CTGCCGCTTA CCGGATACCT GTCCGCCTTT CTCCCTTCGG GAAGCGTGGC GCTTTCTCAT

5401 AGCTCACGCT GTAGGTATCT CAGTTCGGTG TAGGTCGTTC GCTCCAAGCT GGGCTGTGTG

5461 CACGAACCCC CCGTTCAGCC CGACCGCTGC GCCTTATCCG GTAACTATCG TCTTGAGTCC

5521 AACCCGGTAA GACACGACTT ATCGCCACTG GCAGCAGCCA CTGGTAACAG GATTAGCAGA

5581 GCGAGGTATG TAGGCGGTGC TACAGAGTTC TTGAAGTGGT GGCCTAACTA CGGCTACACT

5641 AGAAGAACAG TATTTGGTAT CTGCGCTCTG CTGAAGCCAG TTACCTTCGG AAAAAGAGTT

5701 GGTAGCTCTT GATCCGGCAA ACAAACCACC GCTGGTAGCG GTGGTTTTTT TGTTTGCAAG

5761 CAGCAGATTA CGCGCAGAAA AAAAGGATCT CAAGAAGATC CTTTGATCTT TTCTACGGGG

5821 TCTGACGCTC AGTGGAACGA AAACTCACGT TAAGGGATTT TGGTCATGAG ATTATCAAAA

5881 AGGATCTTCA CCTAGATCCT TTTAAATTAA AAATGAAGTT TTAAATCAAT CTAAAGTATA

5941 TATGAGTAAA CTTGGTCTGA CAGTTACCAA TGCTTAATCA GTGAGGCACC TATCTCAGCG

6001 ATCTGTCTAT TTCGTTCATC CATAGTTGCC TGACTCCCCG TCGTGTAGAT AACTACGATA

6061 CGGGAGGGCT TACCATCTGG CCCCAGTGCT GCAATGATAC CGCGAGACCC ACGCTCACCG

6121 GCTCCAGATT TATCAGCAAT AAACCAGCCA GCCGGAAGGG CCGAGCGCAG AAGTGGTCCT

6181 GCAACTTTAT CCGCCTCCAT CCAGTCTATT AATTGTTGCC GGGAAGCTAG AGTAAGTAGT

6241 TCGCCAGTTA ATAGTTTGCG CAACGTTGTT GCCATTGCTA CAGGCATCGT GGTGTCACGC

6301 TCGTCGTTTG GTATGGCTTC ATTCAGCTCC GGTTCCCAAC GATCAAGGCG AGTTACATGA

6361 TCCCCCATGT TGTGCAAAAA AGCGGTTAGC TCCTTCGGTC CTCCGATCGT TGTCAGAAGT

6421 AAGTTGGCCG CAGTGTTATC ACTCATGGTT ATGGCAGCAC TGCATAATTC TCTTACTGTC

6481 ATGCCATCCG TAAGATGCTT TTCTGTGACT GGTGAGTACT CAACCAAGTC ATTCTGAGAA

6541 TAGTGTATGC GGCGACCGAG TTGCTCTTGC CCGGCGTCAA TACGGGATAA TACCGCGCCA

6601 CATAGCAGAA CTTTAAAAGT GCTCATCATT GGAAAACGTT CTTCGGGGCG AAAACTCTCA

6661 AGGATCTTAC CGCTGTTGAG ATCCAGTTCG ATGTAACCCA CTCGTGCACC CAACTGATCT

6721 TCAGCATCTT TTACTTTCAC CAGCGTTTCT GGGTGAGCAA AAACAGGAAG GCAAAATGCC

6781 GCAAAAAAGG GAATAAGGGC GACACGGAAA TGTTGAATAC TCATACTCTT CCTTTTTCAA

6841 TATTATTGAA GCATTTATCA GGGTTATTGT CTCATGAGCG GATACATATT TGAATGTATT

6901 TAGAAAAATA AACAAATAGG GGTTCCGCGC ACATTTCCCC GAAAAGTGCC ACCTGACGTC

6961 TAAGAAACCA TTATTATCAT GACATTAACC TATAAAAATA GGCGTATCAC GAGGCCCTTT

7021 CGAG

pMaL02-pUbi-GoldyTALEN:

1 TCGCGCGTTT CGGTGATGAC GGTGAAAACC TCTGACACAT GCAGCTCCCG GTCACGGTCA

61 CAGCTTGTCT GTAAGCGGAT GCCGGGAGCA GACAAGCCCG TCAGGGCGCG TCAGCGGGTG

121 TTGGCGGGTG TCGGGGCTGG CTTAACTATG CGGCATCAGA GCAGATTGTA CTGAGAGTGC

181 ACCATATGCG GTGTGAAATA CCGCACAGAT GCGTAAGGAG AAAATACCGC ATCAGGCGCC

241 ATTCGCCATT CAGGCTGCGC AACTGTTGGG AAGGGCGATC GGTGCGGGCC TCTTCGCTAT

301 TACGCCAGCT GGCGAAAGGG GGATGTGCTG CAAGGCGATT AAGTTGGGTA ACGCCAGGGT

361 TTTCCCAGTC ACGACGTTGT AAAACGACGG CCAGTGCCAA AGAAGCATGA CGGCAAGTGG

421 ACGATTCTTA ATTAAATCAT CTTAGGGGGT ACCAAGCTTG ATATCGAATT CCTGCAGTGC

481 AGCGTGACCC GGTCGTGCCC CTCTCTAGAG ATAATGAGCA TTGCATGTCT AAGTTATAAA

541 AAATTACCAC ATATTTTTTT TGTCACACTT GTTTGAAGTG CAGTTTATCT ATCTTTATAC

601 ATATATTTAA ACTTTACTCT ACGAATAATA TAATCTATAG TACTACAATA ATATCAGTGT

661 TTTAGAGAAT CATATAAATG AACAGTTAGA CATGGTCTAA AGGACAATTG AGTATTTTGA

721 CAACAGGACT CTACAGTTTT ATCTTTTTAG TGTGCATGTG TTCTCCTTTT TTTTTGCAAA

781 TAGCTTCACC TATATAATAC TTCATCCATT TTATTAGTAC ATCCATTTAG GGTTTAGGGT

841 TAATGGTTTT TATAGACTAA TTTTTTTAGT ACATCTATTT TATTCTATTT TAGCCTCTAA

901 ATTAAGAAAA CTAAAACTCT ATTTTAGTTT TTTTATTTAA TAATTTAGAT ATAAAATAGA

961 ATAAAATAAA GTGACTAAAA ATTAAACAAA TACCCTTTAA GAAATTAAAA AAACTAAGGA

1021 AACATTTTTC TTGTTTCGAG TAGATAATGC CAGCCTGTTA AACGCCGTCG ACGAGTCTAA

1081 CGGACACCAA CCAGCGAACC AGCAGCGTCG CGTCGGGCCA AGCGAAGCAG ACGGCACGGC

1141 ATCTCTGTCG CTGCCTCTGG ACCCCTCTCG AGAGTTCCGC TCCACCGTTG GACTTGCTCC

1201 GCTGTCGGCA TCCAGAAATG CGTGGCGGAG CGGCAGACGT GAGCCGGCAC GGCAGGCGGC

1261 CTCCTCCTCC TCTCACGGCA CGGCAGCTAC GGGGGATTCC TTTCCCACCG CTCCTTCGCT

1321 TTCCCTTCCT CGCCCGCCGT AATAAATAGA CACCCCCTCC ACACCCTCTT TCCCCAACCT

1381 CGTGTTGTTC GGAGCGCACA CACACACAAC CAGATCTCCC CCAAATCCAC CCGTCGGCAC

1441 CTCCGCTTCA AGGTACGCCG CTCGTCCTCC CCCCCCCCCC CTCTCTACCT TCTCTAGATC

1501 GGCGTTCCGG TCCATGGTTA GGGCCCGGTA GTTCTACTTC TGTTCATGTT TGTGTTAGAT

1561 CCGTGTTTGT GTTAGATCCG TGCTGCTAGC GTTCGTACAC GGATGCGACC TGTACGTCAG

1621 ACACGTTCTG ATTGCTAACT TGCCAGTGTT TCTCTTTGGG GAATCCTGGG ATGGCTCTAG

1681 CCGTTCCGCA GACGGGATCG ATTTCATGAT TTTTTTTGTT TCGTTGCATA GGGTTTGGTT

1741 TGCCCTTTTC CTTTATTTCA ATATATGCCG TGCACTTGTT TGTCGGGTCA TCTTTTCATG

1801 CTTTTTTTTG TCTTGGTTGT GATGATGTGG TCTGGTTGGG CGGTCGTTCT AGATCGGAGT

1861 AGAATTCTGT TTCAAACTAC CTGGTGGATT TATTAATTTT GGATCTGTAT GTGTGTGCCA

1921 TACATATTCA TAGTTACGAA TTGAAGATGA TGGATGGAAA TATCGATCTA GGATAGGTAT

1981 ACATGTTGAT GCGGGTTTTA CTGATGCATA TACAGAGATG CTTTTTGTTC GCTTGGTTGT

2041 GATGATGTGG TGTGGTTGGG CGGTCGTTCA TTCGTTCTAG ATCGGAGTAG AATACTGTTT

2101 CAAACTACCT GGTGTATTTA TTAATTTTGG AACTGTATGT GTGTGTCATA CATCTTCATA

2161 GTTACGAGTT TAAGATGGAT GGAAATATCG ATCTAGGATA GGTATACATG TTGATGTGGG

2221 TTTTACTGAT GCATATACAT GATGGCATAT GCAGCATCTA TTCATATGCT CTAACCTTGA

2281 GTACCTATCT ATTATAATAA ACAAGTATGT TTTATAATTA TTTTGATCTT GATATACTTG

2341 GATGATGGCA TATGCAGCAG CTATATGTGG ATTTTTTTAG CCCTGCCTTC ATACGCTATT

2401 TATTTGCTTG GTACTGTTTC TTTTGTCGAT GCTCACCCTG TTGTTTGGTG TTACTTCTGC

2461 AGCCCGGGCT CGAGAAAGAT ATTGTATATA TCGTAACAAT AGGAGGTTCA ACAATGGCTT

2521 CCTCCCCTCC AAAGAAAAAG AGAAAGGTTA GTTGGAAGGA CGCAAGTGGT TGGTCGCGAG

2581 TGGATCTACG CACGCTCGGC TACAGTCAGC AGCAGCAAGA GAAGATCAAA CCGAAGGTGC

2641 GTTCGACAGT GGCGCAGCAC CACGAGGCAC TGGTGGGCCA TGGGTTTACA CACGCGCACA

2701 TCGTTGCGCT CAGCCAACAC CCGGCAGCGT TAGGGACCGT CGCTGTCACG TATCAGCACA

2761 TAATCACGGC GTTGCCAGAG GCGACACACG AAGACATCGT TGGCGTCGGC AAACAGTGGT

2821 CCGGCGCACG CGCCCTGGAG GCCTTGCTCA CGGATGCGGG GGAGTTGAGA GGTCCGCCGT

2881 TACAGTTGGA CACAGGCCAA CTTGTGAAGA TTGCAAAACG TGGCGGCGTG ACCGCAATGG

2941 AGGCAGTGCA TGCATCGCGC AATGCACTGA CGGGTGCCCC CCTGGAGACG ATGGTGAGCA

3001 AGGGCGAGGA GCTGTTCACC GGGGTGGTGC CCATCCTGGT CGAGCTGGAC GGCGACGTAA

3061 ACGGCCACAA GTTCAGCGTG TCCGGCGAGG GCGAGGGCGA TGCCACCTAC GGCAAGCTGA

3121 CCCTGAAGTT CATCTGCACC ACCGGCAAGC TGCCCGTGCC CTGGCCCACC CTCGTGACCA

3181 CCCTGACCTA CGGCGTGCAG TGCTTCAGCC GCTACCCCGA CCACATGAAG CAGCACGACT

3241 TCTTCAAGTC CGCCATGCCC GAAGGCTACG TCCAGGAGCG CACCATCTTC TTCAAGGACG

3301 ACGGCAACTA CAAGACCCGC GCCGAGGTGA AGTTCGAGGG CGACACCCTG GTGAACCGCA

3361 TCGAGCTGAA GGGCATCGAC TTCAAGGAGG ACGGCAACAT CCTGGGGCAC AAGCTGGAGT

3421 ACAACTACAA CAGCCACAAC GTCTATATCA TGGCCGACAA GCAGAAGAAC GGCATCAAGG

3481 TGAACTTCAA GATCCGCCAC AACATCGAGG ACGGCAGCGT GCAGCTCGCC GACCACTACC

3541 AGCAGAACAC CCCCATCGGC GACGGCCCCG TGCTGCTGCC CGACAACCAC TACCTGAGCA

3601 CCCAGTCCGC CCTGAGCAAA GACCCCAACG AGAAGCGCGA TCACATGGTC CTGCTGGAGT

3661 TCGTGACCGC CGCCGGGATC ACTCTCGGCA TGGACGAGCT GTACAAGTAA CGTCTCCAAC

3721 GACCACCTCG TCGCCTTGGC CTGCCTCGGC GGACGTCCTG CCATGGATGC AGTGAAAAAG

3781 GGATTGCCGC ACGCGCCGGA ATTGATCAGA AGAGTCAATC GCCGTATTGG CGAACGCACG

3841 TCCCATCGCG TTGCCTCTAG ATCCCAGCTA GTGAAATCTG AATTGGAAGA GAAGAAATCT

3901 GAACTTAGAC ATAAATTGAA ATATGTGCCA CATGAATATA TTGAATTGAT TGAAATCGCA

3961 AGAAATTCAA CTCAGGATAG AATCCTTGAA ATGAAGGTGA TGGAGTTCTT TATGAAGGTT

4021 TATGGTTATC GTGGTAAACA TTTGGGTGGA TCAAGGAAAC CAGACGGAGC AATTTATACT

4081 GTCGGATCTC CTATTGATTA CGGTGTGATC GTTGATACTA AGGCATATTC AGGAGGTTAT

4141 AATCTTCCAA TTGGTCAAGC AGATGAAATG GAAAGATATG TCGAAGAGAA TCAAACAAGA

4201 AACAAGCATC TCAACCCTAA TGAATGGTGG AAAGTCTATC CATCTTCAGT AACAGAATTT

4261 AAGTTCTTGT TTGTGAGTGG TCATTTCAAA GGAAACTACA AAGCTCAGCT TACAAGATTG

4321 AATCATATCA CTAATTGTAA TGGAGCTGTT CTTAGTGTAG AAGAGCTTTT GATTGGTGGA

4381 GAAATGATTA AAGCTGGTAC ATTGACACTT GAGGAAGTGA GAAGGAAATT TAATAACGGT

4441 GAGATAAACT TTTAATAGGA GCTCGGATCG TTCAAACATT TGGCAATAAA GTTTCTTAAG

4501 ATTGAATCCT GTTGCCGGTC TTGCGATGAT TATCATATAA TTTCTGTTGA ATTACGTTAA

4561 GCATGTAATA ATTAACATGT AATGCATGAC GTTATTTATG AGATGGGTTT TTATGATTAG

4621 AGTCCCGCAA TTATACATTT AATACGCGAT AGAAAACAAA ATATAGCGCG CAAACTAGGA

4681 TAAATTATCG CGCGCGGTGT CATCTATGTT ACTAGATCGG GCGCGCCTTA CGCGTCCCTA

4741 ATAATAATTA ATTAAGAATC TCCAGAGGAT CGCCGGGAAC CGAGGACGAG TTCGTAATCA

4801 TGGTCATAGC TGTTTCCTGT GTGAAATTGT TATCCGCTCA CAATTCCACA CAACATACGA

4861 GCCGGAAGCA TAAAGTGTAA AGCCTGGGGT GCCTAATGAG TGAGCTAACT CACATTAATT

4921 GCGTTGCGCT CACTGCCCGC TTTCCAGTCG GGAAACCTGT CGTGCCAGCT GCATTAATGA

4981 ATCGGCCAAC GCGCGGGGAG AGGCGGTTTG CGTATTGGGC GCTCTTCCGC TTCCTCGCTC

5041 ACTGACTCGC TGCGCTCGGT CGTTCGGCTG CGGCGAGCGG TATCAGCTCA CTCAAAGGCG

5101 GTAATACGGT TATCCACAGA ATCAGGGGAT AACGCAGGAA AGAACATGTG AGCAAAAGGC

5161 CAGCAAAAGG CCAGGAACCG TAAAAAGGCC GCGTTGCTGG CGTTTTTCCA TAGGCTCCGC

5221 CCCCCTGACG AGCATCACAA AAATCGACGC TCAAGTCAGA GGTGGCGAAA CCCGACAGGA

5281 CTATAAAGAT ACCAGGCGTT TCCCCCTGGA AGCTCCCTCG TGCGCTCTCC TGTTCCGACC

5341 CTGCCGCTTA CCGGATACCT GTCCGCCTTT CTCCCTTCGG GAAGCGTGGC GCTTTCTCAT

5401 AGCTCACGCT GTAGGTATCT CAGTTCGGTG TAGGTCGTTC GCTCCAAGCT GGGCTGTGTG

5461 CACGAACCCC CCGTTCAGCC CGACCGCTGC GCCTTATCCG GTAACTATCG TCTTGAGTCC

5521 AACCCGGTAA GACACGACTT ATCGCCACTG GCAGCAGCCA CTGGTAACAG GATTAGCAGA

5581 GCGAGGTATG TAGGCGGTGC TACAGAGTTC TTGAAGTGGT GGCCTAACTA CGGCTACACT

5641 AGAAGAACAG TATTTGGTAT CTGCGCTCTG CTGAAGCCAG TTACCTTCGG AAAAAGAGTT

5701 GGTAGCTCTT GATCCGGCAA ACAAACCACC GCTGGTAGCG GTGGTTTTTT TGTTTGCAAG

5761 CAGCAGATTA CGCGCAGAAA AAAAGGATCT CAAGAAGATC CTTTGATCTT TTCTACGGGG

5821 TCTGACGCTC AGTGGAACGA AAACTCACGT TAAGGGATTT TGGTCATGAG ATTATCAAAA

5881 AGGATCTTCA CCTAGATCCT TTTAAATTAA AAATGAAGTT TTAAATCAAT CTAAAGTATA

5941 TATGAGTAAA CTTGGTCTGA CAGTTACCAA TGCTTAATCA GTGAGGCACC TATCTCAGCG

6001 ATCTGTCTAT TTCGTTCATC CATAGTTGCC TGACTCCCCG TCGTGTAGAT AACTACGATA

6061 CGGGAGGGCT TACCATCTGG CCCCAGTGCT GCAATGATAC CGCGAGACCC ACGCTCACCG

6121 GCTCCAGATT TATCAGCAAT AAACCAGCCA GCCGGAAGGG CCGAGCGCAG AAGTGGTCCT

6181 GCAACTTTAT CCGCCTCCAT CCAGTCTATT AATTGTTGCC GGGAAGCTAG AGTAAGTAGT

6241 TCGCCAGTTA ATAGTTTGCG CAACGTTGTT GCCATTGCTA CAGGCATCGT GGTGTCACGC

6301 TCGTCGTTTG GTATGGCTTC ATTCAGCTCC GGTTCCCAAC GATCAAGGCG AGTTACATGA

6361 TCCCCCATGT TGTGCAAAAA AGCGGTTAGC TCCTTCGGTC CTCCGATCGT TGTCAGAAGT

6421 AAGTTGGCCG CAGTGTTATC ACTCATGGTT ATGGCAGCAC TGCATAATTC TCTTACTGTC

6481 ATGCCATCCG TAAGATGCTT TTCTGTGACT GGTGAGTACT CAACCAAGTC ATTCTGAGAA

6541 TAGTGTATGC GGCGACCGAG TTGCTCTTGC CCGGCGTCAA TACGGGATAA TACCGCGCCA

6601 CATAGCAGAA CTTTAAAAGT GCTCATCATT GGAAAACGTT CTTCGGGGCG AAAACTCTCA

6661 AGGATCTTAC CGCTGTTGAG ATCCAGTTCG ATGTAACCCA CTCGTGCACC CAACTGATCT

6721 TCAGCATCTT TTACTTTCAC CAGCGTTTCT GGGTGAGCAA AAACAGGAAG GCAAAATGCC

6781 GCAAAAAAGG GAATAAGGGC GACACGGAAA TGTTGAATAC TCATACTCTT CCTTTTTCAA

6841 TATTATTGAA GCATTTATCA GGGTTATTGT CTCATGAGCG GATACATATT TGAATGTATT

6901 TAGAAAAATA AACAAATAGG GGTTCCGCGC ACATTTCCCC GAAAAGTGCC ACCTGACGTC

6961 TAAGAAACCA TTATTATCAT GACATTAACC TATAAAAATA GGCGTATCAC GAGGCCCTTT

7021 CGAG

pTALEN-DES01

1 CATGCCAACC ACAGGGTTCC CCTCGGGATC AAAGTACTTT GATCCAACCC CTCCGCTGCT

61 ATAGTGCAGT CGGCTTCTGA CGTTCAGTGC AGCCGTCTTC TGAAAACGAC ATGTCGCACA

121 AGTCCTAAGT TACGCGACAG GCTGCCGCCC TGCCCTTTTC CTGGCGTTTT CTTGTCGCGT

181 GTTTTAGTCG CATAAAGTAG AATACTTGCG ACTAGAACCG GAGACATTAC GCCATGAACA

241 AGAGCGCCGC CGCTGGCCTG CTGGGCTATG CCCGCGTCAG CACCGACGAC CAGGACTTGA

301 CCAACCAACG GGCCGAACTG CACGCGGCCG GCTGCACCAA GCTGTTTTCC GAGAAGATCA

361 CCGGCACCAG GCGCGACCGC CCGGAGCTGG CCAGGATGCT TGACCACCTA CGCCCTGGCG

421 ACGTTGTGAC AGTGACCAGG CTAGACCGCC TGGCCCGCAG CACCCGCGAC CTACTGGACA

481 TTGCCGAGCG CATCCAGGAG GCCGGCGCGG GCCTGCGTAG CCTGGCAGAG CCGTGGGCCG

541 ACACCACCAC GCCGGCCGGC CGCATGGTGT TGACCGTGTT CGCCGGCATT GCCGAGTTCG

601 AGCGTTCCCT AATCATCGAC CGCACCCGGA GCGGGCGCGA GGCCGCCAAG GCCCGAGGCG

661 TGAAGTTTGG CCCCCGCCCT ACCCTCACCC CGGCACAGAT CGCGCACGCC CGCGAGCTGA

721 TCGACCAGGA AGGCCGCACC GTGAAAGAGG CGGCTGCACT GCTTGGCGTG CATCGCTCGA

781 CCCTGTACCG CGCACTTGAG CGCAGCGAGG AAGTGACGCC CACCGAGGCC AGGCGGCGCG

841 GTGCCTTCCG TGAGGACGCA TTGACCGAGG CCGACGCCCT GGCGGCCGCC GAGAATGAAC

901 GCCAAGAGGA ACAAGCATGA AACCGCACCA GGACGGCCAG GACGAACCGT TTTTCATTAC

961 CGAAGAGATC GAGGCGGAGA TGATCGCGGC CGGGTACGTG TTCGAGCCGC CCGCGCACGT

1021 CTCAACCGTG CGGCTGCATG AAATCCTGGC CGGTTTGTCT GATGCCAAGC TGGCGGCCTG

1081 GCCGGCCAGC TTGGCCGCTG AAGAAACCGA GCGCCGCCGT CTAAAAAGGT GATGTGTATT

1141 TGAGTAAAAC AGCTTGCGTC ATGCGGTCGC TGCGTATATG ATGCGATGAG TAAATAAACA

1201 AATACGCAAG GGGAACGCAT GAAGGTTATC GCTGTACTTA ACCAGAAAGG CGGGTCAGGC

1261 AAGACGACCA TCGCAACCCA TCTAGCCCGC GCCCTGCAAC TCGCCGGGGC CGATGTTCTG

1321 TTAGTCGATT CCGATCCCCA GGGCAGTGCC CGCGATTGGG CGGCCGTGCG GGAAGATCAA

1381 CCGCTAACCG TTGTCGGCAT CGACCGCCCG ACGATTGACC GCGACGTGAA GGCCATCGGC

1441 CGGCGCGACT TCGTAGTGAT CGACGGAGCG CCCCAGGCGG CGGACTTGGC TGTGTCCGCG

1501 ATCAAGGCAG CCGACTTCGT GCTGATTCCG GTGCAGCCAA GCCCTTACGA CATATGGGCC

1561 ACCGCCGACC TGGTGGAGCT GGTTAAGCAG CGCATTGAGG TCACGGATGG AAGGCTACAA

1621 GCGGCCTTTG TCGTGTCGCG GGCGATCAAA GGCACGCGCA TCGGCGGTGA GGTTGCCGAG

1681 GCGCTGGCCG GGTACGAGCT GCCCATTCTT GAGTCCCGTA TCACGCAGCG CGTGAGCTAC

1741 CCAGGCACTG CCGCCGCCGG CACAACCGTT CTTGAATCAG AACCCGAGGG CGACGCTGCC

1801 CGCGAGGTCC AGGCGCTGGC CGCTGAAATT AAATCAAAAC TCATTTGAGT TAATGAGGTA

1861 AAGAGAAAAT GAGCAAAAGC ACAAACACGC TAAGTGCCGG CCGTCCGAGC GCACGCAGCA

1921 GCAAGGCTGC AACGTTGGCC AGCCTGGCAG ACACGCCAGC CATGAAGCGG GTCAACTTTC

1981 AGTTGCCGGC GGAGGATCAC ACCAAGCTGA AGATGTACGC GGTACGCCAA GGCAAGACCA

2041 TTACCGAGCT GCTATCTGAA TACATCGCGC AGCTACCAGA GTAAATGAGC AAATGAATAA

2101 ATGAGTAGAT GAATTTTAGC GGCTAAAGGA GGCGGCATGG AAAATCAAGA ACAACCAGGC

2161 ACCGACGCCG TGGAATGCCC CATGTGTGGA GGAACGGGCG GTTGGCCAGG CGTAAGCGGC

2221 TGGGTTGTCT GCCGGCCCTG CAATGGCACT GGAACCCCCA AGCCCGAGGA ATCGGCGTGA

2281 CGGTCGCAAA CCATCCGGCC CGGTACAAAT CGGCGCGGCG CTGGGTGATG ACCTGGTGGA

2341 GAAGTTGAAG GCCGCGCAGG CCGCCCAGCG GCAACGCATC GAGGCAGAAG CACGCCCCGG

2401 TGAATCGTGG CAAGCGGCCG CTGATCGAAT CCGCAAAGAA TCCCGGCAAC CGCCGGCAGC

2461 CGGTGCGCCG TCGATTAGGA AGCCGCCCAA GGGCGACGAG CAACCAGATT TTTTCGTTCC

2521 GATGCTCTAT GACGTGGGCA CCCGCGATAG TCGCAGCATC ATGGACGTGG CCGTTTTCCG

2581 TCTGTCGAAG CGTGACCGAC GAGCTGGCGA GGTGATCCGC TACGAGCTTC CAGACGGGCA

2641 CGTAGAGGTT TCCGCAGGGC CGGCCGGCAT GGCCAGTGTG TGGGATTACG ACCTGGTACT

2701 GATGGCGGTT TCCCATCTAA CCGAATCCAT GAACCGATAC CGGGAAGGGA AGGGAGACAA

2761 GCCCGGCCGC GTGTTCCGTC CACACGTTGC GGACGTACTC AAGTTCTGCC GGCGAGCCGA

2821 TGGCGGAAAG CAGAAAGACG ACCTGGTAGA AACCTGCATT CGGTTAAACA CCACGCACGT

2881 TGCCATGCAG CGTACGAAGA AGGCCAAGAA CGGCCGCCTG GTGACGGTAT CCGAGGGTGA

2941 AGCCTTGATT AGCCGCTACA AGATCGTAAA GAGCGAAACC GGGCGGCCGG AGTACATCGA

3001 GATCGAGCTA GCTGATTGGA TGTACCGCGA GATCACAGAA GGCAAGAACC CGGACGTGCT

3061 GACGGTTCAC CCCGATTACT TTTTGATCGA TCCCGGCATC GGCCGTTTTC TCTACCGCCT

3121 GGCACGCCGC GCCGCAGGCA AGGCAGAAGC CAGATGGTTG TTCAAGACGA TCTACGAACG

3181 CAGTGGCAGC GCCGGAGAGT TCAAGAAGTT CTGTTTCACC GTGCGCAAGC TGATCGGGTC

3241 AAATGACCTG CCGGAGTACG ATTTGAAGGA GGAGGCGGGG CAGGCTGGCC CGATCCTAGT

3301 CATGCGCTAC CGCAACCTGA TCGAGGGCGA AGCATCCGCC GGTTCCTAAT GTACGGAGCA

3361 GATGCTAGGG CAAATTGCCC TAGCAGGGGA AAAAGGTCGA AAAGGTCTCT TTCCTGTGGA

3421 TAGCACGTAC ATTGGGAACC CAAAGCCGTA CATTGGGAAC CGGAACCCGT ACATTGGGAA

3481 CCCAAAGCCG TACATTGGGA ACCGGTCACA CATGTAAGTG ACTGATATAA AAGAGAAAAA

3541 AGGCGATTTT TCCGCCTAAA ACTCTTTAAA ACTTATTAAA ACTCTTAAAA CCCGCCTGGC

3601 CTGTGCATAA CTGTCTGGCC AGCGCACAGC CGAAGAGCTG CAAAAAGCGC CTACCCTTCG

3661 GTCGCTGCGC TCCCTACGCC CCGCCGCTTC GCGTCGGCCT ATCGCGGCCG CTGGCCGCTC

3721 AAAAATGGCT GGCCTACGGC CAGGCAATCT ACCAGGGCGC GGACAAGCCG CGCCGTCGCC

3781 ACTCGACCGC CGGCGCCCAC ATCAAGGCAC CCTGCCTCGC GCGTTTCGGT GATGACGGTG

3841 AAAACCTCTG ACACATGCAG CTCCCGGAGA CGGTCACAGC TTGTCTGTAA GCGGATGCCG

3901 GGAGCAGACA AGCCCGTCAG GGCGCGTCAG CGGGTGTTGG CGGGTGTCGG GGCGCAGCCA

3961 TGACCCAGTC ACGTAGCGAT AGCGGAGTGT ATACTGGCTT AACTATGCGG CATCAGAGCA

4021 GATTGTACTG AGAGTGCACC ATATGCGGTG TGAAATACCG CACAGATGCG TAAGGAGAAA

4081 ATACCGCATC AGGCGCTCTT CCGCTTCCTC GCTCACTGAC TCGCTGCGCT CGGTCGTTCG

4141 GCTGCGGCGA GCGGTATCAG CTCACTCAAA GGCGGTAATA CGGTTATCCA CAGAATCAGG

4201 GGATAACGCA GGAAAGAACA TGTGAGCAAA AGGCCAGCAA AAGGCCAGGA ACCGTAAAAA

4261 GGCCGCGTTG CTGGCGTTTT TCCATAGGCT CCGCCCCCCT GACGAGCATC ACAAAAATCG

4321 ACGCTCAAGT CAGAGGTGGC GAAACCCGAC AGGACTATAA AGATACCAGG CGTTTCCCCC

4381 TGGAAGCTCC CTCGTGCGCT CTCCTGTTCC GACCCTGCCG CTTACCGGAT ACCTGTCCGC

4441 CTTTCTCCCT TCGGGAAGCG TGGCGCTTTC TCATAGCTCA CGCTGTAGGT ATCTCAGTTC

4501 GGTGTAGGTC GTTCGCTCCA AGCTGGGCTG TGTGCACGAA CCCCCCGTTC AGCCCGACCG

4561 CTGCGCCTTA TCCGGTAACT ATCGTCTTGA GTCCAACCCG GTAAGACACG ACTTATCGCC

4621 ACTGGCAGCA GCCACTGGTA ACAGGATTAG CAGAGCGAGG TATGTAGGCG GTGCTACAGA

4681 GTTCTTGAAG TGGTGGCCTA ACTACGGCTA CACTAGAAGG ACAGTATTTG GTATCTGCGC

4741 TCTGCTGAAG CCAGTTACCT TCGGAAAAAG AGTTGGTAGC TCTTGATCCG GCAAACAAAC

4801 CACCGCTGGT AGCGGTGGTT TTTTTGTTTG CAAGCAGCAG ATTACGCGCA GAAAAAAAGG

4861 ATCTCAAGAA GATCCTTTGA TCTTTTCTAC GGGGTCTGAC GCTCAGTGGA ACGAAAACTC

4921 ACGTTAAGGG ATTTTGGTCA TGCATTCTAG GTACTAAAAC AATTCATCCA GTAAAATATA

4981 ATATTTTATT TTCTCCCAAT CAGGCTTGAT CCCCAGTAAG TCAAAAAATA GCTCGACATA

5041 CTGTTCTTCC CCGATATCCT CCCTGATCGA CCGGACGCAG AAGGCAATGT CATACCACTT

5101 GTCCGCCCTG CCGCTTCTCC CAAGATCAAT AAAGCCACTT ACTTTGCCAT CTTTCACAAA

5161 GATGTTGCTG TCTCCCAGGT CGCCGTGGGA AAAGACAAGT TCCTCTTCGG GCTTTTCCGT

5221 CTTTAAAAAA TCATACAGCT CGCGCGGATC TTTAAATGGA GTGTCTTCTT CCCAGTTTTC

5281 GCAATCCACA TCGGCCAGAT CGTTATTCAG TAAGTAATCC AATTCGGCTA AGCGGCTGTC

5341 TAAGCTATTC GTATAGGGAC AATCCGATAT GTCGATGGAG TGAAAGAGCC TGATGCACTC

5401 CGCATACAGC TCGATAATCT TTTCAGGGCT TTGTTCATCT TCATACTCTT CCGAGCAAAG

5461 GACGCCATCG GCCTCACTCA TGAGCAGATT GCTCCAGCCA TCATGCCGTT CAAAGTGCAG

5521 GACCTTTGGA ACAGGCAGCT TTCCTTCCAG CCATAGCATC ATGTCCTTTT CCCGTTCCAC

5581 ATCATAGGTG GTCCCTTTAT ACCGGCTGTC CGTCATTTTT AAATATAGGT TTTCATTTTC

5641 TCCCACCAGC TTATATACCT TAGCAGGAGA CATTCCTTCC GTATCTTTTA CGCAGCGGTA

5701 TTTTTCGATC AGTTTTTTCA ATTCCGGTGA TATTCTCATT TTAGCCATTT ATTATTTCCT

5761 TCCTCTTTTC TACAGTATTT AAAGATACCC CAAGAAGCTA ATTATAACAA GACGAACTCC

5821 AATTCACTGT TCCTTGCATT CTAAAACCTT AAATACCAGA AAACAGCTTT TTCAAAGTTG

5881 TTTTCAAAGT TGGCGTATAA CATAGTATCG ACGGAGCCGA TTTTGAAACC GCGGTGATCA

5941 CAGGCAGCAA CGCTCTGTCA TCGTTACAAT CAACATGCTA CCCTCCGCGA GATCATCCGT

6001 GTTTCAAACC CGGCAGCTTA GTTGCCGTTC TTCCGAATAG CATCGGTAAC ATGAGCAAAG

6061 TCTGCCGCCT TACAACGGCT CTCCCGCTGA CGCCGTCCCG GACTGATGGG CTGCCTGTAT

6121 CGAGTGGTGA TTTTGTGCCG AGCTGCCGGT CGGGGAGCTG TTGGCTGGCT GGTGGCAGGA

6181 TATATTGTGG TGTAAACAAA TTGACGCTTA GACAACTTAA TAACACATTG CGGACGTTTT

6241 TAATGTACTG AATTAACGCC GAATTAATTC GGGGGATCTG GATTTTAGTA CTGGATTTTG

6301 GTTTTAGGAA TTAGAAATTT TATTGATAGA AGTATTTTAC AAATACAAAT ACATACTAAG

6361 GGTTTCTTAT ATGCTCAACA CATGAGCGAA ACCCTATAGG AACCCTAATT CCCTTATCTG

6421 GGAACTACTC ACACATTATT ATGGAGAAAC TCGAGCTTGT CGATCGACTC TAGCTAGAGG

6481 ATCGATCCGA ACCCCAGAGT CCCGCTCAGA AGAACTCGTC AAGAAGGCGA TAGAAGGCGA

6541 TGCGCTGCGA ATCGGGAGCG GCGATACCGT AAAGCACGAG GAAGCGGTCA GCCCATTCGC

6601 CGCCAAGCTC TTCAGCAATA TCACGGGTAG CCAACGCTAT GTCCTGATAG CGGTCCGCCA

6661 CACCCAGCCG GCCACAGTCG ATGAATCCAG AAAAGCGGCC ATTTTCCACC ATGATATTCG

6721 GCAAGCAGGC ATCGCCATGT GTCACGACGA GATCCTCGCC GTCGGGCATG CGCGCCTTGA

6781 GCCTGGCGAA CAGTTCGGCT GGCGCGAGCC CCTGATGCTC TTCGTCCAGA TCATCCTGAT

6841 CGACAAGACC GGCTTCCATC CGAGTACGTG CTCGCTCGAT GCGATGTTTC GCTTGGTGGT

6901 CGAATGGGCA GGTAGCCGGA TCAAGCGTAT GCAGCCGCCG CATTGCATCA GCCATGATGG

6961 ATACTTTCTC GGCAGGAGCA AGGTGAGATG ACAGGAGATC CTGCCCCGGC ACTTCGCCCA

7021 ATAGCAGCCA GTCCCTTCCC GCTTCAGTGA CAACGTCGAG CACAGCTGCG CAAGGAACGC

7081 CCGTCGTGGC CAGCCACGAT AGCCGCGCTG CCTCGTCCTG GAGTTCATTC AGGGCACCGG

7141 ACAGGTCGGT CTTGACAAAA AGAACCGGGC GCCCCTGCGC TGACAGCCGG AACACGGCGG

7201 CATCAGAGCA GCCGATTGTC TGTTGTGCCC AGTCATAGCC GAATAGCCTC TCCACCCAAG

7261 CGGCCGGAGA ACCTGCGTGC AATCCATCTT GTTCAATCCC CATGGTCGAT CGACAGATCT

7321 GCGAAAGCTC GAGAGAGATA GATTTGTAGA GAGAGACTGG TGATTTCAGC GTGTCCTCTC

7381 CAAATGAAAT GAACTTCCTT ATATAGAGGA AGGTCTTGCG AAGGATAGTG GGATTGTGCG

7441 TCATCCCTTA CGTCAGTGGA GATATCACAT CAATCCACTT GCTTTGAAGA CGTGGTTGGA

7501 ACGTCTTCTT TTTCCACGAT GCTCCTCGTG GGTGGGGGTC CATCTTTGGG ACCACTGTCG

7561 GCAGAGGCAT CTTGAACGAT AGCCTTTCCT TTATCGCAAT GATGGCATTT GTAGGTGCCA

7621 CCTTCCTTTT CTACTGTCCT TTTGATGAAG TGACAGATAG CTGGGCAATG GAATCCGAGG

7681 AGGTTTCCCG ATATTACCCT TTGTTGAAAA GTCTCAATAG CCCTTTGGTC TTCTGAGACT

7741 GTATCTTTGA TATTCTTGGA GTAGACGAGA GTGTCGTGCT CCACCATGTT ATCACATCAA

7801 TCCACTTGCT TTGAAGACGT GGTTGGAACG TCTTCTTTTT CCACGATGCT CCTCGTGGGT

7861 GGGGGTCCAT CTTTGGGACC ACTGTCGGCA GAGGCATCTT GAACGATAGC CTTTCCTTTA

7921 TCGCAATGAT GGCATTTGTA GGTGCCACCT TCCTTTTCTA CTGTCCTTTT GATGAAGTGA

7981 CAGATAGCTG GGCAATGGAA TCCGAGGAGG TTTCCCGATA TTACCCTTTG TTGAAAAGTC

8041 TCAATAGCCC TTTGGTCTTC TGAGACTGTA TCTTTGATAT TCTTGGAGTA GACGAGAGTG

8101 TCGTGCTCCA CCATGTTGGC AAGCTGCTCT AGCCAATACG CAAACCGCCT CTCCCCGCGC

8161 GTTGGCCGAT TCATTAATGC AGCTGGCACG ACAGGTTTCC CGACTGGAAA GCGGGCAGTG

8221 AGCGCAACGC AATTAATGTG AGTTAGCTCA CTCATTAGGC ACCCCAGGCT TTACACTTTA

8281 TGCTTCCGGC TCGTATGTTG TGTGGAATTG TGAGCGGATA ACAATTTCAC ACAGGAAACA

8341 GCTATGACCA TGATTACGAA TTCACCGGTC CTAAGGGGGG TTAATTAATA ATAATTAGGA

8401 CTAGTCAAGC TTGGCACTGG CCGTCGTTTT ACAACGTCGT GACTGGGAAA ACCCTGGCGT

8461 TACCCAACTT AATCGCCTTG CAGCACATCC CCCTTTCGCC AGCTGGCGTA ATAGCGAAGA

8521 GGCCCGCACC GATCGCCCTT CCCAACAGTT GCGCAGCCTG AATGGCGAAT GCTAGAGCAG

8581 CTTGAGCTTG GATCAGATTG TCGTTTCCCG CCTTCAGTTT AAACTATCAG TGTTTGACAG

8641 GATATATTGG CGGGTAAACC TAAGAGAAAA GAGCGTTTAT TAGAATAACG GATATTTAAA

8701 AGGGCGTGAA AAGGTTTATC CGTTCGTCCA TTTGTATGTG

pTALEN-DES02

1 CATGCCAACC ACAGGGTTCC CCTCGGGATC AAAGTACTTT GATCCAACCC CTCCGCTGCT

61 ATAGTGCAGT CGGCTTCTGA CGTTCAGTGC AGCCGTCTTC TGAAAACGAC ATGTCGCACA

121 AGTCCTAAGT TACGCGACAG GCTGCCGCCC TGCCCTTTTC CTGGCGTTTT CTTGTCGCGT

181 GTTTTAGTCG CATAAAGTAG AATACTTGCG ACTAGAACCG GAGACATTAC GCCATGAACA

241 AGAGCGCCGC CGCTGGCCTG CTGGGCTATG CCCGCGTCAG CACCGACGAC CAGGACTTGA

301 CCAACCAACG GGCCGAACTG CACGCGGCCG GCTGCACCAA GCTGTTTTCC GAGAAGATCA

361 CCGGCACCAG GCGCGACCGC CCGGAGCTGG CCAGGATGCT TGACCACCTA CGCCCTGGCG

421 ACGTTGTGAC AGTGACCAGG CTAGACCGCC TGGCCCGCAG CACCCGCGAC CTACTGGACA

481 TTGCCGAGCG CATCCAGGAG GCCGGCGCGG GCCTGCGTAG CCTGGCAGAG CCGTGGGCCG

541 ACACCACCAC GCCGGCCGGC CGCATGGTGT TGACCGTGTT CGCCGGCATT GCCGAGTTCG

601 AGCGTTCCCT AATCATCGAC CGCACCCGGA GCGGGCGCGA GGCCGCCAAG GCCCGAGGCG

661 TGAAGTTTGG CCCCCGCCCT ACCCTCACCC CGGCACAGAT CGCGCACGCC CGCGAGCTGA

721 TCGACCAGGA AGGCCGCACC GTGAAAGAGG CGGCTGCACT GCTTGGCGTG CATCGCTCGA

781 CCCTGTACCG CGCACTTGAG CGCAGCGAGG AAGTGACGCC CACCGAGGCC AGGCGGCGCG

841 GTGCCTTCCG TGAGGACGCA TTGACCGAGG CCGACGCCCT GGCGGCCGCC GAGAATGAAC

901 GCCAAGAGGA ACAAGCATGA AACCGCACCA GGACGGCCAG GACGAACCGT TTTTCATTAC

961 CGAAGAGATC GAGGCGGAGA TGATCGCGGC CGGGTACGTG TTCGAGCCGC CCGCGCACGT

1021 CTCAACCGTG CGGCTGCATG AAATCCTGGC CGGTTTGTCT GATGCCAAGC TGGCGGCCTG

1081 GCCGGCCAGC TTGGCCGCTG AAGAAACCGA GCGCCGCCGT CTAAAAAGGT GATGTGTATT

1141 TGAGTAAAAC AGCTTGCGTC ATGCGGTCGC TGCGTATATG ATGCGATGAG TAAATAAACA

1201 AATACGCAAG GGGAACGCAT GAAGGTTATC GCTGTACTTA ACCAGAAAGG CGGGTCAGGC

1261 AAGACGACCA TCGCAACCCA TCTAGCCCGC GCCCTGCAAC TCGCCGGGGC CGATGTTCTG

1321 TTAGTCGATT CCGATCCCCA GGGCAGTGCC CGCGATTGGG CGGCCGTGCG GGAAGATCAA

1381 CCGCTAACCG TTGTCGGCAT CGACCGCCCG ACGATTGACC GCGACGTGAA GGCCATCGGC

1441 CGGCGCGACT TCGTAGTGAT CGACGGAGCG CCCCAGGCGG CGGACTTGGC TGTGTCCGCG

1501 ATCAAGGCAG CCGACTTCGT GCTGATTCCG GTGCAGCCAA GCCCTTACGA CATATGGGCC

1561 ACCGCCGACC TGGTGGAGCT GGTTAAGCAG CGCATTGAGG TCACGGATGG AAGGCTACAA

1621 GCGGCCTTTG TCGTGTCGCG GGCGATCAAA GGCACGCGCA TCGGCGGTGA GGTTGCCGAG

1681 GCGCTGGCCG GGTACGAGCT GCCCATTCTT GAGTCCCGTA TCACGCAGCG CGTGAGCTAC

1741 CCAGGCACTG CCGCCGCCGG CACAACCGTT CTTGAATCAG AACCCGAGGG CGACGCTGCC

1801 CGCGAGGTCC AGGCGCTGGC CGCTGAAATT AAATCAAAAC TCATTTGAGT TAATGAGGTA

1861 AAGAGAAAAT GAGCAAAAGC ACAAACACGC TAAGTGCCGG CCGTCCGAGC GCACGCAGCA

1921 GCAAGGCTGC AACGTTGGCC AGCCTGGCAG ACACGCCAGC CATGAAGCGG GTCAACTTTC

1981 AGTTGCCGGC GGAGGATCAC ACCAAGCTGA AGATGTACGC GGTACGCCAA GGCAAGACCA

2041 TTACCGAGCT GCTATCTGAA TACATCGCGC AGCTACCAGA GTAAATGAGC AAATGAATAA

2101 ATGAGTAGAT GAATTTTAGC GGCTAAAGGA GGCGGCATGG AAAATCAAGA ACAACCAGGC

2161 ACCGACGCCG TGGAATGCCC CATGTGTGGA GGAACGGGCG GTTGGCCAGG CGTAAGCGGC

2221 TGGGTTGTCT GCCGGCCCTG CAATGGCACT GGAACCCCCA AGCCCGAGGA ATCGGCGTGA

2281 CGGTCGCAAA CCATCCGGCC CGGTACAAAT CGGCGCGGCG CTGGGTGATG ACCTGGTGGA

2341 GAAGTTGAAG GCCGCGCAGG CCGCCCAGCG GCAACGCATC GAGGCAGAAG CACGCCCCGG

2401 TGAATCGTGG CAAGCGGCCG CTGATCGAAT CCGCAAAGAA TCCCGGCAAC CGCCGGCAGC

2461 CGGTGCGCCG TCGATTAGGA AGCCGCCCAA GGGCGACGAG CAACCAGATT TTTTCGTTCC

2521 GATGCTCTAT GACGTGGGCA CCCGCGATAG TCGCAGCATC ATGGACGTGG CCGTTTTCCG

2581 TCTGTCGAAG CGTGACCGAC GAGCTGGCGA GGTGATCCGC TACGAGCTTC CAGACGGGCA

2641 CGTAGAGGTT TCCGCAGGGC CGGCCGGCAT GGCCAGTGTG TGGGATTACG ACCTGGTACT

2701 GATGGCGGTT TCCCATCTAA CCGAATCCAT GAACCGATAC CGGGAAGGGA AGGGAGACAA

2761 GCCCGGCCGC GTGTTCCGTC CACACGTTGC GGACGTACTC AAGTTCTGCC GGCGAGCCGA

2821 TGGCGGAAAG CAGAAAGACG ACCTGGTAGA AACCTGCATT CGGTTAAACA CCACGCACGT

2881 TGCCATGCAG CGTACGAAGA AGGCCAAGAA CGGCCGCCTG GTGACGGTAT CCGAGGGTGA

2941 AGCCTTGATT AGCCGCTACA AGATCGTAAA GAGCGAAACC GGGCGGCCGG AGTACATCGA

3001 GATCGAGCTA GCTGATTGGA TGTACCGCGA GATCACAGAA GGCAAGAACC CGGACGTGCT

3061 GACGGTTCAC CCCGATTACT TTTTGATCGA TCCCGGCATC GGCCGTTTTC TCTACCGCCT

3121 GGCACGCCGC GCCGCAGGCA AGGCAGAAGC CAGATGGTTG TTCAAGACGA TCTACGAACG

3181 CAGTGGCAGC GCCGGAGAGT TCAAGAAGTT CTGTTTCACC GTGCGCAAGC TGATCGGGTC

3241 AAATGACCTG CCGGAGTACG ATTTGAAGGA GGAGGCGGGG CAGGCTGGCC CGATCCTAGT

3301 CATGCGCTAC CGCAACCTGA TCGAGGGCGA AGCATCCGCC GGTTCCTAAT GTACGGAGCA

3361 GATGCTAGGG CAAATTGCCC TAGCAGGGGA AAAAGGTCGA AAAGGTCTCT TTCCTGTGGA

3421 TAGCACGTAC ATTGGGAACC CAAAGCCGTA CATTGGGAAC CGGAACCCGT ACATTGGGAA

3481 CCCAAAGCCG TACATTGGGA ACCGGTCACA CATGTAAGTG ACTGATATAA AAGAGAAAAA

3541 AGGCGATTTT TCCGCCTAAA ACTCTTTAAA ACTTATTAAA ACTCTTAAAA CCCGCCTGGC

3601 CTGTGCATAA CTGTCTGGCC AGCGCACAGC CGAAGAGCTG CAAAAAGCGC CTACCCTTCG

3661 GTCGCTGCGC TCCCTACGCC CCGCCGCTTC GCGTCGGCCT ATCGCGGCCG CTGGCCGCTC

3721 AAAAATGGCT GGCCTACGGC CAGGCAATCT ACCAGGGCGC GGACAAGCCG CGCCGTCGCC

3781 ACTCGACCGC CGGCGCCCAC ATCAAGGCAC CCTGCCTCGC GCGTTTCGGT GATGACGGTG

3841 AAAACCTCTG ACACATGCAG CTCCCGGAGA CGGTCACAGC TTGTCTGTAA GCGGATGCCG

3901 GGAGCAGACA AGCCCGTCAG GGCGCGTCAG CGGGTGTTGG CGGGTGTCGG GGCGCAGCCA

3961 TGACCCAGTC ACGTAGCGAT AGCGGAGTGT ATACTGGCTT AACTATGCGG CATCAGAGCA

4021 GATTGTACTG AGAGTGCACC ATATGCGGTG TGAAATACCG CACAGATGCG TAAGGAGAAA

4081 ATACCGCATC AGGCGCTCTT CCGCTTCCTC GCTCACTGAC TCGCTGCGCT CGGTCGTTCG

4141 GCTGCGGCGA GCGGTATCAG CTCACTCAAA GGCGGTAATA CGGTTATCCA CAGAATCAGG

4201 GGATAACGCA GGAAAGAACA TGTGAGCAAA AGGCCAGCAA AAGGCCAGGA ACCGTAAAAA

4261 GGCCGCGTTG CTGGCGTTTT TCCATAGGCT CCGCCCCCCT GACGAGCATC ACAAAAATCG

4321 ACGCTCAAGT CAGAGGTGGC GAAACCCGAC AGGACTATAA AGATACCAGG CGTTTCCCCC

4381 TGGAAGCTCC CTCGTGCGCT CTCCTGTTCC GACCCTGCCG CTTACCGGAT ACCTGTCCGC

4441 CTTTCTCCCT TCGGGAAGCG TGGCGCTTTC TCATAGCTCA CGCTGTAGGT ATCTCAGTTC

4501 GGTGTAGGTC GTTCGCTCCA AGCTGGGCTG TGTGCACGAA CCCCCCGTTC AGCCCGACCG

4561 CTGCGCCTTA TCCGGTAACT ATCGTCTTGA GTCCAACCCG GTAAGACACG ACTTATCGCC

4621 ACTGGCAGCA GCCACTGGTA ACAGGATTAG CAGAGCGAGG TATGTAGGCG GTGCTACAGA

4681 GTTCTTGAAG TGGTGGCCTA ACTACGGCTA CACTAGAAGG ACAGTATTTG GTATCTGCGC

4741 TCTGCTGAAG CCAGTTACCT TCGGAAAAAG AGTTGGTAGC TCTTGATCCG GCAAACAAAC

4801 CACCGCTGGT AGCGGTGGTT TTTTTGTTTG CAAGCAGCAG ATTACGCGCA GAAAAAAAGG

4861 ATCTCAAGAA GATCCTTTGA TCTTTTCTAC GGGGTCTGAC GCTCAGTGGA ACGAAAACTC

4921 ACGTTAAGGG ATTTTGGTCA TGCATTCTAG GTACTAAAAC AATTCATCCA GTAAAATATA

4981 ATATTTTATT TTCTCCCAAT CAGGCTTGAT CCCCAGTAAG TCAAAAAATA GCTCGACATA

5041 CTGTTCTTCC CCGATATCCT CCCTGATCGA CCGGACGCAG AAGGCAATGT CATACCACTT

5101 GTCCGCCCTG CCGCTTCTCC CAAGATCAAT AAAGCCACTT ACTTTGCCAT CTTTCACAAA

5161 GATGTTGCTG TCTCCCAGGT CGCCGTGGGA AAAGACAAGT TCCTCTTCGG GCTTTTCCGT

5221 CTTTAAAAAA TCATACAGCT CGCGCGGATC TTTAAATGGA GTGTCTTCTT CCCAGTTTTC

5281 GCAATCCACA TCGGCCAGAT CGTTATTCAG TAAGTAATCC AATTCGGCTA AGCGGCTGTC

5341 TAAGCTATTC GTATAGGGAC AATCCGATAT GTCGATGGAG TGAAAGAGCC TGATGCACTC

5401 CGCATACAGC TCGATAATCT TTTCAGGGCT TTGTTCATCT TCATACTCTT CCGAGCAAAG

5461 GACGCCATCG GCCTCACTCA TGAGCAGATT GCTCCAGCCA TCATGCCGTT CAAAGTGCAG

5521 GACCTTTGGA ACAGGCAGCT TTCCTTCCAG CCATAGCATC ATGTCCTTTT CCCGTTCCAC

5581 ATCATAGGTG GTCCCTTTAT ACCGGCTGTC CGTCATTTTT AAATATAGGT TTTCATTTTC

5641 TCCCACCAGC TTATATACCT TAGCAGGAGA CATTCCTTCC GTATCTTTTA CGCAGCGGTA

5701 TTTTTCGATC AGTTTTTTCA ATTCCGGTGA TATTCTCATT TTAGCCATTT ATTATTTCCT

5761 TCCTCTTTTC TACAGTATTT AAAGATACCC CAAGAAGCTA ATTATAACAA GACGAACTCC

5821 AATTCACTGT TCCTTGCATT CTAAAACCTT AAATACCAGA AAACAGCTTT TTCAAAGTTG

5881 TTTTCAAAGT TGGCGTATAA CATAGTATCG ACGGAGCCGA TTTTGAAACC GCGGTGATCA

5941 CAGGCAGCAA CGCTCTGTCA TCGTTACAAT CAACATGCTA CCCTCCGCGA GATCATCCGT

6001 GTTTCAAACC CGGCAGCTTA GTTGCCGTTC TTCCGAATAG CATCGGTAAC ATGAGCAAAG

6061 TCTGCCGCCT TACAACGGCT CTCCCGCTGA CGCCGTCCCG GACTGATGGG CTGCCTGTAT

6121 CGAGTGGTGA TTTTGTGCCG AGCTGCCGGT CGGGGAGCTG TTGGCTGGCT GGTGGCAGGA

6181 TATATTGTGG TGTAAACAAA TTGACGCTTA GACAACTTAA TAACACATTG CGGACGTTTT

6241 TAATGTACTG AATTAACGCC GAATTAATTC GGGGGATCTG GATTTTAGTA CTGGATTTTG

6301 GTTTTAGGAA TTAGAAATTT TATTGATAGA AGTATTTTAC AAATACAAAT ACATACTAAG

6361 GGTTTCTTAT ATGCTCAACA CATGAGCGAA ACCCTATAGG AACCCTAATT CCCTTATCTG

6421 GGAACTACTC ACACATTATT ATGGAGAAAC TCGAGCTTGT CGATCGACTC TAGCTAGAGG

6481 ATCGATCCGA ACCCCAGAGT CCCGCTCAGA AGAACTCGTC AAGAAGGCGA TAGAAGGCGA

6541 TGCGCTGCGA ATCGGGAGCG GCGATACCGT AAAGCACGAG GAAGCGGTCA GCCCATTCGC

6601 CGCCAAGCTC TTCAGCAATA TCACGGGTAG CCAACGCTAT GTCCTGATAG CGGTCCGCCA

6661 CACCCAGCCG GCCACAGTCG ATGAATCCAG AAAAGCGGCC ATTTTCCACC ATGATATTCG

6721 GCAAGCAGGC ATCGCCATGT GTCACGACGA GATCCTCGCC GTCGGGCATG CGCGCCTTGA

6781 GCCTGGCGAA CAGTTCGGCT GGCGCGAGCC CCTGATGCTC TTCGTCCAGA TCATCCTGAT

6841 CGACAAGACC GGCTTCCATC CGAGTACGTG CTCGCTCGAT GCGATGTTTC GCTTGGTGGT

6901 CGAATGGGCA GGTAGCCGGA TCAAGCGTAT GCAGCCGCCG CATTGCATCA GCCATGATGG

6961 ATACTTTCTC GGCAGGAGCA AGGTGAGATG ACAGGAGATC CTGCCCCGGC ACTTCGCCCA

7021 ATAGCAGCCA GTCCCTTCCC GCTTCAGTGA CAACGTCGAG CACAGCTGCG CAAGGAACGC

7081 CCGTCGTGGC CAGCCACGAT AGCCGCGCTG CCTCGTCCTG GAGTTCATTC AGGGCACCGG

7141 ACAGGTCGGT CTTGACAAAA AGAACCGGGC GCCCCTGCGC TGACAGCCGG AACACGGCGG

7201 CATCAGAGCA GCCGATTGTC TGTTGTGCCC AGTCATAGCC GAATAGCCTC TCCACCCAAG

7261 CGGCCGGAGA ACCTGCGTGC AATCCATCTT GTTCAATCCC CATGGTCGAT CGACAGATCT

7321 GCGAAAGCTC GAGAGAGATA GATTTGTAGA GAGAGACTGG TGATTTCAGC GTGTCCTCTC

7381 CAAATGAAAT GAACTTCCTT ATATAGAGGA AGGTCTTGCG AAGGATAGTG GGATTGTGCG

7441 TCATCCCTTA CGTCAGTGGA GATATCACAT CAATCCACTT GCTTTGAAGA CGTGGTTGGA

7501 ACGTCTTCTT TTTCCACGAT GCTCCTCGTG GGTGGGGGTC CATCTTTGGG ACCACTGTCG

7561 GCAGAGGCAT CTTGAACGAT AGCCTTTCCT TTATCGCAAT GATGGCATTT GTAGGTGCCA

7621 CCTTCCTTTT CTACTGTCCT TTTGATGAAG TGACAGATAG CTGGGCAATG GAATCCGAGG

7681 AGGTTTCCCG ATATTACCCT TTGTTGAAAA GTCTCAATAG CCCTTTGGTC TTCTGAGACT

7741 GTATCTTTGA TATTCTTGGA GTAGACGAGA GTGTCGTGCT CCACCATGTT ATCACATCAA

7801 TCCACTTGCT TTGAAGACGT GGTTGGAACG TCTTCTTTTT CCACGATGCT CCTCGTGGGT

7861 GGGGGTCCAT CTTTGGGACC ACTGTCGGCA GAGGCATCTT GAACGATAGC CTTTCCTTTA

7921 TCGCAATGAT GGCATTTGTA GGTGCCACCT TCCTTTTCTA CTGTCCTTTT GATGAAGTGA

7981 CAGATAGCTG GGCAATGGAA TCCGAGGAGG TTTCCCGATA TTACCCTTTG TTGAAAAGTC

8041 TCAATAGCCC TTTGGTCTTC TGAGACTGTA TCTTTGATAT TCTTGGAGTA GACGAGAGTG

8101 TCGTGCTCCA CCATGTTGGC AAGCTGCTCT AGCCAATACG CAAACCGCCT CTCCCCGCGC

8161 GTTGGCCGAT TCATTAATGC AGCTGGCACG ACAGGTTTCC CGACTGGAAA GCGGGCAGTG

8221 AGCGCAACGC AATTAATGTG AGTTAGCTCA CTCATTAGGC ACCCCAGGCT TTACACTTTA

8281 TGCTTCCGGC TCGTATGTTG TGTGGAATTG TGAGCGGATA ACAATTTCAC ACAGGAAACA

8341 GCTATGACCA TGATTACGAA TTCACTAGTC CTAAGGGGGG TTAATTAATA ATAATTAGGC

8401 CTGCAGGCAA GCTTGGCACT GGCCGTCGTT TTACAACGTC GTGACTGGGA AAACCCTGGC

8461 GTTACCCAAC TTAATCGCCT TGCAGCACAT CCCCCTTTCG CCAGCTGGCG TAATAGCGAA

8521 GAGGCCCGCA CCGATCGCCC TTCCCAACAG TTGCGCAGCC TGAATGGCGA ATGCTAGAGC

8581 AGCTTGAGCT TGGATCAGAT TGTCGTTTCC CGCCTTCAGT TTAAACTATC AGTGTTTGAC

8641 AGGATATATT GGCGGGTAAA CCTAAGAGAA AAGAGCGTTT ATTAGAATAA CGGATATTTA

8701 AAAGGGCGTG AAAAGGTTTA TCCGTTCGTC CATTTGTATG TG

pTALEN-DES03

1 TGAGCGTCGC AAAGGCGCTC GGTCTTGCCT TGCTCGTCGG TGATGTACTT CACCAGCTCC

61 GCGAAGTCGC TCTTCTTGAT GGAGCGCATG GGGACGTGCT TGGCAATCAC GCGCACCCCC

121 CGGCCGTTTT AGCGGCTAAA AAAGTCATGG CTCTGCCCTC GGGCGGACCA CGCCCATCAT

181 GACCTTGCCA AGCTCGTCCT GCTTCTCTTC GATCTTCGCC AGCAGGGCGA GGATCGTGGC

241 ATCACCGAAC CGCGCCGTGC GCGGGTCGTC GGTGAGCCAG AGTTTCAGCA GGCCGCCCAG

301 GCGGCCCAGG TCGCCATTGA TGCGGGCCAG CTCGCGGACG TGCTCATAGT CCACGACGCC

361 CGTGATTTTG TAGCCCTGGC CGACGGCCAG CAGGTAGGCC GACAGGCTCA TGCCGGCCGC

421 CGCCGCCTTT TCCTCAATCG CTCTTCGTTC GTCTGGAAGG CAGTACACCT TGATAGGTGG

481 GCTGCCCTTC CTGGTTGGCT TGGTTTCATC AGCCATCCGC TTGCCCTCAT CTGTTACGCC

541 GGCGGTAGCC GGCCAGCCTC GCAGAGCAGG ATTCCCGTTG AGCACCGCCA GGTGCGAATA

601 AGGGACAGTG AAGAAGGAAC ACCCGCTCGC GGGTGGGCCT ACTTCACCTA TCCTGCCCGG

661 CTGACGCCGT TGGATACACC AAGGAAAGTC TACACGAACC CTTTGGCAAA ATCCTGTATA

721 TCGTGCGAAA AAGGATGGAT ATACCGAAAA AATCGCTATA ATGACCCCGA AGCAGGGTTA

781 TGCAGCGGAA AAGCGCCACG CTTCCCGAAG GGAGAAAGGC GGACAGGTAT CCGGTAAGCG

841 GCAGGGTCGG AACAGGAGAG CGCACGAGGG AGCTTCCAGG GGGAAACGCC TGGTATCTTT

901 ATAGTCCTGT CGGGTTTCGC CACCTCTGAC TTGAGCGTCG ATTTTTGTGA TGCTCGTCAG

961 GGGGGCGGAG CCTATGGAAA AACGCCAGCA ACGCGGCCTT TTTACGGTTC CTGGCCTTTT

1021 GCTGGCCTTT TGCTCACATG TTCTTTCCTG CGTTATCCCC TGATTCTGTG GATAACCGTA

1081 TTACCGCCTT TGAGTGAGCT GATACCGCTC GCCGCAGCCG AACGACCGAG CGCAGCGAGT

1141 CAGTGAGCGA GGAAGCGGAA GAGCGCCAGA AGGCCGCCAG AGAGGCCGAG CGCGGCCGTG

1201 AGGCTTGGAC GCTAGGGCAG GGCATGAAAA AGCCCGTAGC GGGCTGCTAC GGGCGTCTGA

1261 CGCGGTGGAA AGGGGGAGGG GATGTTGTCT ACATGGCTCT GCTGTAGTGA GTGGGTTGCG

1321 CTCCGGCAGC GGTCCTGATC AATCGTCACC CTTTCTCGGT CCTTCAACGT TCCTGACAAC

1381 GAGCCTCCTT TTCGCCAATC CATCGACAAT CACCGCGAGT CCCTGCTCGA ACGCTGCGTC

1441 CGGACCGGCT TCGTCGAAGG CGTCTATCGC GGCCCGCAAC AGCGGCGAGA GCGGAGCCTG

1501 TTCAACGGTG CCGCCGCGCT CGCCGGCATC GCTGTCGCCG GCCTGCTCCT CAAGCACGGC

1561 CCCAACAGTG AAGTAGCTGA TTGTCATCAG CGCATTGACG GCGTCCCCGG CCGAAAAACC

1621 CGCCTCGCAG AGGAAGCGAA GCTGCGCGTC GGCCGTTTCC ATCTGCGGTG CGCCCGGTCG

1681 CGTGCCGGCA TGGATGCGCG CGCCATCGCG GTAGGCGAGC AGCGCCTGCC TGAAGCTGCG

1741 GGCATTCCCG ATCAGAAATG AGCGCCAGTC GTCGTCGGCT CTCGGCACCG AATGCGTATG

1801 ATTCTCCGCC AGCATGGCTT CGGCCAGTGC GTCGAGCAGC GCCCGCTTGT TCCTGAAGTG

1861 CCAGTAAAGC GCCGGCTGCT GAACCCCCAA CCGTTCCGCC AGTTTGCGTG TCGTCAGACC

1921 GTCTACGCCG ACCTCGTTCA ACAGGTCCAG GGCGGCACGG ATCACTGTAT TCGGCTGCAA

1981 CTTTGTCATG CTTGACACTT TATCACTGAT AAACATAATA TGTCCACCAA CTTATCAGTG

2041 ATAAAGAATC CGCGCGTTCA ATCGGACCAG CGGAGGCTGG TCCGGAGGCC AGACGTGAAA

2101 CCCAACATAC CCCTGATCGT AATTCTGAGC ACTGTCGCGC TCGACGCTGT CGGCATCGGC

2161 CTGATTATGC CGGTGCTGCC GGGCCTCCTG CGCGATCTGG TTCACTCGAA CGACGTCACC

2221 GCCCACTATG GCATTCTGCT GGCGCTGTAT GCGTTGGTGC AATTTGCCTG CGCACCTGTG

2281 CTGGGCGCGC TGTCGGATCG TTTCGGGCGG CGGCCAATCT TGCTCGTCTC GCTGGCCGGC

2341 GCCAGATCTG GGGAACCCTG TGGTTGGCAT GCACATACAA ATGGACGAAC GGATAAACCT

2401 TTTCACGCCC TTTTAAATAT CCGATTATTC TAATAAACGC TCTTTTCTCT TAGGTTTACC

2461 CGCCAATATA TCCTGTCAAA CACTGATAGT TTGGGTACCG AGCTCGAATT CGTAATCATG

2521 GTCATAGCTG TTTCCTGTGT GAAATTGTTA TCCGCTCACA ATTCCACACA ACATACGAGC

2581 CGGAAGCATA AAGTGTAAAG CCTGGGGTGC CTAATGAGTG AGCTAACTCA CATTAATTGC

2641 GTTGCGCTCA CTGCCCGCTT TCCAGTCGGG AAACCTGTCG TGCCAGCTGC ATTAATGAAT

2701 CGGCCAACGC GCGGGGAGAG GCGGTTTGCG TATTGGCTAG AGCAGCTTGC CAACATGGTG

2761 GAGCACGACA CTCTCGTCTA CTCCAAGAAT ATCAAAGATA CAGTCTCAGA AGACCAAAGG

2821 GCTATTGAGA CTTTTCAACA AAGGGTAATA TCGGGAAACC TCCTCGGATT CCATTGCCCA

2881 GCTATCTGTC ACTTCATCAA AAGGACAGTA GAAAAGGAAG GTGGCACCTA CAAATGCCAT

2941 CATTGCGATA AAGGAAAGGC TATCGTTCAA GATGCCTCTG CCGACAGTGG TCCCAAAGAT

3001 GGACCCCCAC CCACGAGGAG CATCGTGGAA AAAGAAGACG TTCCAACCAC GTCTTCAAAG

3061 CAAGTGGATT GATGTGATAA CATGGTGGAG CACGACACTC TCGTCTACTC CAAGAATATC

3121 AAAGATACAG TCTCAGAAGA CCAAAGGGCT ATTGAGACTT TTCAACAAAG GGTAATATCG

3181 GGAAACCTCC TCGGATTCCA TTGCCCAGCT ATCTGTCACT TCATCAAAAG GACAGTAGAA

3241 AAGGAAGGTG GCACCTACAA ATGCCATCAT TGCGATAAAG GAAAGGCTAT CGTTCAAGAT

3301 GCCTCTGCCG ACAGTGGTCC CAAAGATGGA CCCCCACCCA CGAGGAGCAT CGTGGAAAAA

3361 GAAGACGTTC CAACCACGTC TTCAAAGCAA GTGGATTGAT GTGATATCTC CACTGACGTA

3421 AGGGATGACG CACAATCCCA CTATCCTTCG CAAGACCTTC CTCTATATAA GGAAGTTCAT

3481 TTCATTTGGA GAGGACACGC TGAAATCACC AGTCTCTCTC TACAAATCTA TCTCTCTCGA

3541 GCTTTCGCAG ATCTGTCGAT CGACCATGGG GATTGAACAA GATGGATTGC ACGCAGGTTC

3601 TCCGGCCGCT TGGGTGGAGA GGCTATTCGG CTATGACTGG GCACAACAGA CAATCGGCTG

3661 CTCTGATGCC GCCGTGTTCC GGCTGTCAGC GCAGGGGCGC CCGGTTCTTT TTGTCAAGAC

3721 CGACCTGTCC GGTGCCCTGA ATGAACTCCA GGACGAGGCA GCGCGGCTAT CGTGGCTGGC

3781 CACGACGGGC GTTCCTTGCG CAGCTGTGCT CGACGTTGTC ACTGAAGCGG GAAGGGACTG

3841 GCTGCTATTG GGCGAAGTGC CGGGGCAGGA TCTCCTGTCA TCTCACCTTG CTCCTGCCGA

3901 GAAAGTATCC ATCATGGCTG ATGCAATGCG GCGGCTGCAT ACGCTTGATC CGGCTACCTG

3961 CCCATTCGAC CACCAAGCGA AACATCGCAT CGAGCGAGCA CGTACTCGGA TGGAAGCCGG

4021 TCTTGTCGAT CAGGATGATC TGGACGAAGA GCATCAGGGG CTCGCGCCAG CCGAACTGTT

4081 CGCCAGGCTC AAGGCGCGCA TGCCCGACGG CGAGGATCTC GTCGTGACAC ATGGCGATGC

4141 CTGCTTGCCG AATATCATGG TGGAAAATGG CCGCTTTTCT GGATTCATCG ACTGTGGCCG

4201 GCTGGGTGTG GCGGACCGCT ATCAGGACAT AGCGTTGGCT ACCCGTGATA TTGCTGAAGA

4261 GCTTGGCGGC GAATGGGCTG ACCGCTTCCT CGTGCTTTAC GGTATCGCCG CTCCCGATTC

4321 GCAGCGCATC GCCTTCTATC GCCTTCTTGA CGAGTTCTTC TGAGCGGGAC TCTGGGGTTC

4381 GGATCGATCC TCTAGCTAGA GTCGATCGAC AAGCTCGAGT TTCTCCATAA TAATGTGTGA

4441 GTAGTTCCCA GATAAGGGAA TTAGGGTTCC TATAGGGTTT CGCTCATGTG TTGAGCATAT

4501 AAGAAACCCT TAGTATGTAT TTGTATTTGT AAAATACTTC TATCAATAAA ATTTCTAATT

4561 CCTAAAACCA AAATCCAGTA CTAAAATCCA GATCCCCCGA ATTAATTCGG CGTTAATTCA

4621 GTACATTAAA AACGTCCGCA ATGTGTTATT AAGTTGTCTA AGCGTCAATT TGTTTACACC

4681 ACAATATATG GCGCGCCACC GGTGGTCTCT TCGGACTAGT CCTGGTTTAA ACCACCTGCA

4741 GGCTTAAGAT TGAATCCTGT TGCCGGTCTT GCGATGATTA TCATATAATT TCTGTTGAAT

4801 TACGTTAAGC ATGTAATAAT TAACATGTAA TGCATGACGT TATTTATGAG ATGGGTTTTT

4861 ATGATTAGAG TCCCGCAATT ATACATTTAA TACGCGATAG AAAACAAAAT ATAGCGCGCA

4921 AACTAGGATA AATTATCGCG CGCGGTGTCA TCTATGTTAC TAGATCGGGA ATTCACTGGC

4981 CGTCGTTTTA CAACGTCGTG ACTGGGAAAA CCCTGGCGTT ACCCAACTTA ATCGCCTTGC

5041 AGCACATCCC CCTTTCGCCA GCTGGCGTAA TAGCGAAGAG GCCCGCACCG ATCGCCCTTC

5101 CCAACAGTTG CGCAGCCTGA ATGGCGCCCG CTCCTTTCGC TTTCTTCCCT TCCTTTCTCG

5161 CCACGTTCGC CGGCTTTCCC CGTCAAGCTC TAAATCGGGG GCTCCCTTTA GGGTTCCGAT

5221 TTAGTGCTTT ACGGCACCTC GACCCCAAAA AACTTGATTT GGGTGATGGT TCACGTAGTG

5281 GGCCATCGCC CTGATAGACG GTTTTTCGCC CTTTGACGTT GGAGTCCACG TTCTTTAATA

5341 GTGGACTCTT GTTCCAAACT GGAACAACAC TCAACCCTAT CTCGGGCTAT TCTTTTGATT

5401 TATAAGGGAT TTTGCCGATT TCGGAACCAC CATCAAACAG GATTTTCGCC TGCTGGGGCA

5461 AACCAGCGTG GACCGCTTGC TGCAACTCTC TCAGGGCCAG GCGGTGAAGG GCAATCAGCT

5521 GTTGCCCGTC TCACTGGTGA AAAGAAAAAC CACCCCAGTA CATTAAAAAC GTCCGCAATG

5581 TGTTATTAAG TTGTCTAAGC GTCAATTTGT TTACACCACA ATATATCCTG CCACCAGCCA

5641 GCCAACAGCT CCCCGACCGG CAGCTCGGCA CAAAATCACC ACTCGATACA GGCAGCCCAT

5701 CAGTCCGGGA CGGCGTCAGC GGGAGAGCCG TTGTAAGGCG GCAGACTTTG CTCATGTTAC

5761 CGATGCTATT CGGAAGAACG GCAACTAAGC TGCCGGGTTT GAAACACGGA TGATCTCGCG

5821 GAGGGTAGCA TGTTGATTGT AACGATGACA GAGCGTTGCT GCCTGTGATC AAATATCATC

5881 TCCCTCGCAG AGATCCGAAT TATCAGCCTT CTTATTCATT TCTCGCTTAA CCGTGACAGG

5941 CTGTCGATCT TGAGAACTAT GCCGACATAA TAGGAAATCG CTGGATAAAG CCGCTGAGGA

6001 AGCTGAGTGG CGCTATTTCT TTAGAAGTGA ACGTTGACGA TATCAACTCC CCTATCCATT

6061 GCTCACCGAA TGGTACAGGT CGGGGACCCG AAGTTCCGAC TGTCGGCCTG ATGCATCCCC

6121 GGCTGATCGA CCCCAGATCT GGGGCTGAGA AAGCCCAGTA AGGAAACAAC TGTAGGTTCG

6181 AGTCGCGAGA TCCCCCGGAA CCAAAGGAAG TAGGTTAAAC CCGCTCCGAT CAGGCCGAGC

6241 CACGCCAGGC CGAGAACATT GGTTCCTGTA GGCATCGGGA TTGGCGGATC AAACACTAAA

6301 GCTACTGGAA CGAGCAGAAG TCCTCCGGCC GCCAGTTGCC AGGCGGTAAA GGTGAGCAGA

6361 GGCACGGGAG GTTGCCACTT GCGGGTCAGC ACGGTTCCGA ACGCCATGGA AACCGCCCCC

6421 GCCAGGCCCG CTGCGACGCC GACAGGATCT AGCGCTGCGT TTGGTGTCAA CACCAACAGC

6481 GCCACGCCCG CAGTTCCGCA AATAGCCCCC AGGACCGCCA TCAATCGTAT CGGGCTACCT

6541 AGCAGAGCGG CAGAGATGAA CACGACCATC AGCGGCTGCA CAGCGCCTAC CGTCGCCGCG

6601 ACCCCGCCCG GCAGGCGGTA GACCGAAATA AACAACAAGC TCCAGAATAG CGAAATATTA

6661 AGTGCGCCGA GGATGAAGAT GCGCATCCAC CAGATTCCCG TTGGAATCTG TCGGACGATC

6721 ATCACGAGCA ATAAACCCGC CGGCAACGCC CGCAGCAGCA TACCGGCGAC CCCTCGGCCT

6781 CGCTGTTCGG GCTCCACGAA AACGCCGGAC AGATGCGCCT TGTGAGCGTC CTTGGGGCCG

6841 TCCTCCTGTT TGAAGACCGA CAGCCCAATG ATCTCGCCGT CGATGTAGGC GCCGAATGCC

6901 ACGGCATCTC GCAACCGTTC AGCGAACGCC TCCATGGGCT TTTTCTCCTC GTGCTCGTAA

6961 ACGGACCCGA ACATCTCTGG AGCTTTCTTC AGGGCCGACA ATCGGATCTC GCGGAAATCC

7021 TGCACGTCGG CCGCTCCAAG CCGTCGAATC TGAGCCTTAA TCACAATTGT CAATTTTAAT

7081 CCTCTGTTTA TCGGCAGTTC GTAGAGCGCG CCGTGCGTCC CGAGCGATAC TGAGCGAAGC

7141 AAGTGCGTCG AGCAGTGCCC GCTTGTTCCT GAAATGCCAG TAAAGCGCTG GCTGCTGAAC

7201 CCCCAGCCGG AACTGACCCC ACAAGGCCCT AGCGTTTGCA ATGCACCAGG TCATCATTGA

7261 CCCAGGCGTG TTCCACCAGG CCGCTGCCTC GCAACTCTTC GCAGGCTTCG CCGACCTGCT

7321 CGCGCCACTT CTTCACGCGG GTGGAATCCG ATCCGCACAT GAGGCGGAAG GTTTCCAGCT

7381 TGAGCGGGTA CGGCTCCCGG TGCGAGCTGA AATAGTCGAA CATCCGTCGG GCCGTCGGCG

7441 ACAGCTTGCG GTACTTCTCC CATATGAATT TCGTGTAGTG GTCGCCAGCA AACAGCACGA

7501 CGATTTCCTC GTCGATCAGG ACCTGGCAAC GGGACGTTTT CTTGCCACGG TCCAGGACGC

7561 GGAAGCGGTG CAGCAGCGAC ACCGATTCCA GGTGCCCAAC GCGGTCGGAC GTGAAGCCCA

7621 TCGCCGTCGC CTGTAGGCGC GACAGGCATT CCTCGGCCTT CGTGTAATAC CGGCCATTGA

7681 TCGACCAGCC CAGGTCCTGG CAAAGCTCGT AGAACGTGAA GGTGATCGGC TCGCCGATAG

7741 GGGTGCGCTT CGCGTACTCC AACACCTGCT GCCACACCAG TTCGTCATCG TCGGCCCGCA

7801 GCTCGACGCC GGTGTAGGTG ATCTTCACGT CCTTGTTGAC GTGGAAAATG ACCTTGTTTT

7861 GCAGCGCCTC GCGCGGGATT TTCTTGTTGC GCGTGGTGAA CAGGGCAGAG CGGGCCGTGT

7921 CGTTTGGCAT CGCTCGCATC GTGTCCGGCC ACGGCGCAAT ATCGAACAAG GAAAGCTGCA

7981 TTTCCTTGAT CTGCTGCTTC GTGTGTTTCA GCAACGCGGC CTGCTTGGCC TCGCTGACCT

8041 GTTTTGCCAG GTCCTCGCCG GCGGTTTTTC GCTTCTTGGT CGTCATAGTT CCTCGCGTGT

8101 CGATGGTCAT CGACTTCGCC AAACCTGCCG CCTCCTGTTC GAGACGACGC GAACGCTCCA

8161 CGGCGGCCGA TGGCGCGGGC AGGGCAGGGG GAGCCAGTTG CACGCTGTCG CGCTCGATCT

8221 TGGCCGTAGC TTGCTGGACC ATCGAGCCGA CGGACTGGAA GGTTTCGCGG GGCGCACGCA

8281 TGACGGTGCG GCTTGCGATG GTTTCGGCAT CCTCGGCGGA AAACCCCGCG TCGATCAGTT

8341 CTTGCCTGTA TGCCTTCCGG TCAAACGTCC GATTCATTCA CCCTCCTTGC GGGATTGCCC

8401 CGACTCACGC CGGGGCAATG TGCCCTTATT CCTGATTTGA CCCGCCTGGT GCCTTGGTGT

8461 CCAGATAATC CACCTTATCG GCAATGAAGT CGGTCCCGTA GACCGTCTGG CCGTCCTTCT

8521 CGTACTTGGT ATTCCGAATC TTGCCCTGCA CGAATACCAG CGACCCCTTG CCCAAATACT

8581 TGCCGTGGGC CTCGGCCTGA GAGCCAAAAC ACTTGATGCG GAAGAAGTCG GTGCGCTCCT

8641 GCTTGTCGCC GGCATCGTTG CGCCACATCT AGGTACTAAA ACAATTCATC CAGTAAAATA

8701 TAATATTTTA TTTTCTCCCA ATCAGGCTTG ATCCCCAGTA AGTCAAAAAA TAGCTCGACA

8761 TACTGTTCTT CCCCGATATC CTCCCTGATC GACCGGACGC AGAAGGCAAT GTCATACCAC

8821 TTGTCCGCCC TGCCGCTTCT CCCAAGATCA ATAAAGCCAC TTACTTTGCC ATCTTTCACA

8881 AAGATGTTGC TGTCTCCCAG GTCGCCGTGG GAAAAGACAA GTTCCTCTTC GGGCTTTTCC

8941 GTCTTTAAAA AATCATACAG CTCGCGCGGA TCTTTAAATG GAGTGTCTTC TTCCCAGTTT

9001 TCGCAATCCA CATCGGCCAG ATCGTTATTC AGTAAGTAAT CCAATTCGGC TAAGCGGCTG

9061 TCTAAGCTAT TCGTATAGGG ACAATCCGAT ATGTCGATGG AGTGAAAGAG CCTGATGCAC

9121 TCCGCATACA GCTCGATAAT CTTTTCAGGG CTTTGTTCAT CTTCATACTC TTCCGAGCAA

9181 AGGACGCCAT CGGCCTCACT CATGAGCAGA TTGCTCCAGC CATCATGCCG TTCAAAGTGC

9241 AGGACCTTTG GAACAGGCAG CTTTCCTTCC AGCCATAGCA TCATGTCCTT TTCCCGTTCC

9301 ACATCATAGG TGGTCCCTTT ATACCGGCTG TCCGTCATTT TTAAATATAG GTTTTCATTT

9361 TCTCCCACCA GCTTATATAC CTTAGCAGGA GACATTCCTT CCGTATCTTT TACGCAGCGG

9421 TATTTTTCGA TCAGTTTTTT CAATTCCGGT GATATTCTCA TTTTAGCCAT TTATTATTTC

9481 CTTCCTCTTT TCTACAGTAT TTAAAGATAC CCCAAGAAGC TAATTATAAC AAGACGAACT

9541 CCAATTCACT GTTCCTTGCA TTCTAAAACC TTAAATACCA GAAAACAGCT TTTTCAAAGT

9601 TGTTTTCAAA GTTGGCGTAT AACATAGTAT CGACGGAGCC GATTTTGAAA CCACAATTAT

9661 GGGTGATGCT GCCAACTTAC TGATTTAGTG TATGATGGTG TTTTTGAGGT GCTCCAGTGG

9721 CTTCTGTGTC TATCAGCTGT CCCTCCTGTT CAGCTACTGA CGGGGTGGTG CGTAACGGCA

9781 AAAGCACCGC CGGACATCAG CGCTATCTCT GCTCTCACTG CCGTAAAACA TGGCAACTGC

9841 AGTTCACTTA CACCGCTTCT CAACCCGGTA CGCACCAGAA AATCATTGAT ATGGCCATGA

9901 ATGGCGTTGG ATGCCGGGCA ACAGCCCGCA TTATGGGCGT TGGCCTCAAC ACGATTTTAC

9961 GTCACTTAAA AAACTCAGGC CGCAGTCGGT AACCTCGCGC ATACAGCCGG GCAGTGACGT

10021 CATCGTCTGC GCGGAAATGG ACGAACAGTG GGGCTATGTC GGGGCTAAAT CGCGCCAGCG

10081 CTGGCTGTTT TACGCGTATG ACAGTCTCCG GAAGACGGTT GTTGCGCACG TATTCGGTGA

10141 ACGCACTATG GCGACGCTGG GGCGTCTTAT GAGCCTGCTG TCACCCTTTG ACGTGGTGAT

10201 ATGGATGACG GATGGCTGGC CGCTGTATGA ATCCCGCCTG AAGGGAAAGC TGCACGTAAT

10261 CAGCAAGCGA TATACGCAGC GAATTGAGCG GCATAACCTG AATCTGAGGC AGCACCTGGC

10321 ACGGCTGGGA CGGAAGTCGC TGTCGTTCTC AAAATCGGTG GAGCTGCATG ACAAAGTCAT

10381 CGGGCATTAT CTGAACATAA AACACTATCA ATAAGTTGGA GTCATTACCC AATTATGATA

10441 GAATTTACAA GCTATAAGGT TATTGTCCTG GGTTTCAAGC ATTAGTCCAT GCAAGTTTTT

10501 ATGCTTTGCC CATTCTATAG ATATATTGAT AAGCGCGCTG CCTATGCCTT GCCCCCTGAA

10561 ATCCTTACAT ACGGCGATAT CTTCTATATA AAAGATATAT TATCTTATCA GTATTGTCAA

10621 TATATTCAAG GCAATCTGCC TCCTCATCCT CTTCATCCTC TTCGTCTTGG TAGCTTTTTA

10681 AATATGGCGC TTCATAGAGT AATTCTGTAA AGGTCCAATT CTCGTTTTCA TACCTCGGTA

10741 TAATCTTACC TATCACCTCA AATGGTTCGC TGGGTTTATC GCACCCCCGA ACACGAGCAC

10801 GGCACCCGCG ACCACTATGC CAAGAATGCC CAAGGTAAAA ATTGCCGGCC CCGCCATGAA

10861 GTCCGTGAAT GCCCCGACGG CCGAAGTGAA GGGCAGGCCG CCACCCAGGC CGCCGCCCTC

10921 ACTGCCCGGC ACCTGGTCGC TGAATGTCGA TGCCAGCACC TGCGGCACGT CAATGCTTCC

10981 GGGCGTCGCG CTCGGGCTGA TCGCCCATCC CGTTACTGCC CCGATCCCGG CAATGGCAAG

11041 GACTGCCAGC GCTGCCATTT TTGGGGTGAG GCCGTTCGCG GCCGAGGGGC GCAGCCCCTG

11101 GGGGGATGGG AGGCCCGCGT TAGCGGGCCG GGAGGGTTCG AGAAGGGGGG GCACCCCCCT

11161 TCGGCGTGCG CGGTCACGCG CACAGGGCGC AGCCCTGGTT AAAAACAAGG TTTATAAATA

11221 TTGGTTTAAA AGCAGGTTAA AAGACAGGTT AGCGGTGGCC GAAAAACGGG CGGAAACCCT

11281 TGCAAATGCT GGATTTTCTG CCTGTGGACA GCCCCTCAAA TGTCAATAGG TGCGCCCCTC

11341 ATCTGTCAGC ACTCTGCCCC TCAAGTGTCA AGGATCGCGC CCCTCATCTG TCAGTAGTCG

11401 CGCCCCTCAA GTGTCAATAC CGCAGGGCAC TTATCCCCAG GCTTGTCCAC ATCATCTGTG

11461 GGAAACTCGC GTAAAATCAG GCGTTTTCGC CGATTTGCGA GGCTGGCCAG CTCCACGTCG

11521 CCGGCCGAAA TCGAGCCTGC CCCTCATCTG TCAACGCCGC GCCGGGTGAG TCGGCCCCTC

11581 AAGTGTCAAC GTCCGCCCCT CATCTGTCAG TGAGGGCCAA GTTTTCCGCG AGGTATCCAC

11641 AACGCCGGCG GCCGCGGTGT CTCGCACACG GCTTCGACGG CGTTTCTGGC GCGTTTGCAG

11701 GGCCATAGAC GGCCGCCAGC CCAGCGGCGA GGGCAACCAG CCCGG
