# Supplementary material for: TALEN-Based Mutagenesis of Lipoxygenase LOX3 Enhances the Storage Tolerance of Rice (Oryza sativa) Seeds
Source: PLoS One. 2015 Dec 7;10(12):e0143877. doi: 10.1371/journal.pone.0143877 (PMC4671593; doi:10.1371/journal.pone.0143877)
Supplement: S2 File — (DOCX) [file pone.0143877.s003.docx]

**Supplemental materials and methods**

**Construction of vector system**

The backbone of the donor vectors used in this study was derived from pMD18-T simple vector and was further optimized. The two Esp3I sites of pMD18-T simple vector were mutated by Fast Mutagenesis System (TransGen Biotech) using primers mutz5, muty5, mutz6 and muty6. The synthesis DNA fragment (5’-TTAATTAAGGGGGGTTACCGGGTACCCCAAGCTTCGGCG

CGCCTTACGCGTCGGTAAATCATCTTAATTAA-3’) was introduced into modified pMD18-T simple vector to obtain pMAL01. Similarly, the DNA fragment (5’-TTAATTAAATCATCTTAGG

GGGTACCCCAAGCTTCGGGCGCGCCTTACGCGTCCCTAATAATAATTAATTAA-3’) was also introduced into modified pMD18-T simple vector to obtain pMAL02. To obtain TALEN expression cassette, the AscI was added to multicloning site (MCS) of pBluescript II SK, and the PCR product of maize ubiquitous promoter from pCXUN (primers ubiz and ubiy) was cloned and inserted into KpnI and SmaI sites, generating clone pBluescriptII SK-Ubi promoter. Then the nos terminator was cloned (Tnosz, Tnosy) into SacI and AscI sites of pBluescript II SK-Ubi promoter to obtain pBluescriptII SK-Ubi promoter-nos terminator. For cloning convenience for repeat restriction enzymes, LaZ fragment in TALE scaffold is replaced with eGFP, which has no effect on the application of the vector. The TALEN scaffold was digested with XhoI and SacI from pTAL1, and inserted into corresponding sites of pBluescriptII SK-Ubi promoter-nos terminator to obtain pBluescriptII SK-Ubi promoter-TAL-nos terminator. Also, CaMV35S promoter(35z1, 35y1) or double-enhancer version of the CaMV35S promoter(35z2, 35y2) was PCR-amplified and cloned into corresponding positions of pBluescriptII SK-Ubi promoter-TAL-nos terminator, producing pBluescriptII SK-P35S(2*p35S) promoter-TAL-nos terminator. Then, the TALEN expression cassette was digested with KpnI and AscI and introduced into pMAL01 and pMAL02.

The GoldyTALEN scaffold was taken from RCIscript-GoldyTALEN (Addgene #38142), and heterodimeric (ELD, KKR mutations) FokI domains were mutated by Fast Mutagenesis System (TransGen Biotech) using primers mutz1, muty1, mutz2, muty2 mutz3, muty3, mutz4 and muty4.

To produce pTALEN-DES01/02, the DNA fragment:

(1)AgeI-FS-PacI-FS-SpeI 5’-AATTCACCGGTCCTAAGGGGGGTTAATTAATAATAATTAGGACTAGTCA-3’

and

(2)SpeI-FS-PacI-FS-SbfI

5’- AATTCACTAGTCCTAAGGGGGGTTAATTAATAATAATTAGGCCTGCAGGCA-3’

were synthesized and inserted into pCambia2300 by EcoRI and HindIII.

In addition, the DNA fragment AgeI-BsaI-SpeI-PmeI-SbfI:

5’- CGCGCCACCGGTGGTCTCTTCGGACTAGTCCTGGTTTAAACCACCTGCAGGC-3’was blunt-end –cloned into the HindIII site of pBI121 to obtain pTALEN-DES03.

The GFP report plasmid in SSA-mediated yeast assay was made based on pGBKT7 (Matchmaker® Gold Yeast Two-Hybrid System, clontech). Two partially overlapped GFP fragments amplified(primers gfpz2, gfpy2, gfpz3 and gfpy3) were introduced into pGBKT7 with AscI, PacI and SbfI between promoter ADH1 and terminator, yielding pGBKT7-DES. The target site of LOX-T1 and LOX-T2, LOX-T3 and LOX-T4, LOX-T5 and LOX-T6 were synthesized and inserted into corresponding sites of pGBKT7-DES.
