# Supplementary material for: TALEN-Based Mutagenesis of Lipoxygenase LOX3 Enhances the Storage Tolerance of Rice (Oryza sativa) Seeds
Source: PLoS One. 2015 Dec 7;10(12):e0143877. doi: 10.1371/journal.pone.0143877 (PMC4671593; doi:10.1371/journal.pone.0143877)
Supplement: S1 Table — (DOCX) [file pone.0143877.s004.docx]

**S1 Table .**

**Information on engineered TALEN for lipoxygenase *LOX3***

| **TALEN ID** | **exon** | **Repeat number** | **Spacer length** | **RVDs** | **Target sequence** |
| --- | --- | --- | --- | --- | --- |
| **LOX-T1** | 4 | 20 | 19 | NN HD HD NN HD HD NN NN HD NN NI HD NG NI NG NI NG HD HD NG | GCCGCCGGCGACTATATCCT-caagctccccatgcctcag-ATTATCAAACGTGAGCGC |
| **LOX-T2** |  | 18 |  | NN HD NN HD NG HD NI HD NN NG NG NG NN NI NG NI NI NG |  |
|  |  |  |  |  |  |
| **LOX-T3** | 3 | 15 | 17 | NI HD HD NG NN HD HD NN NI NN HD HD NI NN NI | ACCTGCCGAGCCAGA-tgccggcggcgctgaagc-CGTACCGCGACGACG |
| **LOX-T4** |  | 15 |  | HD NN NG HD NN NG HD NN HD NN NN NG NI HD NN |  |
|  |  |  |  |  |  |
| **LOX-T5** | 4 | 20 | 20 | HD HD NN NN NI HD NG HD NN NN NI NN NI NN NG NI NN NN HD NG | CCGGACTCGGAGAGTAGGCT-gtcgctggtggagcagatct-ACGTGCCGCGGGACG |
| **LOX-T6** |  | 15 |  | HD NN NG HD HD HD NN HD NN NN HD NI HD NN NG |  |
