# Supplementary material for: TALEN-Based Mutagenesis of Lipoxygenase LOX3 Enhances the Storage Tolerance of Rice (Oryza sativa) Seeds
Source: PLoS One. 2015 Dec 7;10(12):e0143877. doi: 10.1371/journal.pone.0143877 (PMC4671593; doi:10.1371/journal.pone.0143877)
Supplement: S2 Table — (DOCX) [file pone.0143877.s005.docx]

**S2 Table .**

**List of PCR primers**

| name | 5’-3’ |
| --- | --- |
| mutz5 | GACACATGCAGCTCCCGGTCACGGTCAAGCG |
| muty5 | GACCGGGAGCTGCATGTGTCAGAGGTTTTCAC |
| mutz6 | TATCACGAGGCCCTTTCGAGTCGCGCGTTTC |
| muty6 | CTCGAAAGGGCCTCGTGATACGCCTATTTT |
| ubiz | GGTACCCTGCAGTGCAGCGTGACCC |
| ubiy | CCCGGGCTGCAGAAGTAACACCAAAC |
| Tnosz | CGAGCTCGGATCGTTCAAACATTTGGC |
| Tnosy | GGCGCGCCCGATCTAGTAACATAG |
| 35z1 | GGGGTACCCATGGAGTCAAAGATTCAAATAGAGG |
| 35y1 | CCCTCGAGAGTCCCCCGTGTTCTCTCC |
| 35z2 | GGTACCGCCTCCTGTCAATGCTG |
| 35y2 | CTCGAGCGTGTTCTCTCCAAATGAAA |
| mutz1 | GAAATGCAAAGATATGTCAAAGAGAATCA |
| muty1 | TGACATATCTTTGCATTTCATCTGCTTGA |
| mutz2 | CTTACAAGATTGAATCATAAGACTAATTGTA |
| muty2 | CTTATGATTCAATCTTGTAAGCTGAGCTTTG |
| mutz3 | GGTCAAGCAGATGAAATGGAAAGATATGT |
| muty3 | CCATTTCATCTGCTTGACCAATTGGAAGA |
| mutz4 | CAAACAAGAAACAAGCATCTCAACCCTAA |
| muty4 | GATGCTTGTTTCTTGTTTGATTCTCTTCG |
| gfpz2 | CCATGGCGCGCCAGTTAATTAACTACCTGCAGGTCGTAGATCTGACTAGTAAAGGAG |
| gfpy2 | GCGGCCGCTCACACGTGGTGGTGGTGG |
| gfpz3 | TCCATGGTAGATCTGACTAGTAAAG |
| gfpy3 | TTGGCGCGCCAATTAGTTGGCTTTGATGCCGTTC |
| kanz | AGGAACCCTAATTCCCTTATCTGG |
| kany | CCTGTCATCTCACCTTGCTCCTG |
| TALz | GGTTAGTTGGAAGGACGCAAGTGG |
| TALy | CCTCTGGCAACGCCGTGATTATG |
| FokIz | CTAGTGAAATCTGAATTGGAAGAGAAGAAATC |
| FokIy | CACCGTTATTAAATTTCCTTCTCACTTCCTC |
